# Decision-Making about Healthcare Related Tests and Diagnostic Strategies: User Testing of GRADE Evidence Tables

**DOI:** 10.1371/journal.pone.0134553

**Published:** 2015-10-16

**Authors:** Reem A. Mustafa, Wojtek Wiercioch, Nancy Santesso, Adrienne Cheung, Barbara Prediger, Tejan Baldeh, Alonso Carrasco-Labra, Romina Brignardello-Petersen, Ignacio Neumann, Patrick Bossuyt, Amit X. Garg, Monika Lelgemann, Diedrich Bühler, Jan Brozek, Holger J. Schünemann

**Affiliations:** 1 Department of Clinical Epidemiology and Biostatistics, McMaster University, Hamilton, Ontario, Canada; 2 Departments of Internal Medicine and Biomedical & Health Informatics, University of Missouri-Kansas City, Kansas City, United States of America; 3 Faculty of Medicine, University of British Colombia, Vancouver, British Columbia, Canada; 4 Center for Medical Biometry and Medical Informatics, University of Freiburg, Freiburg, Germany; 5 Evidence-Based Dentistry Unit, Faculty of Dentistry, Universidad de Chile, Santiago de Chile, Chile; 6 Institute of Health Policy, Management and Evaluation, University of Toronto, Toronto, Canada; 7 Department of Internal Medicine, Pontificia Universidad Católica de Chile, Santiago, Chile; 8 Department of Clinical Epidemiology, Biostatistics and Bioinformatics, Academic Medical Center, Amsterdam, The Netherlands; 9 Department of Medicine, Western University, London, Ontario, Canada; 10 Medizinischer Dienst des Spitzenverbandes Bund der Kranken-kassen e.V. (MDS) Theodor Althoff-Str. 47 45133 Essen, Germany; 11 Abteilung Medizin. GKV—Reinhardtstraße 28 10117 Berlin, Germany; 12 Department of Medicine, McMaster University, Hamilton, Ontario, Canada; University of Algarve, PORTUGAL

## Abstract

**Objective:**

To develop guidance on what information to include and how to present it in tables summarizing the evidence from systematic reviews of test accuracy following the Grading of Recommendations Assessment, Development and Evaluation (GRADE) approach.

**Methods:**

To design and refine the evidence tables, we used an iterative process based on the analysis of data from four rounds of discussions, feedback and user testing. During the final round, we conducted one-on-one user testing with target end users. We presented a number of alternative formats of evidence tables to participants and obtained information about users’ understanding and preferences.

**Results:**

More than 150 users participated in initial discussions and provided their formal and informal feedback. 20 users completed one-on-one user testing interviews. Almost all participants preferred summarizing the results of systematic reviews of test accuracy in tabular format rather than plain text. Users generally preferred less complex tables but found presenting sensitivity and specificity estimates only as too simplistic. Users found the presentation of test accuracy for several values of prevalence initially confusing but modifying table layout and adding sample clinical scenarios for each prevalence reduced this confusion. Providing information about clinical consequences of testing result was viewed as not feasible for authors of systematic reviews.

**Conclusion:**

We present the current formats for tables presenting test accuracy following the GRADE approach. These tables can be developed using GRADEpro guidelines development tool (www.guidelinedevelopment.org or www.gradepro.org) and are being further developed into electronic interactive tables that will suit the needs of different end users. The formatting of these tables, and how they influence result interpretation and decision-making will be further evaluated in a randomized trial.

## Background

The Grading of Recommendations Assessment, Development and Evaluation (GRADE) Working Group’s approach to assessing evidence and developing health care recommendations has been adopted by over 90 organizations worldwide, including the World Health Organization and the National Institute for Health and Care Excellence in the UK (www.gradeworkinggroup.org). A key GRADE output are evidence summaries (including summary of findings table and evidence profiles) that are intended to provide succinct, easily consumable information about intervention effects on the most important health outcomes for decision making, the quality of evidence (certainty or confidence in the effect estimates) and magnitude of such effects.[1, 2] In this article we describe research supporting the development and modifications of GRADE tables summarizing the evidence about test accuracy (TA) based on systematic reviews. We aim to present the key findings about users’ perspectives and feedback on various formats of diagnostic evidence tables. We also aim to provide an overview of the remaining challenges in presenting TA systematic review results for users in preparation for follow-up work in this area.

Systematic reviews and meta-analysis of TA summarize the available evidence and assess its quality (certainty or confidence in the effect estimates). TA systematic reviews typically focus on tests to establish the presence or absence of a disease, condition or syndrome, and on tests that ultimately categorize results as positive or negative.

Despite significant developments in the methodology of TA systematic reviews, authors still face many challenges, including how best to present their results to different users. The GRADE Working Group [3] has previously laid out its approach to making recommendations about diagnostic tests including existing challenges and limitations[4]. Appreciating these limitations among users including healthcare providers, guideline developers, and policymakers and their need for TA information in decision making led to the development of diagnostic evidence tables.

In this article, for simplicity of communication, “tests” refers to all healthcare related tests and diagnostic strategies that are used for different application and roles and not necessarily to make a diagnosis sensu stricto. Tests are used for many different applications: screening or surveillance, risk assessment and classification, diagnosis (ruling in), ruling out diagnosis, treatment triage, treatment monitoring, staging and determining prognosis. Tests are used in different roles in the care pathways: triage, add-on, replacement [5], parallel testing and replacement of a reference test[6].

## Methods

### Setting and design

**Fig 1:**
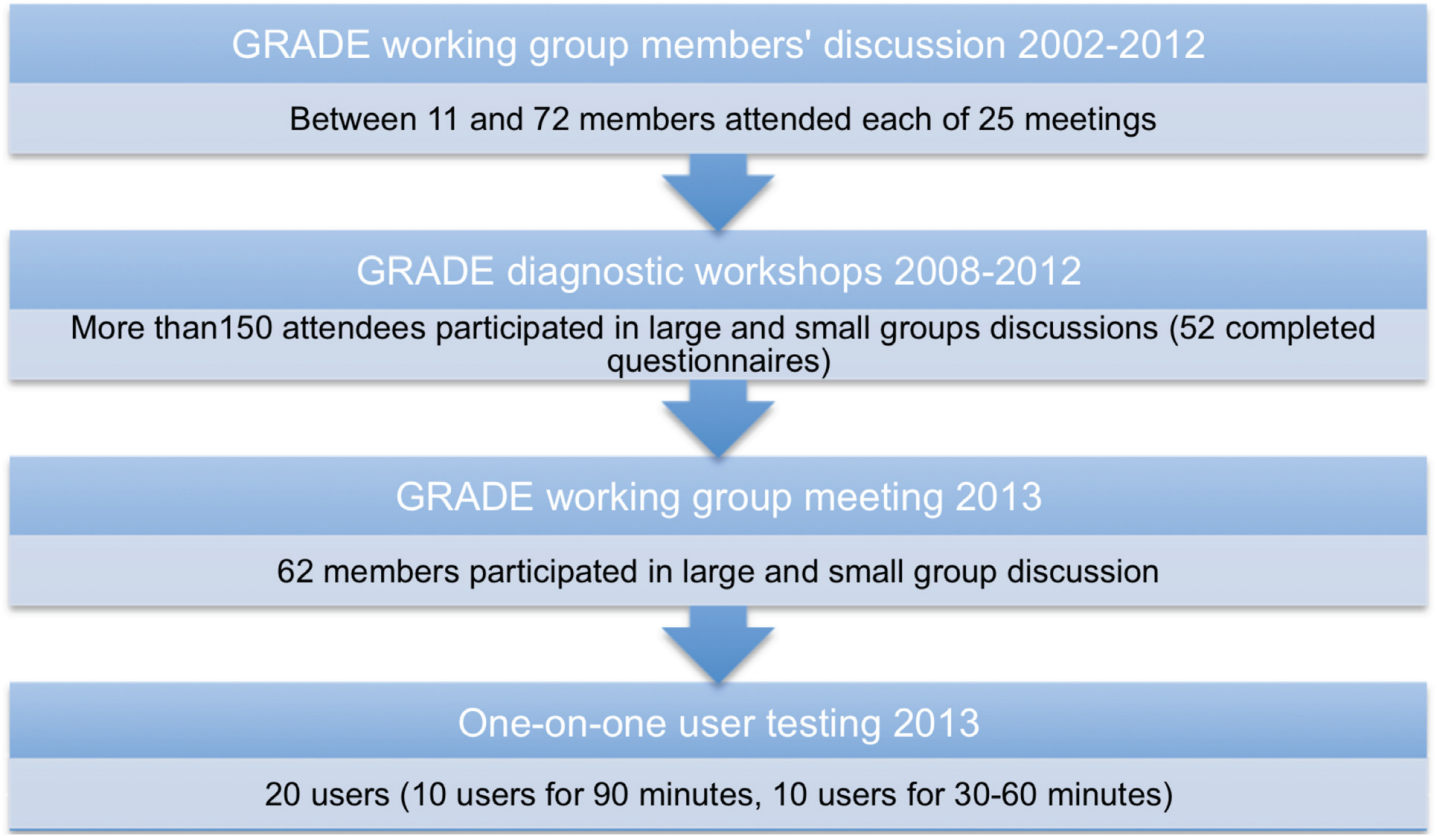
Outline of the rounds of feedback and user testing to develop GRADE diagnostic summary tables.

We evaluated a variety of formats of the diagnostic evidence tables. We used an iterative process to modify the diagnostic evidence tables based on analysis of data from each round of feedback and user testing. Fig 1 summarizes the different rounds that led to the current suggested formats.

The first round involved discussions among members of the GRADE working group, fueled by specific examples of systematic reviews and addressing how the results can be presented. The second round included collecting feedback from various stakeholders attending several diagnostic GRADE workshops. The feedback was obtained informally during the large and small group discussions as well as formally using specifically designed questionnaires. Appendix A in S1 Appendices presents an example of a work package used in one of such workshops. The third round involved several large and small group discussions at the GRADE working group meeting in Barcelona in January 2013. The fourth round consisted of formal one-on-one user testing either in person or via teleconferencing. Appendix B in S1 Appendices presents two examples of material used in the one-on-one user testing.

Initially we developed tables summarizing the information about the use of a single test; later on we explored ways of summarizing information from comparative TA reviews in which ≥2 tests are compared against each other and a common reference standard.

In this article we provide a summary of our final results after considering the feedback from all rounds. We summarise the finding of the background work and we focus on the results of the final round of user testing.

### Background work (rounds 1 and 2)

In the year 2000, the GRADE working group was established to address the shortcomings of existing systems for grading quality of evidence (certainty or confidence in the effect estimates) and strength of recommendations in health care. The working group has met regularly 2–3 times a year to develop the GRADE approach and to address methodological challenges in assessing quality of the evidence and strength of recommendations for health care questions including preventive, therapeutic and diagnostic interventions. In 2002, HJS and JB began with the development of SoF tables for TA and then prepared and facilitated workshops with input from other GRADE members aimed at training and informing various users of TA systematic reviews representing many organizations and professional groups. Each of 12 GRADE diagnostic workshops consisted of a brief presentation about TA results and applying the GRADE approach to evaluate the quality of evidence, a small group exercise to work through practical examples of TA systematic reviews, and the presentation and evaluation of results in diagnostic evidence tables. Workshops duration ranged between 1.5 hours to two days. Workshops were conducted in various countries, including Canada, Germany, Italy, Singapore, Spain, Switzerland and the United States.

#### Test evidence tables

Test evidence tables include three main parts: a heading, the body of the table and the footnotes. The heading contains a description of the health question in a PICO format (population, intervention, comparison and outcomes) and specifications of the index test(s) and the reference test of interest. Similar to the tables used to present results of therapeutic interventions, [1, 2] the body of the table summarizes the results of a systematic review of test accuracy and the judgements about the quality of the evidence (certainty or confidence in the effect estimates). Footnotes provide explanations for specific issues in the body of the table, including justification for any judgements made, e.g. about quality of evidence.

There are two main types of GRADE evidence tables: the evidence profile (EvP) and summary of findings (SoF) tables. The EvP reports the estimates for test accuracy measures and the detailed judgements about the five domains for rating the quality of evidence—considerations of risk of bias, indirectness, inconsistency, imprecision, publication bias, and others. The SoF table follows a similar format. However, it is less complex as detailed judgements about each domain of quality of evidence being explained only in footnotes. Appendix C in S1 Appendices presents an example of GRADE EvP and SoF tables summarizing test accuracy data.

### Participants

Between 11 and 72 members attended each of 25 GRADE working group meetings between 2002 and 2012. More than 150 stakeholders participated in large and small group discussions during workshops and 52 of them completed formal feedback questionnaires about GRADE diagnostic evidence tables. 62 members participated in large and small group discussions and feedback in GRADE working group meetings in 2013. Twenty participants completed one-on-one user testing interviews (10 for 90 minutes and 10 for 30–60 minutes).

We recruited participants, for one-on-one user testing, by sending electronic invitations to authors of Cochrane TA systematic reviews and participants in GRADE workshops at the Guideline International network meeting in 2013. In addition, we approached a convenient sample of clinicians at McMaster University who use results of DTA systematic reviews to inform their clinical decisions. Participants had broad range of experience with TA systematic reviews, health research methods and GRADE. We surveyed and collected feedback from an international group of authors of TA systematic reviews, methodologists, guideline developers, policy makers, and health care professionals that addressed, developed or used systematic reviews or recommendations about tests. [7] Table 1 summarizes background characteristics of the 20 participants who volunteered to complete one-on-one user testing.

**Table 1:** Background characteristics of participants in one on one user testing.

Characteristic	Response (n = 20)
Researcher	80%
Health Professional	60%
Guideline Developer *	60%
Author of DTA systematic review(s)	50%
Years of experience	Mean: 8.5 years (SD 7.52) Range: 1–33 years
Familiarity with GRADE (7 point likert scale)	Mean: 5.9 (SD 1.14) Range: 3–7
Familiarity with GRADE SoF tables (7 point likert scale)	Mean: 6 (SD 1.47) Range: 1–7

* This question was only asked to 10 participants

### Study design and data collection

**Fig 2:**
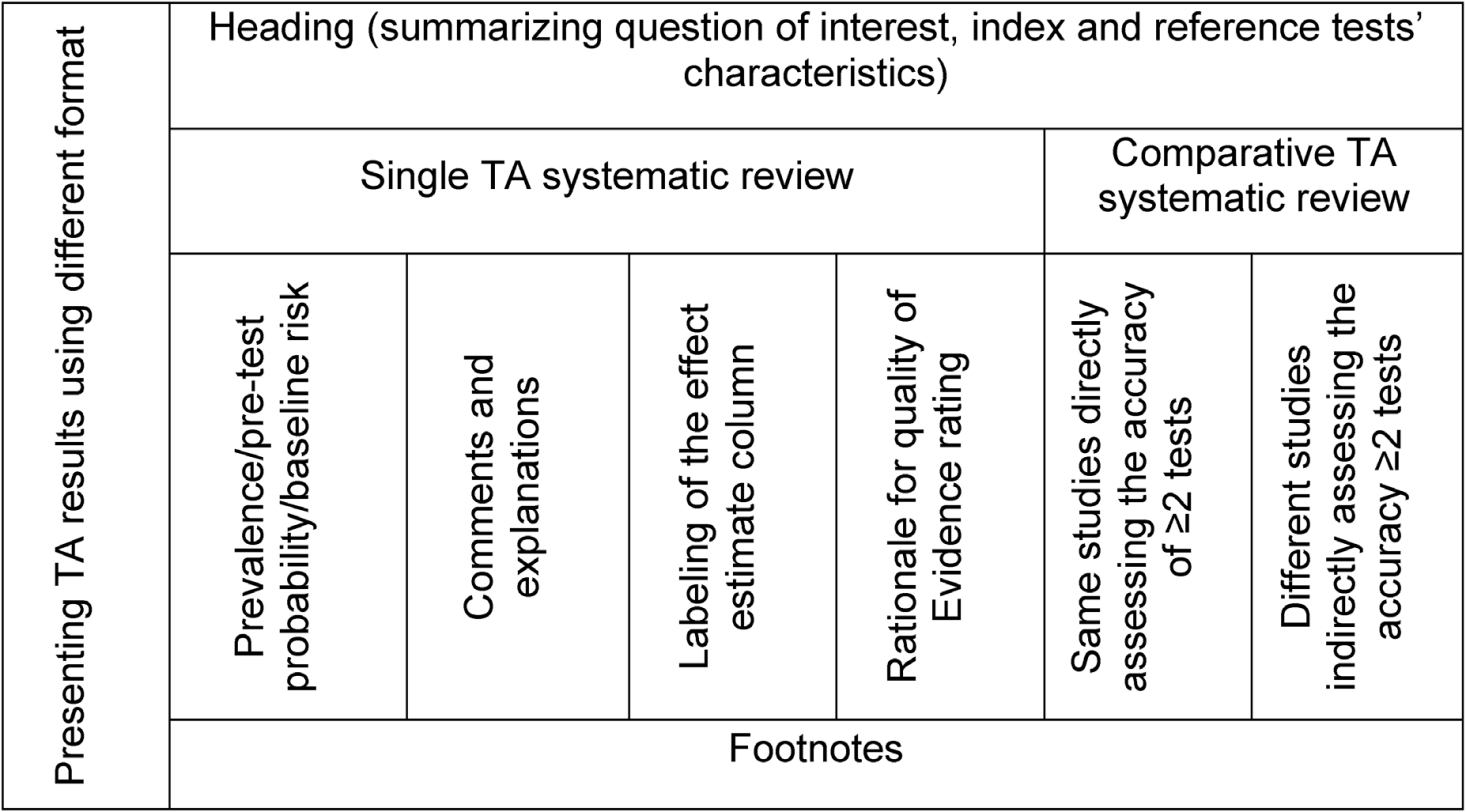
Summary of the domains used for data analysis of user testing and feedback.

The formal one-on-one user testing specifically was intended to compare various formats of evidence tables and to collect user perspectives about the most useful and best possible presentation of information in tables. The results were summarized for TA systematic reviews of single tests and multiple tests that were compared either directly in the same studies or indirectly in different studies against the same reference standard. We used the domains summarized in Fig 2 for our data analysis. We used the different table components as our guide to compile feedback. We also analyzed the comments that addressed the single test versus those that addressed comparative tests separately.

### Data analysis

Two investigators (RAM and WW) separately reviewed the notes and transcripts of participants’ comments and results of user testing and then discussed their findings. We gathered users’ views on the presentation and formatting, content, comprehensiveness, usefulness and accessibility of results in the evidence tables. We also analyzed reasons for confusion and misunderstanding related to the evidence tables’ content. We used basic content data analysis using a data coding system that corresponds to the data collection. We used conventional content analysis as the goal of our study was descriptive and there is little existing theory to guide our analysis. [8] We used a deductive coding to summarise the finding under each of the domains highlighted in Fig 2. Before we carried out the next round of user testing we modified the tables based on the findings of the previous round.

### Ethics statement

The research focusing on user testing was reviewed and approved by the Hamilton integrated Research Ethics Board.

## Results

A range between 11 and 72 members attended each of 25 GRADE working group meetings between 2002 and 2012. More than 150 stakeholders participated in large and small group discussions during workshops and 52 of them completed formal feedback questionnaires about GRADE diagnostic evidence tables. 62 members participated in large and small group discussions and feedback in GRADE working group meetings in 2013 and 20 participants completed one on one user testing interviews (10 for 90 minutes and 10 for 30–60 minutes).

### Presenting TA results using different format

Almost all participants preferred summarizing the results of TA systematic reviews in table format. They considered evidence tables as useful and easy to follow. During the rounds of collecting feedback and user testing we assessed four main formats of tables presenting: 1. sensitivity and specificity estimates only, **2**. individual TA numbers (true positives (TP), false positive (FP), true negative (TN) and false negative (FN)) organised based on test results (test positive and negative), **3**. individual TA numbers (TP, FN, FP and TN) organised based on disease status (disease present or absent), and 4. likelihood ratios with pre- and post-test probabilities.

#### Sensitivity and specificity alone (format 1)

In early discussions some participants noted that a simple format including only sensitivity and specificity would be sufficient. However, once we tested this simplest format, participants unanimously noted that they did not prefer it. Participants noted that sensitivity and specificity are parameters of the test that are familiar to most users, but they are often misinterpreted and may not reflect well the effects expected in the population of interest. Participants also noted that this simple table is missing critical information including estimates of prevalence and other measures of test accuracy such as likelihood ratios, predictive values, and absolute numbers of TP, FP, TN and FN that may be more useful for decision-making. Hence, later rounds focused on the other three formats of the tables.

#### Individual TA values—TP, TN, FP and FN (formats 2 and 3)

Participants generally liked this format but did not have a clear preference for arranging TP, TN, FP and FN in any specific order. Some noted that arranging the rows by test positive (TP and FP) and test negative (TN, FN) makes it more difficult to make a link between the individual test result values and the sensitivity and specificity which are the direct results of a systematic review as they are usually combined across studies in meta-analysis. However, views about what is the more useful order for users varied. Some noted that clinicians are accustomed to thinking about positive and negative test results and that this order may be useful to them. Others noted that arranging the outcomes according to disease status (disease positive (TP and FN) and disease negative (TN and FP)) and making the link to sensitivity and specificity in the table helps better highlight the results, which is most typically used by clinicians and decision makers when applying the test on a population level.

#### Likelihood ratios with pre- and post-test probability (format 4)

Some respondents noted that this format represents clinicians’ implicit thinking in terms of changes in post-test probability based on a test result. Others noted that this format with likelihood ratios and probabilities is a more difficult format to use and takes more time to interpret, especially for those who are not familiar with accuracy measures. Some respondents suggested that it is easier to think of patients and test results in absolute numbers, rather than in changes in probability. It was noted that in this table format, users would most likely use the post-test probability to make decisions about the test while likelihood ratios were considered difficult to understand.

Additionally, multiple participants pointed out the need for flexible tables that allow for qualitative representation of the TA reviews. They explained that this is frequently needed when pooling is not possible either due to methodological challenges or differences among the index test(s) and reference standard in the studies included.

### Evidence tables heading

The aim of the header section was to give a brief description of the population, condition and the index and reference tests. The intent was to provide enough information about how the tests were applied in the studies, to allow users to judge to what extent the results are applicable in their own setting. This included the tests’ role, application, cut-off values, setting and the population included in the studies. Participants reported that presenting background information about the index and reference tests in the header of the table is helpful to contextualize the information before looking at the TA results. The majority preferred to place the number of participants and studies in the header as a method of avoiding repetition and saving space in the table if the information was the same for each row. It was noted that these evidence tables might be more difficult to use if there are different reference standard tests and multiple index tests or cut-off values.

### Evidence tables summarizing single TA systematic reviews

**Prevalence/pre-test probability/baseline risk estimates.** Presenting multiple prevalence estimates allows for interpretation of the test results in different populations as well as comparisons of the test performance in various populations and clinical settings. It may also help users decide which estimate is more applicable to their setting.

Presentation of this information went through multiple changes based on users’ feedback. Initially we removed presentation of three prevalence estimates to one/two prevalence estimates in columns (Appendix D in S1 Appendices show earlier tables with three prevalence estimates in rows). We subsequently added clinical scenarios to describe a typical patient in each prevalence group. Also, we changed the label of prevalence from percentage to natural numbers (per 1000) to be consistent with how results were presented in the reminder of the table.

When TA results were presented for 3 prevalence estimates in rows, respondents reported it was confusing and the information was overwhelming. At that time many preferred to have results of the test presented with only a single prevalence estimate. After changing to presenting different prevalence values in columns rather than in rows, more respondents preferred the table with two prevalence estimates compared with one prevalence estimate. Some noted it gave more information and demonstrated test performance in different settings but others felt it remained unclear what the different prevalence estimates represented. Hence, we added a clinical scenario to the label of the column to describe an average patient in each risk group (prevalence). Users viewed this positively and more respondents preferred showing the TA results based on two prevalence values in the table.

Users noted that the number of prevalence estimates that should be presented depends on the test and condition and the available evidence in the studies in the review, and may vary. However, it was noted that the prevalence estimates should be obtained from the highest quality evidence available that is applicable to the population of interest. For most tests presenting the results for two prevalence settings based on data from observational studies or from the included TA studies seems appropriate. All participants viewed clinical scenarios explaining an average patient in a pre-test probability group as helpful and thus this information should be provided whenever possible. The vast majority preferred clinical scenario to be placed inside the table as opposed to in the footnotes.

**Comments and explanation column.** The comments section of the table is intended to provide generic implications of the test results, e.g. “false positives may lead to unnecessary treatment or additional testing.” or specific information about the downstream consequences, whenever available. Comments were not seen as necessary and helpful to the interpretation of the values in the table for most users. Participants noted that, when authors are explaining the consequences of test results, comments should preferably be based on systematic review(s) of the literature to summarize the highest quality evidence available, and not based on assumptions. It was also suggested that the discussion of probable downstream implications be included in the body of the systematic review rather than in the summary table.

**Labeling of the effect estimate column.** Respondents noted that adding definitions to columns’ labels might be helpful for users not familiar with the definitions, or as a refresher. However, the labels increase the complexity of the table and some labels could be avoided such as definitions of sensitivity and specificity. The majority of respondents preferred ‘Number of results per 1000’ compared to ‘illustrative comparative numbers’ label as they explained it is easier to understand. Participants noted, although ‘per 1000’ label is repetitive and makes the table busier, it may be helpful to retain as a reminder for the reader and to avoid confusion with percentages.

**Rationale for quality of evidence (certainty or confidence in the effect estimates) rating.** We tested the best location to indicate the rational of quality of evidence in the table versus in footnotes. Some respondents noted that providing the rationale for quality of evidence rating in the footnotes is sufficient and may not need to be repeated in the table (with brief reason). While some respondents noted that a possible advantage of having the brief reason inside the table is drawing attention to the footnotes, others noted it could have an opposite effect and distract attention from the footnotes. Respondents generally agreed that footnotes explaining quality of evidence rating should be concise and briefly provide the reasons for the rating beyond just stating to which domain concerns applied (e.g. explain why there was concern about the risk of bias instead of just stating “risk of bias”). It was also noted that further elaboration about the quality of evidence rating can and should be provided in the text of the systematic review.

### Evidence tables summarizing comparative TA systematic reviews of 2 or more tests

These tables summarise the TA results of a comparison of two or more tests. Tests can be compared to each other and to the same reference standard directly in the same studies (direct comparison). Alternatively, one test can be compared to the reference standard in one set of studies and another test compared to the same reference standard in another set of studies to assess the same condition (indirect comparisons).

Most participants indicated that having data about two tests in one table is useful for comparison. However, this increased complexity of the tables. A single table that combines all information together with the comparison of the tests was viewed by majority of respondents as sufficient and more practical compared to presenting two tables for each test separately. Because there is a comparison between tests, users viewed the absolute differences between the values of the outcomes of each test as helpful information, but this made the table more complex. They also preferred to keep the number of studies, participants and the sensitivity and specificity values in the header. However, when tests were not directly compared in the same studies, participants did not always realize that the lower quality of evidence was due to indirect comparison.

### Evidence tables footnotes

The aim of the footnotes section was to give further details that are needed to explain judgements and the information provided in table cells. Many respondents did not read the footnotes. They noted that critical information that is needed to understand the results should be included directly in table cells and not be “hidden” in the footnotes, such as information about reference test or prevalence estimates, which were viewed as critical. Respondents preferred short, informative and easy to read statements. They noted that footnotes explaining the QoE ratings are helpful. It was also suggested that it is best to fit the footnotes on one page with the tables. There was general agreement that the labels “explanations” or “clarifications” would be preferred to the label “footnotes”.

## Discussion

In this article we present the research supporting the development of GRADE diagnostic evidence tables that display the results of TA systematic reviews. Overall, users found the results of TA reviews presented in a summary of findings table useful. However, they preferred simple tables that present the results in a format that is easy to apply to patients and populations. As a result of our sequential rounds of user testing and revisions, current versions of the TA systematic reviews evidence tables were agreed upon (Tables 1, 2 and 3: diagnostic evidence tables).

Our study has multiple strengths. We conducted extensive usability testing with different user groups with a variety of experience with GRADE, TA systematic reviews, and decision-making about tests. This helped us to develop table formats that will be useful for a diverse group of users as well as different applications and settings. We interviewed an international group of users whose first languages were often other than, but not limited to English, adding transferability of our findings and the usability of these tables around the world. We also contacted participants to clarify any confusion about their responses. Additionally, we asked users to suggest solutions to make the tables more useful and minimize any misunderstanding about the results. At least two investigators reviewed the notes and transcripts of the user testing interviews to minimize bias in describing our findings. Furthermore, this is the first empirical work to support the development and modification of GRADE diagnostic evidence tables. [9, 10]

Our study has limitations. Regardless of table layout, there is a learning curve associated with general understanding of the information about test accuracy and systematic reviews of studies measuring it. Understanding and using GRADE diagnostic evidence tables also requires getting accustomed to this presentation of information. The more often they are used, the more familiar users become with the tables. To avoid “learning effect” bias that may affect users feedback, we randomized the order by which participants saw the different formats. Additionally, we observed variability in comprehension and preference for the different formats. In some instances, preference was not in line with the correct understanding of the results, which created a challenge for the developers. When discordance between understanding and preference was observed, we relied on understanding as our primary outcome of interest to guide the development of different formats and to investigate the need to modify any parts of the table to make them more intuitive and transparent. Also, respondents were volunteers who agreed to participate in our study, which may be viewed as a limitation due to self-selection. To avoid that, we contacted a variety of potential respondents and included users that have a wide range of experience and background (Table 1).

When designing evidence tables for systematic reviews of test accuracy we considered the experience from developing GRADE evidence tables for interventions and plain language summaries [11–14]. However, this is the first study assessing users views about diagnostic evidence tables, which entail unique challenges. While determining patient important outcomes is relatively straightforward in intervention reviews, determining which outcomes to present in TA systematic reviews is more complicated. Since only TA results are available to authors of systematic reviews, we collected feedback about which form of TA results to present. We identified three formats that presented TA results in a layout that was acceptable to different users. Participants agreed that while sensitivity and specificity are the most commonly reported in TA systematic reviews, presenting pre- and post-test probability based on likelihood ratios and absolute TA results of TP, FP, TN and FN may be needed to comprehend the implications of the results by users. Based on our findings, we believe probabilities may be a more useful measure for decision making about the use of a test, albeit likely more difficult for some users to understand. We also believe table formats presenting individual TA values (i.e. TP, TN, FP, FN) may be more useful at the population level, while the format presenting likelihood ratios with pre- and post-test probabilities is more useful for decision making about individual patients in the clinical setting.

Presentation of multiple estimates of prevalence was identified by users as important for the application of the test and deciding whether to use it in a given setting. The decision to use the test may differ for a population with low prevalence versus higher prevalence as one considers the test results (e.g. TP, FN, TN, FP) and proportions for the specific population. However, unlike presentation of several baseline risk groups when estimating absolute effects of therapeutic interventions, the importance of presenting test accuracy results based on several assumed values of prevalence (pre-test probability) was not clear to participants until we added clinical scenarios describing an average patient in each prevalence (risk) group. How best to develop the clinical scenarios remains an open question. They could be obtained from the literature or suggested by an expert panel based on the observations from clinical practice. Participants also suggested that TA systematic review authors would need to ensure it is representative of an average patient in that risk category, a challenge that requires clinical expertise. For specific conditions authors of systematic reviews may be able to use validated prediction models of baseline risk or pre-test probability to support their scenarios (e.g. Framingham coronary heart disease prediction score). However, it is likely that identifying studies with an applicable example clinical scenario may be challenging for many topics.

Systematic reviews of interventions typically address questions comparing the outcomes from two management options, even if one of them is no intervention or placebo. This, however, changes with the more widespread use of network meta-analytical approaches that enable multiple direct and indirect comparisons. Systematic reviews of TA assess the accuracy of a single test or multiple tests. This creates a unique challenge, conceptually similar to the challenges of simple presentation of the results of multiple comparisons of treatments, as one has to develop tables that can present results of the accuracy estimates either for single test or multiple tests while attempting to keep the structure similar to minimize confusion among users. There was general consensus among our study participants that tables should be simple both in the information they provide and in their design. Achieving this was naturally more challenging when developing evidence tables for comparative diagnostic accuracy of more than one test.

The majority of the users agreed that evidence tables should be included in TA systematic reviews, as it improves accessibility of the results. However, similar to intervention reviews, we also learned that authors may have difficulty preparing the tables since not all tests or conditions will fit a standard layout. There may also be other methodological issues such as ability to pool the data, especially from different types of studies (e.g. differences in reference test, index tests, indirect comparisons, heterogeneity, etc.). Participants reported that evidence tables would be more acceptable to authors of TA reviews if there were some options for content and layout to accommodate different clinical topics (e.g. interactive table). This led to the development of an electronic version of a summary table that integrates narrative interpretation of the results as well as definitions without crowding the table and making it overwhelming. This interactive format is currently under development and testing.

## Conclusions

In this article we presented the results of extensive user testing that led to the development of the current formats of the evidence tables for test accuracy as suggested by the GRADE approach. These formats can be produced using GRADEpro Guideline Development Tool (www.guidelinedevelopment.org or www.gradepro.org) and are being further developed into electronic interactive tables that will suit the needs of different users. These tables and their impact on understanding the results and making decisions in regards to the test(s), will be evaluated through randomized trials.

## Supporting Information

S1 Appendices Appendix A, Workshop package grading the quality of evidence and preparing summary of findings tables about diagnostic tests. Appendix B, Interview packages. Appendix C, Enhancing the usability and usefulness of summary of findings tables and evidence profiles for decision making about diagnostic tests. Appendix D, Earlier tables with 3 prevalence estimates in rows.
(DOCX)

## References

[1] Guyatt GH, Oxman AD, Santesso N, Helfand M, Vist G, Kunz R, et al GRADE guidelines: 12. Preparing summary of findings tables-binary outcomes. J Clin Epidemiol. 2013;66(2):158–72. Epub 2012/05/23. 10.1016/j.jclinepi.2012.01.012 . 22609141

[2] Guyatt GH, Thorlund K, Oxman AD, Walter SD, Patrick D, Furukawa TA, et al GRADE guidelines: 13. Preparing summary of findings tables and evidence profiles-continuous outcomes. J Clin Epidemiol. 2013;66(2):173–83. Epub 2012/11/03. 10.1016/j.jclinepi.2012.08.001 . 23116689

[3] Grade Working Group. Available: http://www.gradeworkinggroup.org. Accessed: 1 July 2014.

[4] Schunemann HJ, Oxman AD, Brozek J, Glasziou P, Bossuyt P, Chang S, et al GRADE: assessing the quality of evidence for diagnostic recommendations. BMJ. 2008;13(6). 10.1136/ebm.13.6.162-a 19043023

[5] Bossuyt PM, Irwig L, Craig J, Glasziou P. Comparative accuracy: assessing new tests against existing diagnostic pathways. BMJ. 2006;332(7549):1089–92. 10.1136/bmj.332.7549.1089 16675820 PMC1458557

[6] Mustafa RAW, Cheung A, Prediger B, Brozek J, Bossuyt P, Garg AX, et al Decision-making about healthcare related tests and diagnostic strategies: A review of methodological and practical challenges and introduction to a new series Journal of clinical epidemiology. 10.1016/j.jclinepi.2017.09.003 28916488

[7] Tiselius HG, Alken P, Buck C, Gallucci M, Knoll T, Sarica K, et al Guidelines on urolithiasis. Arnhem, Netherlands: European Association of Urology; 2012.

[8] Hsieh HF, Shannon SE. Three approaches to qualitative content analysis. Qualitative health research. 2005;15(9):1277–88. 10.1177/1049732305276687 . 16204405

[9] World Health Organization. Automated Real-time Nucleic Acid Amplification Technology for Rapid and Simultaneous Detection of Tuberculosis and Rifampicin Resistance: Xpert MTB/RIF System—Policy Statement. Geneva, Switzerland: World Health Organization; 2011. 26158191

[10] World Health Organization. Automated Real-Time Nucleic Acid Amplification Technology for Rapid and Simultaneous Detection of Tuberculosis and Rifampicin Resistance: Xpert MTB/RIF Assay for the Diagnosis of Pulmonary and Extrapulmonary TB in Adults and Children: Policy Update. WHO Guidelines Approved by the Guidelines Review Committee Geneva 2013. 25473701

[11] Rosenbaum SE, Glenton C, Nylund HK, Oxman AD. User testing and stakeholder feedback contributed to the development of understandable and useful Summary of Findings tables for Cochrane reviews. J Clin Epidemiol. 2010;63(6):607–19. 10.1016/j.jclinepi.2009.12.013 . 20434023

[12] Rosenbaum SE, Glenton C, Oxman AD. Summary-of-findings tables in Cochrane reviews improved understanding and rapid retrieval of key information. J Clin Epidemiol. 2010;63(6):620–6. 10.1016/j.jclinepi.2009.12.014 . 20434024

[13] Glenton C, Santesso N, Rosenbaum S, Nilsen ES, Rader T, Ciapponi A, et al Presenting the results of Cochrane Systematic Reviews to a consumer audience: a qualitative study. Med Decis Making. 2010;30(5):566–77. 10.1177/0272989X10375853 . 20643912

[14] Vandvik PO, Santesso N, Akl EA, You J, Mulla S, Spencer FA, et al Formatting modifications in GRADE evidence profiles improved guideline panelists comprehension and accessibility to information. A randomized trial. J Clin Epidemiol. 2012;65(7):748–55. Epub 2012/05/09. 10.1016/j.jclinepi.2011.11.013 . 22564503

