# Supplementary material for: Decision-Making about Healthcare Related Tests and Diagnostic Strategies: User Testing of GRADE Evidence Tables
Source: PLoS One. 2015 Oct 16;10(10):e0134553. doi: 10.1371/journal.pone.0134553 (PMC4608675; doi:10.1371/journal.pone.0134553)
Supplement: S1 Appendices — Appendix A, Workshop package grading the quality of evidence and preparing summary of findings tables about diagnostic tests. Appendix B, Interview packages. Appendix C, Enhancing the usability and usefulness of summary of findings tables and evidence profiles for decision making about diagnostic tests. Appendix D, Earlier tables with 3 prevalence estimates in rows. (DOCX) [file pone.0134553.s001.docx]

Supplement 1

Appendix A: workshop package grading the quality of evidence and preparing summary of findings tables about diagnostic tests

**Aim of this exercise**Summary of Findings (SoF) tables provide a concise, easy to understand summary of results of a systematic review. The GRADE Working Group and the Cochrane Collaboration Applicability and Recommendations Methods Group have suggested an approach to grading the quality of evidence for questions about diagnostic accuracy. This workshop will introduce the approach based on examples. We will discuss the content of the currently suggested format of SoF tables and seek feedback on improving them.

1. Read the ***abstract*** of the review
2. Identify the ***clinical question*** asked and its components

**Review:** Galactomannan detection for invasive aspergillosis in immunocompromized patients. Leeflang MM et al. Cochrane Database of Systematic Reviews 2008, Issue 4. Art. No.: CD007394.

**Question:** ___________________________________________________________________________

____________________________________________________________________________________

**Setting:** _____________________________________________________________________________

**Population (prevalence):** _______________________________________________________________

**New test:** ____________________________________________________________________________

**Existing test:** _________________________________________________________________________

**Cut-off criterion:** ______________________________________________________________________

***Exercise***

Please review attached examples of proposed Summary of Findings table and an Evidence Profile.

**The aim of the following questions is to assess whether the currently proposed table format is easy to understand – we do not evaluate your knowledge.**

**Do you have experience with diagnostic test accuracy studies?**  Yes   No

**Do you have experience with systematic reviews of diagnostic test accuracy studies?**  Yes  No

***Overall appearance of the tables***

**To what extent do you agree with the following statements?**

1. **The information in the Evidence Profile is clearly presented.**

| I strongly  disagree | I disagree | I somewhat  disagree | I am not sure | I somewhat  agree | I agree | I strongly agree |
| --- | --- | --- | --- | --- | --- | --- |

1. **The information in the Summary of Findings table is clearly presented.**

| I strongly  disagree | I disagree | I somewhat  disagree | I am not sure | I somewhat  agree | I agree | I strongly agree |
| --- | --- | --- | --- | --- | --- | --- |

1. **Generally, the Evidence Profile is easy to understand.**

| I strongly  disagree | I disagree | I somewhat  disagree | I am not sure | I somewhat  agree | I agree | I strongly agree |
| --- | --- | --- | --- | --- | --- | --- |

1. **Generally, the Summary of Findings table is easy to understand.**

| I strongly  disagree | I disagree | I somewhat  disagree | I am not sure | I somewhat  agree | I agree | I strongly agree |
| --- | --- | --- | --- | --- | --- | --- |

Please provide suggestions for improvement, in particular if you disagree with the above statements:

***Comprehensibility***

1. **In the row “true positives” under the heading “prevalence 1%” the number “6 (5 to 8)” means that:**

6 out of 100 patients who receive the new test (95% confidence interval: 5 to 8) are correctly identified as having the disease.

6 out of 1000 patients who receive the new test (95% confidence interval: 5 to 8) are correctly identified as having the disease.

6 out of 2777 patients who receive the new test (95% confidence interval: 5 to 8) are correctly identified as having the disease.

only 6 out of 1000 patients who suffer the disease (95% confidence interval: 5 to 8) will test positive.

if the prevalence decreases from 12% to 1%, 6 additional people with the disease will be identified.

I cannot find the answer

I do not understand the question

1. **The risk of false positive results in patients who receive a new test in a setting with a prevalence of 1% is:**

6 per 1000  28 per 1000  30 per 1000  50 per 1000  89 per 1000

I cannot find the answer

I do not understand the question

1. **“High quality of evidence” for the outcome “true positives” refers to:
   (Check the most complete answer)**

the extent to which we can be confident that the estimate of the rate of “true positives” is correct

the extent to which we can be confident about the consequences important to patients correctly identified as having the disease

an overall estimate of the quality of all reviewed diagnostic and therapeutic studies

the overall quality of evidence of the reviewed diagnostic studies and the confidence about the consequences of being classified as “true positive” for patients in whom the test will be used

the risk of bias of reviewed diagnostic studies.

I cannot find the answer

I do not understand the question

1. **The number 7 in the “importance” column for “true positive” results in the Evidence Profile indicates:
   (Check the most complete answer)**

low importance of “true positive results, implying they are not critical to making a decision

that the correct labeling of patients as having the disease in question is sufficient for recommending the test in clinical practice.

that the patient-important consequences related with being correctly diagnosed as having the disease are regarded as very important

that “true positives” are the only outcome that should be considered for decision-making

that the clinical decision about using the test in question in immunocompromised patients should not be based on the true positive results

I cannot find the answer

I do not understand the question

1. **In the framework or grading the quality of evidence, directness refers to:
   (Choose ALL answers that you think are correct)**

the degree to which we are confident how the outcomes important to patients will be affected by the correct or incorrect diagnosis and subsequent management

the degree to which the tests studied are comparable to those being used in clinical practice

the degree to which the diagnostic expertise of people applying the tests in the studies is comparable to the expertise of those who will interpret the test in clinical practice

the degree to which patients in the studies resemble those in whom the test will be used in clinical practice

whether the tests being assessed and the reference standard were all assessed in the same study populations or whether each of the tests was compared to the reference standard in a different study population

I cannot find the answer

I do not understand the question

1. **Please provide any comments that you may have about the comprehensiveness of the tables, including suggestions about how it could be improved, e.g. should there be a graphical presentation of the results?**

|  |
| --- |

***Accessibility of the results***

**To what extent do you agree with the following statements?**

1. **The authors of a systematic review have indicated what they considered to be the most important outcomes for someone considering the diagnostic test.**

| I strongly  disagree | I disagree | I somewhat  disagree | I am not sure | I somewhat  agree | I agree | I strongly agree |
| --- | --- | --- | --- | --- | --- | --- |

1. **What outcomes were the most important according to the authors?**

True positive  True negative  False positive  False negative  Inconclusive

Resource use  I cannot find the answer  I do not understand the question

1. **It was easy to find the information about what outcomes are the most important.**

| I strongly  disagree | I disagree | I somewhat  disagree | I am not sure | I somewhat  agree | I agree | I strongly agree |
| --- | --- | --- | --- | --- | --- | --- |

1. **It was easy to find the results for each of the outcomes.**

| I strongly  disagree | I disagree | I somewhat  disagree | I am not sure | I somewhat  agree | I agree | I strongly agree |
| --- | --- | --- | --- | --- | --- | --- |

1. **It was easy to understand the results for each of the outcomes.**

| I strongly  disagree | I disagree | I somewhat  disagree | I am not sure | I somewhat  agree | I agree | I strongly agree |
| --- | --- | --- | --- | --- | --- | --- |

1. **The main findings of the review are presented in a way that would help me to make a decision.**

| I strongly  disagree | I disagree | I somewhat  disagree | I am not sure | I somewhat  agree | I agree | I strongly agree |
| --- | --- | --- | --- | --- | --- | --- |

1. **It was easy to find information about the quality of the evidence for each outcome.**

| I strongly  disagree | I disagree | I somewhat  disagree | I am not sure | I somewhat  agree | I agree | I strongly agree |
| --- | --- | --- | --- | --- | --- | --- |

1. **Overall, how would you rate the accessibility of the main findings of this review?**By “accessibility” we mean the extent to which the main findings are easy to find, understand, and use by someone making a decision.

| Very inaccessible | Inaccessible | Somewhat  inaccessible | I am not sure | Somewhat  accessible | Accessible | Very accessible |
| --- | --- | --- | --- | --- | --- | --- |

1. **Please provide comments you may have about the accessibility of the main findings of the review in the space below, including suggestions about how this could be improved:**

|  |
| --- |

1. **Is there any other information you would like to see in the tables summarizing the results of a review?**

Yes  No  I do not know

**If yes, please describe the information you would like to see presented additionally.**

***Evaluation of this QUESTIONNAIRE***

1. **Do you have any suggestions how to improve the comprehension and clarity of the above questions?**

Yes  No  I do not know

**If yes, please list the questions that were challenging and provide suggestions for improvement.**

|  |
| --- |

**Workshop date:**

**Venue:**

**Facilitators:**

Exercise

- *Work in small groups*
- *Select someone from your small group to report back to the whole group (take notes!)*
- *Watch the time*
- *Follow the subsequent instructions*

Instructions (outline – you will find detailed instructions on the respective pages as labeled)

1. Read the ***abstract*** of the systematic review (attachment)
2. Identify the ***clinical question*** asked in the review and its components (page 2)
3. Identify the ***main comparison*** from the review that you want to work on (e.g. BNP vs no BNP). Read the relevant sections of the review focusing on the text marked-up on the margin.
4. Complete the assessment of the quality of evidence in an evidence profile (page 3, instructions on page 4 and 5)

- Go to the marked-up text in the systematic review to evaluate the quality of evidence
- Make judgement about the quality of the evidence for each outcome
- Make judgments about the overall quality of evidence

1. Provide estimates of the effect of using a test in a population of patients with an assumed prevalence. Obtain the sensitivity and the specificity of the test in order to calculate the true positives (TP), true negatives (TN), false positives (FP) and false negatives (FN) results (page 6)

- Use a hypothetical pre-test probability (prevalence) of the disease of 60%.
- Calculate true positives, false positives, true negatives, and false negatives (page 6)

1. Provide information about the magnitude of desirable and undesirable outcomes (page 7 and 8)

- Choose the most important outcome for decision making
- Rate the relative importance of the effect
- Fill out the table to provide information about the outcomes

1. Develop a recommendation for the use of this test (page 11 and 12)

*During the exercise please contemplate how a Summary of Findings table for systematic reviews of diagnostic accuracy studies and an Evidence Profile for decision making (choosing among available tests) should look like.*

**1.** Specify details of the review and the clinical question asked

***Title of the review****: Diagnostic accuracy of pleural fluid NT-pro-BNP for pleural effusions of cardiac origin: a systematic review and meta-analysis*

**Identify the following components:**

| **Patients** (P: Population) and **Prevalence:** | |
| --- | --- |
| **Purpose** of the test (triage, replacement or add-on, screening, making diagnosis, monitoring) | |
| **New/index test** (I: Intervention): | |
| **Existing/comparator/reference test** (C: Comparator): | |
| **Outcomes of interest** (O): [consider all outcomes that patients could experience with or without treatment – use worksheet 1 on page 3] | |
| **Cut-off threshold** (if relevant): | |
|  |  |
|  |  |

**2.** Assess ***quality of evidence*** across studies for ***each aspect*** in a review
 *(see example at the end of this work package)*

**Quality assessment for diagnostic accuracy studies**

| Test result | No. of studies | Study  design | Factors that may decrease quality of evidence | | | | | | **Final**  **Quality** (symbols) |
| --- | --- | --- | --- | --- | --- | --- | --- | --- | --- |
|  |  |  | Risk of bias | Publication bias | Inconsistency | Imprecision | Indirectness of test accuracy | Other factors |  |
| sensitivity  (TP + FN) |  |  |  |  |  |  |  |  |  |
| specificity  (FP + TN) |  |  |  |  |  |  |  |  |  |
|  |  |  |  |  |  |  |  |  |  |
| Inconclusive |  |  |  |  |  |  |  |  |  |
| Cost |  |  |  |  |  |  |  |  |  |
| Footnotes: | | | | | | | | | |

**GRADE quality assessment criteria for diagnostic accuracy studies**

| **Underlying study design** |
| --- |
| Valid diagnostic accuracy studies (cross-sectional or cohort) in patients with diagnostic uncertainty and direct comparison of test results with an appropriate reference standard are initially rated as **high quality** evidence. These studies are rare, however. |
| **Factors that may decrease the quality of evidence** |
| - Limitations in design or execution of the study (risk of bias) - Indirectness (comparison or the population, new test, comparison test, and outcomes) - Inconsistency in study results - Imprecise results - High probability of publication bias   If any of the factors warranting downgrading is present, consider if the limitations are ***serious*** (downgrade by ***one*** level) or ***very serious*** (downgrade by ***two*** levels). |
| **Factors that may increase the quality of evidence** |
|  |

**Factors that determine or decrease the quality of evidence for studies of diagnostic accuracy**

| **Factors that determine quality** | **Explanations how they differ from evidence about treatment options** |
| --- | --- |
| **Study design** | ***Different criteria*** for accuracy studies than for management trials  Cross-sectional or cohort studies in patients with diagnostic uncertainty and direct comparison of test results with an appropriate reference standard are considered high quality and can move to moderate, low or very low depending on the following factors. |
| **Limitations in design or execution** (risk of bias) | ***Different criteria*** for accuracy studies than for management trials  Consecutive patients should be recruited as a single cohort and not classified by disease state and the selection as well as the referral process should be clearly described. Tests should be performed in all patients in the same patient population for the new test and a well described reference standard – the evaluators should be blind to the results of the alternative test and reference standard. |
| **Indirectness** of evidence |  |
| *Outcomes* | ***Similar criteria*** for accuracy studies and for management trials  Diagnostic accuracy studies do not provide direct evidence about patient-important outcomes. One must make deductions about the balance between the presumed influences of any differences in true and false positives and true and false negatives on patient-important outcomes in relationship to test complications and costs (diagnostic accuracy studies typically provide low quality evidence for making recommendations due to indirectness of outcomes, similar to surrogate outcomes for treatments). |
| *Patient populations, diagnostic test/intervention, comparison test/intervention, and indirect comparisons* | ***Similar criteria*** for accuracy studies and for management trials  The quality of evidence can be lowered if there are important differences:   1. between the populations studied and those for whom the recommendation is intended (*e.g.* the spectrum of disease or co-morbidity) 2. in tests studied and the expertise of those applying them in the studies compared to the settings for which the recommendations are intended   The quality of evidence can be also lowered if the tests being compared are each compared to a reference (gold) standard in different studies but not directly in the same studies. |
| **Inconsistency** of the results | ***Similar criteria*** for accuracy studies and for management trials  For accuracy studies, unexplained inconsistency in sensitivity, specificity or likelihood ratios (rather than relative risk or mean differences) can lower the quality of evidence. |
| **Imprecision** of the results | ***Similar criteria*** for accuracy studies and for management trials  For accuracy studies wide confidence intervals for estimates of test accuracy, or true and false positive and negative rates can lower the quality of evidence. |
| **Publication bias** | ***Similar criteria*** for accuracy studies and for management trials  A high risk of publication bias (e.g. evidence from small studies for a new intervention or test, or asymmetry in a funnel plot) can lower the quality of evidence. |

References: 1) Guyatt GH et al. What is "quality of evidence" and why is it important to clinicians? BMJ 2008;336:995–8. 2) Schünemann HJ et al. Grading quality of evidence and strength of recommendations for diagnostic tests and strategies. BMJ 2008;336:1106–10.

**Quality criteria of diagnostic accuracy studies**

Quality criteria of diagnostic accuracy studies derived from QUADAS (Reitsma JB, Rutjes AWS, Whiting P, Vlassov VV, Leeflang MMG, Deeks JJ,. Chapter 9: Assessing methodological quality. In: Deeks JJ, Bossuyt PM, Gatsonis C (editors), Cochrane Handbook for Systematic Reviews of Diagnostic Test Accuracy Version 1.0.0. The Cochrane Collaboration, 2009. Available from: http://srdta.cochrane.org/

| **#** | **Item** | **Yes** | **Unclear** | **No** |
| --- | --- | --- | --- | --- |
| **1** | Was the spectrum of patients representative of the patients who will receive the test in practice? (representative spectrum) | 🞎 | 🞎 | 🞎 |
| **2** | Is the reference standard likely to classify the target condition correctly? (acceptable reference standard) | 🞎 | 🞎 | 🞎 |
| **3** | Is the time period between reference standard and index test short enough to be reasonably sure that the target condition did not change between the two tests? (acceptable delay between tests) | 🞎 | 🞎 | 🞎 |
| **4** | Did the whole sample or a random selection of the sample, receive verification using the intended reference standard? (partial verification avoided) | 🞎 | 🞎 | 🞎 |
| **5** | Did patients receive the same reference standard irrespective of the index test result? (differential verification avoided) | 🞎 | 🞎 | 🞎 |
| **6** | Was the reference standard independent of the index test (i.e. the index test did not form part of the reference standard)? (incorporation avoided) | 🞎 | 🞎 | 🞎 |
| **7** | Were the reference standard results interpreted without knowledge of the results of the index test? (index test results blinded) | 🞎 | 🞎 | 🞎 |
| **8** | Were the index test results interpreted without knowledge of the results of the reference standard? (reference standard results blinded) | 🞎 | 🞎 | 🞎 |
| **9** | Were the same clinical data available when test results were interpreted as would be available when the test is used in practice? (relevant clinical information) | 🞎 | 🞎 | 🞎 |
| **10** | Were uninterpretable/ intermediate test results reported? (uninterpretable results reported) | 🞎 | 🞎 | 🞎 |
| **11** | Were withdrawals from the study explained? (withdrawals explained) | 🞎 | 🞎 | 🞎 |

**3.** Provide estimates of the effect of using a test in a population of patients with an assumed prevalence.

Assumed typical pre-test probability (“prevalence”): 60 %

| **Test findings** | |  |
| --- | --- | --- |
| Pooled sensitivity | _____ (95% CI: _____ to _____) |  |
| Pooled specificity | _____ (95% CI: _____ to _____) |  |
| **Consequences** | | **Quality of Evidence** |
|  | **Number per 1000 tested*** |  |
| **TP** |  |  |
| **TN** |  |  |
| **FP** |  |  |
| **FN** |  |  |
| **Inconclusive results**** |  |  |
| **Cost** |  |  |

* all results are given per 1000 patients tested based on the prevalence of ____ % and pooled sensitivity and specificity.

** inconclusive results are either uninterpretable, indeterminate or intermediate test results

Calculate an absolute number of patients with a given test result based on the combined sensitivity and specificity from meta-analysis and an assumed prevalence of a target condition using a 2x2 table.

|  |  | **Reference standard** | |  |
| --- | --- | --- | --- | --- |
|  |  | *Disease present* | *Disease absent* |  |
| **New Test** | *Positive* | TP | FP |  |
|  |  | **_____** | **_____** |  |
|  | *Negative* | FN | TN |  |
|  |  | **_____** | **_____** |  |
| Prevalence: **_____ %** | | **_____** | **_____** | **1000** |

Example calculation for an ***assumed prevalence of 20%***.

|  |  | **Reference standard** | |  |
| --- | --- | --- | --- | --- |
|  |  | *Disease present* | *Disease absent* |  |
| **New Test** | *Positive* | TP = sensitivity x **200** | FP = (1 – specificity) x **800** |  |
|  |  |  |  |  |
|  | *Negative* | FN = (1 – sensitivity) x **200** | TN = specificity x **800** |  |
|  |  |  |  |  |
| Prevalence: **20 %** | | **200** | **800** | **1000** |

**4.** Choose the most important outcomes for decision making

Choose the most important outcomes for decision making that are a consequence of the condition, disease or a problem (e.g. mortality, stroke, disability, bleeding complications, etc.). Consider all consequences regardless of whether or not the correct diagnosis is made (e.g. false positives and whether the consequences happen with or without subsequent therapy (e.g. false negatives will not receive potentially beneficial treatment and false positives may receive unnecessary but potentially harmful therapy). If you are not familiar with the topic talk to your neighbor or make assumptions and the best guesses.

Consider the outcomes that:

- are part of the natural history of a disease
- may have been reported in systematic reviews and/or individual studies
- Might be important to someone making a decision to use or not to use a test, including complications of performing tests being compared (include both benefits and adverse effects, and costs if relevant)

Consider efficacy of a treatment for all important outcomes and the rate of adverse effects.

Complete a list of the assumptions about patient outcomes/consequences (use worksheet below):

- E.g. Relative Risk Reduction for Mortality: 50%
- E.g. Relative Risk Increase for severe adverse outcomes: 20%

You may draw a flow diagram of what may happen to patients to support your thinking.

List all outcomes and specify how frequently they would occur in each of the four categories (overwrite the grey example):

| **Outcome** | **Assumptions about the effect of appropriate treatment**  (RRR) | **Include in TP, TN, FP, FN**  (at what rate would the outcome occur with or without correct therapy?) | | | |
| --- | --- | --- | --- | --- | --- |
|  |  | **TP** | **TN** | **FP** | **FN** |
| 1. mortality at 30 days | 50% | 35%^1^ | 5%^2^ | 7%^3^ | 70%^4^ |
|  |  |  |  |  |  |
|  |  |  |  |  |  |
|  |  |  |  |  |  |
|  |  |  |  |  |  |
|  |  |  |  |  |  |
|  |  |  |  |  |  |

**Footnotes:**

^1^ Those correctly diagnosed with the disease and treated successfully (RRR: 50%)

^2^ This proportion will die regardless within 30 days

^3^ Adverse effects of therapy cause an additional 2% mortality

^4^ Proportion that will die because they will have not received therapy or therapy will have been delayed

**5.** Rate the relative importance of the effect

Consider ***patient-important consequences*** of being correctly or incorrectly classified as having or not having a disease. Assess the ***relative importance*** of each outcome and decide which is critical for the decision to use the test. Use the percentages that you have assumed in worksheet 1. Begin by listing the consequences followed by the percentage and then rate the outcomes according to the following scale.

| Rate the importance on a 9-point scale (please note that you can assign the same rating several times): | |
| --- | --- |
| **1 – 3** | **not important** (not included in the *evidence profile*) |
| **4 – 6** | **important, but not critical** for making a decision (included in the *evidence profile*) |
| **7 – 9** | **critical** for making a decision (included in the *evidence profile*) |

| **Patient-important consequences** | **Importance** |
| --- | --- |
| TP: (*e.g.* mortality 35%; severe stroke 5%; etc) |  |
| TN: |  |
| FP: |  |
| FN: |  |
| Inconclusive results*: |  |
| Cost: |  |

* uninterpretable, indeterminate or intermediate test results

**6.** Consider the patient important outcomes and how directly they relate to diagnostic accuracy

**Summary of Findings Table based on patient outcomes**

**Population/ Setting:**

**New Test/ cut-off value: Comparison Test/ cut-off value**: **Reference Test:**

**Sensitivity ___ (CI___-___) Specificity ___ (CI ___-___)**

| **Patient outcomes**  **(importance)** | **Assumed patient consequences** | **Basis of assumptions** | **Results per 1000 patients/year**  **for a given pre-test probability** | | | **Quality of Evidence about patient outcomes** |
| --- | --- | --- | --- | --- | --- | --- |
|  |  |  | **__%** | **__%** | **__%** |  |
| Mortality (9) | True positive will have improved mortality due to early diagnosis and false negative will have worse mortality due to late diagnosis | We assumed 10% increased mortality risk every year from CHF to be uniformed across different stages. We also assumed 10% RRR in mortality for early diagnosis of CHF. | 0 more  patients will live | 1 more  patients will live | 1 more  patient will live | ⊕OOO  Very low |
|  |  |  |  |  |  |  |
|  |  |  |  |  |  |  |
|  |  |  |  |  |  |  |
|  |  |  |  |  |  |  |
|  |  |  |  |  |  |  |
|  |  |  |  |  |  |  |

**Footnotes: ****

(1)

**7.** Move from evidence to recommendation

**6.1. Determine overall quality of evidence**

| \| **Choose one** \| **Symbol** \| **Quality** \| **Interpretation** \| \| --- \| --- \| --- \| --- \| \| 🞏 \| ⊕⊕⊕⊕ \| **High** \| Further research is very unlikely to change our confidence in the estimate of effect or accuracy. \| \| 🞏 \| ⊕⊕⊕ \| **Moderate** \| Further research is likely to have an important impact on our confidence in the estimate of effect or accuracy and may change the estimate. \| \| 🞏 \| ⊕⊕O \| **Low** \| Further research is very likely to have an important impact on our confidence in the estimate of effect or accuracy and is likely to change the estimate. \| \| 🞏 \| ⊕ \| **Very low** \| Any estimate of effect or accuracy is very uncertain. \|   Overall quality of evidence across all *critical* outcomes is: |
| --- | --- | --- | --- | --- | --- | --- | --- | --- | --- | --- | --- | --- | --- | --- | --- | --- | --- | --- | --- | --- |

**6.2. Values and preferences (assume a set of values for each outcome considered)**

*Example: A high value may be placed on the true positives, false negatives, and false positives because the associated treatments both have important benefits and harms, but relatively low values on true negatives and resources use.*

| **Outcome** | **Values and preferences** |
| --- | --- |
| **TP** |  |
| **TN** |  |
| **FP** |  |
| **FN** |  |
| **Inconclusive results** |  |
| **Cost** |  |

**6.3. Draft recommendation**

|  |
| --- |

**8.** Decide about the strength of a recommendation (strong or conditional/weak)

Use the table below to make a judgment. The four factors in this table will determine whether the recommendation is likely to be “strong” or “conditional” (aka “weak”). Frequent positive answers increase the likelihood of a strong recommendation. Make sure to add an explanation for your judgment.

| **Factors that can weaken the strength of a recommendation** | **Decision** | **Explanation** |
| --- | --- | --- |
| **High or moderate quality evidence** *(is there high or moderate quality evidence?)*  The higher the quality of evidence, the more likely is a strong recommendation. The lower the quality of evidence the more likely is a conditional/weak recommendation | 🞏 Yes  🞏 No |  |
| **Certainty about the balance of benefits versus harms and burdens** *(is there certainty?)*  The larger the difference between the desirable and undesirable consequences and the certainty around that difference, the more likely a strong recommendation. The smaller the net benefit and the lower the certainty for that benefit, the more likely is a conditional/weak recommendation. | 🞏 Yes  🞏 No |  |
| **Certainty or similarity in values** *(is there certainty?)*  The smaller the variability or the greater the certainty around values and preferences, the more likely is a strong recommendation. | 🞏 Yes  🞏 No |  |
| **Resource implications (are the resources consumed worth the expected benefit)**  The higher the costs of an intervention compared to the alternative that is considered and other cost related to the decision – that is, the more resources consumed – the more likely is a conditional/weak recommendation. | 🞏 Yes  🞏 No |  |

If consensus has not been reached by discussion, the panel can use the following table to record their views (votes) about the strength of the recommendation related to a specific management option, based on their analysis of the available evidence and its quality, the benefits and downsides, values and preferences, and resource use (cost). This assessment is then mapped to the strength of recommendation for the use, or non-use, of each intervention.

| **If you need to vote: Insert the number of votes for the recommendation in each category** | | | | |
| --- | --- | --- | --- | --- |
| **Assessors’ view of the balance between desirable and undesirable consequences of the intervention** | Desirable consequences clearly outweigh undesirable consequences | Desirable consequences probably outweigh undesirable consequences | Undesirable consequences probably outweigh desirable consequences | Undesirable consequences clearly outweigh desirable consequences |
| **Strength of recommendation** | Strong for an intervention | Conditional (weak) for an intervention | Conditional (weak) against an intervention | Strong against an intervention |
| **Wording of a recommendation** | We recommend to “do something” | We suggest (conditionally recommend) to “do something” | We suggest (conditionally recommend) not to “do something” | We recommend not to “do something” |
| **Number of votes** |  |  |  |  |

| **Strength of the recommendation:** | 🞏 Strong  🞏 Conditional (weak) |
| --- | --- |

| **Final recommendation** | |
| --- | --- |
|  | |
| Strength: | Quality of evidence: |
| **Assumptions about underlying values and preferences** | |
|  | |
| **Remarks** | |
|  | |

**Alternative Example**

Hamon M et al. Diagnostic performance of multislice spiral computed tomography of coronary arteries as compared with conventional invasive coronary angiography: a meta-analysis. J Am Coll Cardiol 2006;48:1896–1910.

**Question:** Should multislice spiral computed tomography rather than conventional coronary angiography be used to diagnose coronary artery disease (CAD)?

**Patient or population:** Adults suspected of coronary artery disease

**Settings:** The included trials were conducted in Europe and North America

**Quality assessment for diagnostic accuracy studies – example**

| Outcome | No. of studies | Study  design | Factors that may decrease quality of evidence | | | | | **Final**  **quality** |
| --- | --- | --- | --- | --- | --- | --- | --- | --- |
|  |  |  | Risk of bias | Indirectness | Inconsistency | Imprecision | Publication bias |  |
| **True positives**  (patients with CAD) | 21 studies (1570 pts) | cross-sectional^1^ | No serious limitations | Little or no uncertainty | Serious inconsistency^3^ | No serious imprecision | Not detected^4^ | ⊕⊕⊕  moderate |
| **True negatives** (patients without CAD) | 21 studies (1570 pts) | cross-sectional^1^ | No serious limitations | Little or no uncertainty | Serious inconsistency^3^ | No serious imprecision | Not detected^4^ | ⊕⊕⊕  moderate |
| **False positives** (patients incorrectly classified as having CAD) | 21 studies (1570 pts) | cross-sectional^1^ | No serious limitations | Little or no uncertainty | Serious inconsistency^3^ | No serious imprecision | Not detected^4^ | ⊕⊕⊕  moderate |
| **False negatives** (patients incorrectly classified as not having CAD) | 21 studies (1570 pts) | cross-sectional^1^ | No serious limitations | Some uncertainty^2^ | Serious inconsistency^3^ | No serious imprecision | Not detected^4^ | ⊕⊕  low |
| Inconclusive | – | – | – | – | – | – | – | – |
| Cost | – | – | – | – | – | – | – | – |
| ^1^ All patients were selected to have conventional coronary angiography and were, therefore, generally presenting with high probability of coronary artery disease (median prevalence in included studies 63.5%, range 6.6-100%)  ^2^ Some uncertainty about directness for false negatives related to detrimental effects from delayed diagnosis or myocardial insult, reducing quality of evidence for consequences of false negative test results from high to moderate.  ^3^ Statistically significant, unexplained heterogeneity of results for sensitivity (proportion of patients with positive coronary angiography with positive computed tomography scan), specificity (proportion of patients with negative coronary angiography with negative computed tomography scan), likelihood ratios, and diagnostic odds ratios, reducing quality of evidence for consequences of true positive, true negative, and false positive results from high to moderate and of false negative results from moderate to low.^13^  ^4^ Possibility of publication bias not excluded but not considered sufficient to downgrade quality of evidence. | | | | | | | | |

**Summary of findings – example.** Assumed pre-test probability (prevalence) was 20%.

| **Test findings** | | | |
| --- | --- | --- | --- |
| Pooled sensitivity | 0.96 (95% CI: 0.94 to 0.98) | | |
| Pooled specificity | 0.74 (95% CI: 0.65 to 0.84) | | |
| **Consequences** | | | |
|  | **Number per 1000^1^** |  | **Importance** |
| **TP^2^** | 192 |  | 8 |
| **TN^3^** | 592 |  | 8 |
| **FP^4^** | 208 |  | 7 |
| **FN^5^** | 8 |  | 9 |
| **Inconclusive results^6,7^** | – |  | 5 |
| **Cost^7^** | – |  | 5 |

^1^ all results are given per 1000 patients tested based on the prevalence of 20% and pooled sensitivity and specificity.

^6^ inconclusive results are either uninterpretable, indeterminate or intermediate test results

^2^ Important because mandates drugs, angioplasty and stents, bypass surgery.

^3^ Important because spares patients unnecessary interventions associated with adverse effects.

^4^ Important because patients are exposed to unnecessary potential adverse effects from drugs and invasive procedures.

^5^ Important because increase risk of coronary events as a result of patients not receiving efficacious treatment.

^6^ Uninterpretable, indeterminate, or intermediate test results; important because generate anxiety, uncertainty as to how to proceed, further testing, and possible negative consequences of either treating or not treating.

^7^ Although the results for these consequences are not reported because they are not exactly known on the basis of the available data, they are important.

Appendix B S1: interview packages

Full 30-min interview

Summary of Findings tables for diagnostic test accuracy reviews – Interview Guide

| **Test participant No.:** |  |
| --- | --- |
| Participant name and contact information: | Name:  Email/Telephone: |
| Location: |  |
| Date: |  |
| Interviewer/Note taker: |  |
| Recorder interview No. and Total Time  *(e.g. 01, 1h12min)*: |  |

**A. Interviewer Checklist**

***For in person interviews:***

- Printed copy of all SoF table formats to be evaluated
- Interview Guide form. Take notes (point-form preferred) in the spaces provided.
- Additional paper to take notes if needed.
- Audio recorder. Test the recorder before each interview (Press REC to record a test message, then press STOP. Press PLAY to make sure the message was recorded clearly and is audible and press ERASE to delete the test message)

***For interviews by telephone or Skype:***

- Make sure you have a land line phone number or a Skype username
- For interviews by Skype, search for and add in the username of the interviewee in advance
- Send in advance a pdf copy of all the SoF table formats to be evaluated
- Confirm that the participant has received the SoF tables by email
- Confirm the date and time of the call, and send an email explaining that he/she needs to have the tables available
- Send a reminder to the participant two days before the meeting
- Interview Guide form
- Additional paper to take notes if needed.
- Audiorecorder for telephone/Recording Software for Skype. Test audio recorder before each interview.

**B. Introduction – 1 minute**

**What we are testing and why:**

**Say:** In this user testing interview, we will be evaluating the usability and understanding of Summary of Findings tables, referred to as “SoF” tables for short. Summary of Findings tables are used in the GRADE approach to summarize and present data from systematic reviews. They are intended to facilitate access to the key information of a systematic review. The format that SoF tables should have in diagnostic test accuracy (DTA) reviews has not been fully explored.

We will use your feedback to help determine the ideal presentation of information and the preferred content in SoF tables for diagnostic tests. We really appreciate you giving us a bit of your time.

**Participant Consent Statement:**

**Say:** The research study has been reviewed by the Hamilton Health Sciences Research Ethics Board. With your permission, the session will be recorded on tape for transcription and erased afterwards. Do you agree to have the interview recorded and for the data collected in the study to be used anonymously in publication?

Yes  No Notes:

Is it ok to contact you in the future if we have any questions?

Yes  No Notes:

***> As soon as you have finished the introduction, TURN ON AUDIORECORDER.***

**C. Background questions – 3 minutes**

**Say:** I will first ask a few questions about your background.

1. **Ask:** What is your current position?:

1. **Ask:** What is your formal education?: *(e.g. MSc, PhD Edpidemiology, Health Economics, etc.)*

1. **Ask:** We would also like to know about your professional background, and you may fit into more than one of the following categories, so select all that apply.

***Note to Interviewer***: For interviews with DTA review authors, skip C, for interviews with Clinicians skip B and ask directly about specialty:

1. Would you identify yourself as a **Researcher**:  Yes  No
2. Would you identify yourself as a **Health Professional**:  Yes  No

Please specify (*e.g. profession or clinical specialty*):

1. Are you an **author of DTA systematic review(s)**:  Yes  No
2. Are there any other roles that you have had that you would identify as relevant to this interview (e.g. guideline developer) (Specify):

1. How long have you been in these roles, in other words how many years of experience do you have overall?: *Years*

***Note to Interviewer***: For authors of DTA reviews, skip to Q6.

**Say:** For the next set of questions, I will ask about your experience with systematic reviews. We have provided an Answer Options Sheet as a guide. Please refer to the **BLUE section** of the sheet to help you choose the appropriate answers.

**Ask:** To what extent do you agree or disagree with the following statement?

1. I am familiar with systematic reviews.

Note that in the introduction I mentioned the topic of diagnostic testing, but here I am referring to systematic reviews on any topic, not DTA reviews specifically.

Strongly Disagree Somewhat Neither Agree Somewhat Agree Strongly

Disagree Disagree or Disagree Agree Agree

1. Approximately how many systematic reviews do you read per week, or per month, or per year? You can answer this question using the time frame you prefer:

*Circle time frame used:*  [per week] [per month] [per year]

1. Approximately how often do you access the Cochrane Library per week, or per month, or per year? You can also answer this question using the time frame you prefer:

*Circle time frame used:*  [per week] [per month] [per year]

1. For what purpose do you read/use systematic reviews?:

***Note to interviewer:*** *If the participant has difficulty answering, provide the cue*: Someone might read a systematic review for their own learning, or to make policy decisions, to make clinical decisions for a patient, or to make recommendations for clinical practice guidelines or coverage decisions.

***Note to Interviewer***: For authors of DTA reviews, skip to Q9.

**Say:** Refer again to the **BLUE section** of the answer sheet, to what extent do you agree or disagree with the following statement.

1. I am familiar with diagnostic test accuracy (DTA) systematic reviews.

Strongly Disagree Somewhat Neither Agree Somewhat Agree Strongly

Disagree Disagree or Disagree Agree Agree

1. Would you identify yourself as a **user of DTA systematic reviews**:  Yes  No

**If Yes**, in what capacity have you used DTA systematic reviews (e.g. to make clinical decisions for patients, to inform policies, to inform guideline developers)?:

[Alternative question f*or authors of DTA reviews* if have difficulty answering: In what capacity are the DTA reviews that you have produced intended to be used by others?]

How many times have you participated in this role:

**Say:** Referring to the **BLUE section** of the answer sheet, to what extent do you agree or disagree with the following statements.

1. I am familiar with the GRADE approach.

Strongly Disagree Somewhat Neither Agree Somewhat Agree Strongly

Disagree Disagree or Disagree Agree Agree

1. I am familiar with using Summary of Findings tables.

Strongly Disagree Somewhat Neither Agree Somewhat Agree Strongly

Disagree Disagree or Disagree Agree Agree

**D. Instructions for Evaluation of SoF Formats – 30 Seconds**

**Say:**

I will present to you alternative formats of the SoF tables with different ways of presenting information. For the questions I will ask you, think out loud, we want to capture your opinion. If you think something is easy or difficult, clear or confusing, please point it out. There are no right or wrong answers, we are not testing your knowledge, we are testing our material.

**E. Review SoF Format 1 – Layout 1 – 7 minutes**

**Say**: Please refer to the printout of SoF Format 1 – Layout 1 on Page 1. Review this format of the SoF table for about 2 minutes to grasp the information being presented. Let me know when you are ready. (***Note****:* Time the 2 minutes and notify participant when time is up.)

1. **Ask:**
2. What is your overall impression of this SoF table?
3. Which features of the table helped you to understand the systematic review data displayed? Which features were difficult to understand?

***Note to Interviewer:*** If interviewee is unsure provide cue: What do you think about the table overall, do you understand everything the table is presenting? Does it present all the information you think is essential for reporting and making conclusions about the test?

1. For Clinicians: Would you recommend or use this diagnostic test?

For DTA review authors: What would your conclusion about the test be given the results presented in the SoF table?

Yes  No  Need More Information

Why or why not? What information did you use to come to your conclusion?:

***Note to Interviewer:*** This question is to gauge the overall understanding of the table. We would like to know what components of the table the user looked at and considered in providing their answer. We want to see if some users for example find only sensitivity and specificity sufficient and do not consider individual DTA results or impact on patient-important outcomes. ‘Need more information’ could refer to information on purpose of the test, place of the test in the test-treatment strategy, or downstream consequences of test results or treatment.

1. Did you initially read the content in the footnotes when reviewing the table?:

Yes  No  Why or Why Not?:

***Note to Interviewer:*** If answer above is yes, and the answer addresses the follow-up to Q4 below, then ask only the question on the 7-point scale for Q4, if not addressed then ask the follow-up questions.

**Say:** If you did not initially read the footnotes, have a look through them now. For the next question, referring to the **RED section** of the answer sheet, to what extent do you agree or disagree with this statement.

1. The content in the footnotes helped me to better understand the data.

Strongly Disagree Somewhat Neither Agree Somewhat Agree Strongly

Disagree Disagree or Disagree Agree Agree

Why? What information were you looking for in the footnotes to help with understanding and interpretation of the data in the table? Did the footnotes provide the information you expected to see?

**F. Review SoF Format 1 – Layout 2 – 7 minutes**

**Say**: We have also prepared a second layout for this SoF format. Please refer to the printout of SoF Format 1 – Layout 2 on Page 2. Review this layout for about 1 minute. This layout uses the same systematic review example as the previous one.

**Say**: Referring to the **GREEN section** of the answer sheet, to what extent do you agree or disagree with these statements.

1. The purpose of the two different prevalence values presented was clear to me.

Strongly Disagree Somewhat Neither Agree Somewhat Agree Strongly

Disagree Disagree or Disagree Agree Agree

Why? For what reason did you think two prevalence estimates are presented in this SoF table layout?

1. The presentation of various prevalence estimates is important for readers of the systematic review to help make appropriate conclusions and demonstrate diagnostic test accuracy results in different clinical settings.

Strongly Disagree Somewhat Neither Agree Somewhat Agree Strongly

Disagree Disagree or Disagree Agree Agree

For Clinicians: How do you think the presentation of different prevalence values affects making conclusions about the test? How might your conclusion about the test change when presented with the different prevalence estimates?

For DTA authors: How do you think the presentation of different prevalence values affects making appropriate conclusions about the test by the users of the systematic review?

1. Do you think that an example clinical scenario should be presented in a SoF table to accompany the prevalence value estimate?

Yes, present clinical scenario  No, clinical scenario is unnecessary

If **NO**, why do you think the scenario is not necessary?

1. Could the clinical scenario information be placed in the footnotes instead of inside the table?

Yes, place in footnotes  No, leave in table  Other answer

What is the reason for your preference?

**Say**: I will now ask about your overall preference between the two layouts we have reviewed.

1. Do you prefer Layout 1 (Pg 1) with one prevalence estimate and no clinical scenario, or Layout 2 (Pg 2) with two prevalence estimates and the clinical scenarios?:

Layout 1  Layout 2

1. What is the reason for your preference?
2. Would you change anything about the overall presentation or look of either layout?

**G. Review SoF Format 2 – 2 minutes**

**Say:** We will now review an alternative format of the SoF table. Please refer to the printout of SoF Format 2 on Page 3. Review this format for about 30 seconds to familiarize yourself with it. This SoF format uses the same systematc review example as the first format.

1. **Ask:**
2. What is your overall impression of this format?
3. How does the arrangement of the rows in the table by test-positive and test-negative (versus according to sensitivity and specificity as in the first format) affect your interpretation of the test results?
4. Does this arrangement help or hinder using the SoF table for making conclusions about the test?

**H. Review SoF Format 3 – Layouts 1-3 – 5 minutes**

**Say:** We will now review a third alternative format of the SoF table. This SoF table is a bit different from the first two formats you have reviewed, but uses the same systematic review example. Please refer to the printout of SoF Table Format 3 – Layout 1 on Page 4 and review it for about 1 minute. Let me know when you are ready.

1. **Ask**:
2. What is your overall impression of this format?
3. How do the features of this table affect your understanding and interpretation of the systematic review results when compared to the features in the previous two SoF formats?
4. Which features of this table are more and less helpful? Is there any information that is missing?

1. **Ask**:
2. How do you think the presentation of post-test probability and likelihood ratios affects making appropriate conclusions about the test by the users of the systematic review?
3. Do the likelihood ratios provide important information, or is post-test probability the more important piece of information in this table?

**Say**: We have also prepared two other layouts for this SoF format. Please refer to the printouts of SoF Format 3 – Layout 2 on Page 5 and SoF Format 3 – Layout 3 on Page 6. I will now ask about your overall preference between the three layouts.

1. Do you prefer prefer Layout 1 (Pg 4) with no clinical scenarios, Layout 2 (Pg 5) with the clinical scenarios, or Layout 3 (Pg 6) with the clinical scenarios and the likelihood ratios placed in the header of the table instead of as a column inside the table?

Layout 1  Layout 2  Layout 3

1. What is the reason for your preference?
2. Would you change anything about the overall presentation or look of these layouts?
3. If you prefer the layouts with the clinical scenario, where should the clinical scenario be placed in this SoF format?

**I. Review SoF Format 4 – 2 minutes**

**Say:** We will now review the last alternative format. Please refer to the printout of SoF Table Format 4 on Page 7 and review it for about 30 seconds. Let me know when you are ready.

**Say:** Referring to the **Yellow section** of the answer sheet, to what extent do you agree with the following statement?

1. The table provides sufficient information for understanding the systematic review results and making conclusions about the diagnostic test.

Strongly Disagree Somewhat Neither Agree Somewhat Agree Strongly

Disagree Disagree or Disagree Agree Agree

1. **Ask**:
2. What is your overall impression of this format?
3. Is this table sufficient to present DTA systematic review results? Why or why not?
4. What information is missing that is essential for understanding the results and making an appropriate conclusion about the diagnostic test?

**J. Overall Preferences – 2 minutes**

**Say:** Having viewed all the formats, I will now ask you about your overall preferences.

1. **Ask**:
2. Overall, do you prefer prefer SoF Format 1 (Pg 1 & 2, with rows arranged by sensitivity and specificity), Format 2 (Pg 3, with rows arranged by test-positive and test-negative), Format 3 (Pgs 4-6, with presentation of post-test probability and likelihood ratios), or Format 4 (Pg 7, with presentation of sensitivity and specificity).
3. Rank the other formats in order of preference. You can choose not to rank a certain format if you feel that it is not useful at all and should not be used.

Format 1 (by sens/spec): Format 2 (by test +/-):

Format 3 (LR): Format 4 (sens/spec only):

What is the reason for your overall preference and the rankings you assigned? Which features of the tables were you taking into consideration in deciding your preferences?

**Say:** Considering the SoF tables overall and referring to the **Yellow section** of the answer sheet, to what extent do you agree with the following statement?

1. Presenting the SoF table as part of a DTA systematic review is a helpful way of reporting the outcomes.

Strongly Disagree Somewhat Neither Agree Somewhat Agree Strongly

Disagree Disagree or Disagree Agree Agree

Why or why not? Does this table represent what you would expect from a SoF to help with interpretation of the results and making appropriate conclusions about a diagnostic test? Do you think a SoF table should be included in a DTA review?

**K. Conclusion – 1 minute**

- - 1. **Ask:** Do you have any final comments that you would like to make about the content and presentation of information in SoF tables, keeping in mind that the goal of this interview was to identify problematic areas for users and to determine the usefulness and usability of the tables for presentation of DTA systematic review data?

**Say:** That marks the end of our interview. Thank you very much for your participation, we really appreciate you dedicating your time to this.

| Galactomannan Elisa For The Diagnosis Of Invasive Aspergillosis | | | |
| --- | --- | --- | --- |
| **Patients or population:** immunocompromized patients, mostly hematology patients^1^ **Settings:** mainly hematology or cancer departments, mainly inpatients **New Test:** commercial Platelia® sandwich ELISA detecting galactomannan in serum^2^ \| **Cut-off value:** 1.5 ODI  **Reference Test:** composite of EORTC/MSG clinical and histological criteria^3^ \| **Threshold:** Proven or probable invasive aspergillosis | | | |
| **Test result** | Number of results  per 1000 patients tested  (95% CI) | Number  of participants  (studies) | Quality  of the evidence (GRADE) |
|  | Assumed numbers with galactomannan ELISA compared to reference test (EORTC/MSG criteria) |  |  |
|  | Prevalence 20 per 1000^4^ |  |  |
| Sensitivity (95% CI): 0.64 (0.50 to 0.77) | | 2777  (18 studies) | ⊕⊕⊕⊕  High^5^ |
| True positives | 13 per 1000  (9 to 15 per 1000) |  |  |
| False negatives | 7 per 1000  (4 to 10 per 1000) |  |  |
| Specificity (95% CI): 0.95 (0.91 to 0.97) | |  |  |
| True negatives | 931 per 1000  (901 to 960 per 1000) |  |  |
| False positives | 49 per 1000  (30 to 89 per 1000) |  |  |
| CI: Confidence interval; ODI: Optical density index; EORTC: European Organization for Research and Treatment of Cancer; MSG: Mycoses Study Group | | | |
| Leeflang M, Debets-Ossenkopp Y, Visser C, Scholten R, Hooft L, Bijlmer H, et al. Galactomannan detection for invasive aspergillosis in immunocompromized patients. Cochrane Database of Systematic Reviews. 2008; 4 (Art. No.: CD007394). | | | |
| Footnotes:  ^1^ One study that included only patients with HIV/AIDS was excluded, because this patient group and setting differed from the other included patient groups in such a way that authors of the review regarded the study population as not being representative.  ^2^ A diagnostic test for invasive aspergillosis (IA) needs to be not too invasive or a burden to immunocompromised patients. The galactomannan ELISA test on a serum specimen is less burdensome than the current reference standard.  ^3^ A true reference standard is autopsy combined with culture from autopsy specimens; a clinical reference standard is composed of EORTC/MSG clinical and histological criteria.  ^4^ Estimate of prevalence of IA was based on the median and range of values in included studies.  ^5^ Included studies were valid diagnostic accuracy studies in patients with diagnostic uncertainty and direct comparison of test results with an appropriate reference standard. Quality was not downgraded based on the GRADE criteria. | | | |

| **Galactomannan ELISA for the diagnosis of invasive aspergillosis** | | | | |
| --- | --- | --- | --- | --- |
| Patients or population: immunocompromized patients, mostly hematology patients^1^ Settings: mainly hematology or cancer departments, mainly inpatients New Test: commercial Platelia® sandwich ELISA detecting galactomannan in serum^2^ \| Cut-off value: 1.5 ODI  Reference Test: composite of EORTC/MSG clinical and histological criteria^3^ \| Threshold: Proven or probable invasive aspergillosis | | | | |
| **Test result** | Number of results  per 1000 patients tested^4^  (95% CI) | | Number of participants  (studies) | Quality  of the evidence (GRADE) |
|  | Prevalence 20 per 1000^5^:  Which is typically seen in adults undergoing transplant, no neutropenic patients. | Prevalence 400 per 1000^5^:  Which is typically seen in adults with hematological disorders that were neutropenic, underwent chemotherapy, had persistent fever despite antibiotics, acute graft versus host disease, or received corticosteroids. |  |  |
| Sensitivity (95% CI): 0.64 (0.50 to 0.77) | | | 2777  (18 studies) | ⊕⊕⊕⊕  High^6^ |
| True positives | 13 per 1000  (9 to 15 per 1000) | 282 per 1000  (220 to 339 per 1000) |  |  |
| False negatives | 7 per 1000  (4 to 10 per 1000) | 158 per 1000  (101 to 220 per 1000) |  |  |
| Specificity (95% CI): 0.95 (0.91 to 0.97) | | |  |  |
| True negatives | 931 per 1000  (901 to 960 per 1000) | 532 per 1000  (510 to 543 per 1000) |  |  |
| False positives | 49 per 1000  (30 to 89 per 1000) | 28 per 1000  (17 to 50 per 1000) |  |  |
| CI: Confidence interval; ODI: Optical density index; EORTC: European Organization for Research and Treatment of Cancer; MSG: Mycoses Study Group | | | | |
| Leeflang M, Debets-Ossenkopp Y, Visser C, Scholten R, Hooft L, Bijlmer H, et al. Galactomannan detection for invasive aspergillosis in immunocompromized patients. Cochrane Database of Systematic Reviews. 2008; 4 (Art. No.: CD007394). | | | | |
| Footnotes:  ^1^ One study that included only patients with HIV/AIDS was excluded, because this patient group and setting differed from the other included patient groups in such a way that authors of the review regarded the study population as not being representative.  ^2^ A diagnostic test for invasive aspergillosis (IA) needs to be not too invasive or a burden to immunocompromised patients. The galactomannan ELISA test on a serum specimen is less burdensome than the current reference standard.  ^3^ A true reference standard is autopsy combined with culture from autopsy specimens; a clinical reference standard is composed of EORTC/MSG clinical and histological criteria.  ^4^ Assumed numbers with galactomannan ELISA compared to reference test (EORTC/MSG criteria).  ^5^ Estimates of prevalence of IA were based on the median and range of values in included studies.  ^6^ Included studies were valid diagnostic accuracy studies in patients with diagnostic uncertainty and direct comparison of test results with an appropriate reference standard. Quality was not downgraded based on the GRADE criteria. | | | | |

| **Galactomannan ELISA for the diagnosis of invasive aspergillosis** | | | | | |
| --- | --- | --- | --- | --- | --- |
| Patients or population: immunocompromized patients, mostly hematology patients^1^ Settings: mainly hematology or cancer departments, mainly inpatients New Test: commercial Platelia® sandwich ELISA detecting galactomannan in serum^2^ \| Cut-off value: 1.5 ODI  Reference Test: composite of EORTC/MSG clinical and histological criteria^3^ \| Threshold: Proven or probable invasive aspergillosis | | | | | |
| Pooled Sensitivity (95% CI): 0.64 (0.50 to 0.77) \| Pooled Specificity (95% CI): 0.95 (0.91 to 0.97) | | | | | |
| **Test result** | Number of results  per 1000 patients tested^4^  (95% CI) | | | Number of participants  (studies) | Quality  of the evidence (GRADE) |
|  | Prevalence 20 per 1000^5^:  Which is typically seen in adults undergoing transplant, no neutropenic patients. | Prevalence 400 per 1000^5^:  Which is typically seen in adults with hematological disorders that were neutropenic, underwent chemotherapy, had persistent fever despite antibiotics, acute graft versus host disease, or received corticosteroids. | |  |  |
| Test-Positive | | | | 2777  (18 studies) | ⊕⊕⊕⊕  High^6^ |
| True positives | 13 per 1000  (9 to 15 per 1000) | 282 per 1000  (220 to 339 per 1000) | |  |  |
| False positives | 49 per 1000  (30 to 89 per 1000) | 28 per 1000  (17 to 50 per 1000) | |  |  |
| Test-Negative | | | |  |  |
| True negatives | 931 per 1000  (901 to 960 per 1000) | | 532 per 1000  (510 to 543 per 1000) |  |  |
| False negatives | 7 per 1000  (4 to 10 per 1000) | | 158 per 1000  (101 to 220 per 1000) |  |  |
| CI: Confidence interval; ODI: Optical density index; EORTC: European Organization for Research and Treatment of Cancer; MSG: Mycoses Study Group | | | | | |
| Leeflang M, Debets-Ossenkopp Y, Visser C, Scholten R, Hooft L, Bijlmer H, et al. Galactomannan detection for invasive aspergillosis in immunocompromized patients. Cochrane Database of Systematic Reviews. 2008; 4 (Art. No.: CD007394). | | | | | |
| Footnotes:  ^1^ One study that included only patients with HIV/AIDS was excluded, because this patient group and setting differed from the other included patient groups in such a way that authors of the review regarded the study population as not being representative.  ^2^ A diagnostic test for invasive aspergillosis (IA) needs to be not too invasive or a burden to immunocompromised patients. The galactomannan ELISA test on a serum specimen is less burdensome than the current reference standard.  ^3^ A true reference standard is autopsy combined with culture from autopsy specimens; a clinical reference standard is composed of EORTC/MSG clinical and histological criteria.  ^4^ Assumed numbers with galactomannan ELISA compared to reference test (EORTC/MSG criteria).  ^5^ Estimates of prevalence of IA were based on the median and range of values in included studies.  ^6^ Included studies were valid diagnostic accuracy studies in patients with diagnostic uncertainty and direct comparison of test results with an appropriate reference standard. Quality was not downgraded based on the GRADE criteria. | | | | | |

| **Galactomannan ELISA for the diagnosis of invasive aspergillosis** | | | | | |
| --- | --- | --- | --- | --- | --- |
| Patients or population: immunocompromized patients, mostly hematology patients^1^ Settings: mainly hematology or cancer departments, mainly inpatients New Test: commercial Platelia® sandwich ELISA detecting galactomannan in serum^2^ \| Cut-off value: 1.5 ODI  Reference Test: composite of EORTC/MSG clinical and histological criteria^3^ \| Threshold: Proven or probable invasive aspergillosis | | | | | |
| Pooled Sensitivity (95% CI): 0.64 (0.50 to 0.77) \| Pooled Specificity (95% CI): 0.95 (0.91 to 0.97) | | | | | |
| Pre-test  probability^4^ | Test result | Post-test probability^5^  (95% CI) | Likelihood ratio (LR)  (95% CI) | Number  of participants (studies) | Quality  of the evidence (GRADE) |
| Low Probability | | | | 2777  (18 studies) | ⊕⊕⊕⊕  High^6^ |
| 2% | + | 21%  (17% to 26%) | + LR = 13  (10 to 17) |  |  |
|  | – | 1%  (1% to 1%) | – LR = 0.38  (0.27 to 0.54) |  |  |
| High Probability | | | |  |  |
| 44% | + | 91%  (89% to 93%) | + LR = 13  (10 to 16) |  |  |
|  | – | 23%  (22% to 24%) | – LR = 0.38  (0.35 to 0.41) |  |  |
| CI: Confidence interval; ODI: Optical density index; EORTC: European Organization for Research and Treatment of Cancer; MSG: Mycoses Study Group | | | | | |
| Leeflang M, Debets-Ossenkopp Y, Visser C, Scholten R, Hooft L, Bijlmer H, et al. Galactomannan detection for invasive aspergillosis in immunocompromized patients. Cochrane Database of Systematic Reviews. 2008; 4 (Art. No.: CD007394). | | | | | |
| Footnotes:  ^1^ One study that included only patients with HIV/AIDS was excluded, because this patient group and setting differed from the other included patient groups in such a way that authors of the review regarded the study population as not being representative.  ^2^ A diagnostic test for invasive aspergillosis (IA) needs to be not too invasive or a burden to immunocompromised patients. The galactomannan ELISA test on a serum specimen is less burdensome than the current reference standard.  ^3^ A true reference standard is autopsy combined with culture from autopsy specimens; a clinical reference standard is composed of EORTC/MSG clinical and histological criteria.  ^4^ Pre-test probablity of IA was selected based on the median and range of prevalence values in included studies.  ^5^ Percentages are rounded to the nearest integer.  ^6^ Included studies were valid diagnostic accuracy studies in patients with diagnostic uncertainty and direct comparison of test results with an appropriate reference standard. Quality was not downgraded based on the GRADE criteria. | | | | | |

| **Galactomannan ELISA for the diagnosis of invasive aspergillosis** | | | | | |
| --- | --- | --- | --- | --- | --- |
| Patients or population: immunocompromized patients, mostly hematology patients^1^ Settings: mainly hematology or cancer departments, mainly inpatients New Test: commercial Platelia® sandwich ELISA detecting galactomannan in serum^2^ \| Cut-off value: 1.5 ODI  Reference Test: composite of EORTC/MSG clinical and histological criteria^3^ \| Threshold: Proven or probable invasive aspergillosis | | | | | |
| Pooled Sensitivity (95% CI): 0.64 (0.50 to 0.77) \| Pooled Specificity (95% CI): 0.95 (0.91 to 0.97) | | | | | |
| **Pre-test  probability^4^** | Test result | Post-test probability^5^  (95% CI) | Likelihood ratio (LR)  (95% CI) | Number  of participants (studies) | Quality  of the evidence (GRADE) |
| Low Probability | | | | 2777  (18 studies) | ⊕⊕⊕⊕  High^6^ |
| 2%  - - - - - - - - - - - - - - - - -  Which is typically seen in adults undergoing transplant, no neutropenic patients | + | 21%  (17% to 26%) | + LR = 13  (10 to 17) |  |  |
|  | – | 1%  (1% to 1%) | – LR = 0.38  (0.27 to 0.54) |  |  |
| High Probability | | | |  |  |
| 44%  - - - - - - - - - - - - - - - - -  Which is typically seen in adults with hematological disorders that were neutropenic, underwent chemotherapy, had persistent fever despite antibiotics, acute graft versus host disease, or received corticosteroids. | + | 91%  (89% to 93%) | + LR = 13  (10 to 16) |  |  |
|  | – | 23%  (22% to 24%) | – LR = 0.38  (0.35 to 0.41) |  |  |
| CI: Confidence interval; ODI: Optical density index; EORTC: European Organization for Research and Treatment of Cancer; MSG: Mycoses Study Group | | | | | |
| Leeflang M, Debets-Ossenkopp Y, Visser C, Scholten R, Hooft L, Bijlmer H, et al. Galactomannan detection for invasive aspergillosis in immunocompromized patients. Cochrane Database of Systematic Reviews. 2008; 4 (Art. No.: CD007394). | | | | | |
| Footnotes:  ^1^ One study that included only patients with HIV/AIDS was excluded, because this patient group and setting differed from the other included patient groups in such a way that authors of the review regarded the study population as not being representative.  ^2^ A diagnostic test for invasive aspergillosis (IA) needs to be not too invasive or a burden to immunocompromised patients. The galactomannan ELISA test on a serum specimen is less burdensome than the current reference standard.  ^3^ A true reference standard is autopsy combined with culture from autopsy specimens; a clinical reference standard is composed of EORTC/MSG clinical and histological criteria.  ^4^ Pre-test probablity of IA was selected based on the median and range of prevalence values in included studies.  ^5^ Percentages are rounded to the nearest integer.  ^6^ Included studies were valid diagnostic accuracy studies in patients with diagnostic uncertainty and direct comparison of test results with an appropriate reference standard. Quality was not downgraded based on the GRADE criteria. | | | | | |

| **Galactomannan ELISA for the diagnosis of invasive aspergillosis** | | | | |
| --- | --- | --- | --- | --- |
| Patients or population: immunocompromized patients, mostly hematology patients^1^ Settings: mainly hematology or cancer departments, mainly inpatients New Test: commercial Platelia® sandwich ELISA detecting galactomannan in serum^2^ \| Cut-off value: 1.5 ODI  Reference Test: composite of EORTC/MSG clinical and histological criteria^3^ \| Threshold: Proven or probable invasive aspergillosis | | | | |
| Pooled Sensitivity (95% CI): 0.64 (0.50 to 0.77) \| Pooled Specificity (95% CI): 0.95 (0.91 to 0.97) | | | | |
| Likelihood ratio (LR) (95% CI): + LR = 13 (10 to 17) , – LR = 0.38 (0.27 to 0.54) | | | | |
| **Pre-test  probability^4^** | Test result | Post-test probability^5^  (95% CI) | Number  of participants (studies) | Quality  of the evidence (GRADE) |
| Low Probability | | | 2777  (18 studies) | ⊕⊕⊕⊕  High^6^ |
| 2%  - - - - - - - - - - - - - - - - - -  Which is typically seen in adults undergoing transplant, no neutropenic patients. | + | 21%  (17% to 26%) |  |  |
|  | – | 1%  (1% to 1%) |  |  |
| High Probability | | |  |  |
| 44%  - - - - - - - - - - - - - - - - - -  Which is typically seen in adults with hematological disorders that were neutropenic, underwent chemotherapy, had persistent fever despite antibiotics, acute graft versus host disease, or received corticosteroids. | + | 91%  (89% to 93%) |  |  |
|  | – | 23%  (22% to 24%) |  |  |
| CI: Confidence interval; ODI: Optical density index; EORTC: European Organization for Research and Treatment of Cancer; MSG: Mycoses Study Group | | | | |
| Leeflang M, Debets-Ossenkopp Y, Visser C, Scholten R, Hooft L, Bijlmer H, et al. Galactomannan detection for invasive aspergillosis in immunocompromized patients. Cochrane Database of Systematic Reviews. 2008; 4 (Art. No.: CD007394). | | | | |
| Footnotes:  ^1^ One study that included only patients with HIV/AIDS was excluded, because this patient group and setting differed from the other included patient groups in such a way that authors of the review regarded the study population as not being representative.  ^2^ A diagnostic test for invasive aspergillosis (IA) needs to be not too invasive or a burden to immunocompromised patients. The galactomannan ELISA test on a serum specimen is less burdensome than the current reference standard.  ^3^ A true reference standard is autopsy combined with culture from autopsy specimens; a clinical reference standard is composed of EORTC/MSG clinical and histological criteria.  ^4^ Pre-test probablity of IA was selected based on the median and range of prevalence values in included studies.  ^5^ Percentages are rounded to the nearest integer.  ^6^ Included studies were valid diagnostic accuracy studies in patients with diagnostic uncertainty and direct comparison of test results with an appropriate reference standard. Quality was not downgraded based on the GRADE criteria. | | | | |

| **Galactomannan ELISA for the diagnosis of invasive aspergillosis** | | | |
| --- | --- | --- | --- |
| Patients or population: immunocompromized patients, mostly hematology patients^1^ Settings: mainly hematology or cancer departments, mainly inpatients New Test: commercial Platelia® sandwich ELISA detecting galactomannan in serum^2^ \| Cut-off value: 1.5 ODI  Reference Test: composite of EORTC/MSG clinical and histological criteria^3^ \| Threshold: Proven or probable invasive aspergillosis | | | |
| **Test**  **property** | Summary estimate  (95% CI) | Number of  participants  (studies) | Quality  of the evidence  (GRADE) |
| Sensitivity | 0.64  (0.50 to 0.77) | 2777  (18 studies) | ⊕⊕⊕⊕  High^4^ |
| Specificity | 0.95  (0.91 to 0.97) |  |  |
| CI: Confidence interval; ODI: Optical density index; EORTC: European Organization for Research and Treatment of Cancer; MSG: Mycoses Study Group | | | |
| Leeflang M, Debets-Ossenkopp Y, Visser C, Scholten R, Hooft L, Bijlmer H, et al. Galactomannan detection for invasive aspergillosis in immunocompromized patients. Cochrane Database of Systematic Reviews. 2008; 4 (Art. No.: CD007394). | | | |
| Footnotes:  ^1^ One study that included only patients with HIV/AIDS was excluded, because this patient group and setting differed from the other included patient groups in such a way that authors of the review regarded the study population as not being representative.  ^2^ A diagnostic test for invasive aspergillosis (IA) needs to be not too invasive or a burden to immunocompromised patients. The galactomannan ELISA test on a serum specimen is less burdensome than the current reference standard.  ^3^ A true reference standard is autopsy combined with culture from autopsy specimens; a clinical reference standard is composed of EORTC/MSG clinical and histological criteria.  ^4^ Included studies were valid diagnostic accuracy studies in patients with diagnostic uncertainty and direct comparison of test results with an appropriate reference standard. Quality was not downgraded based on the GRADE criteria. | | | |

Full interview

Summary of Findings tables for diagnostic test accuracy reviews – Interview Guide

A**. Interviewer Checklist**

**For in person interviews:**

- Printed copy of all SoF table formats to be evaluated

- Interview Guide form. Take notes (point-form preferred) in the spaces provided.

- Additional paper to take notes if needed.
- Audiorecorder. Test the recorderbefore each interview (Press REC to record a test message, then press STOP. Press PLAY to make sure the message was recorded clearly and is audible and press ERASE to delete the test message)

| **Test participant No.:** |  |
| --- | --- |
| Participant name and contact information: | Name:____________________________________  _________________________________________  Email/Telephone: ____________________________  _________________________________________ |
| Location: |  |
| Date: |  |
| Interviewer/Notetaker: |  |
| Recorder interview No. and Total Time  *(e.g. 01, 1h12min)*: |  |

**For interviews by telephone or Skype:**

- Make sure you have a land line phone number or a Skype username
- For interviews by Skype, search for and add in the username of the interviewee in advance
- Send in advance a pdf copy of all the SoF table formatss to be evaluated
- Confirm that the participant has received the SoF tables by email
- Confirm the date and time of the call, and send an email explaining that he/she needs to have the tables available
- Send a reminder to the participant two days before the meeting
- Interview Guide form
- Additional paper to take notes if needed.
- Audiorecorder for telephone/Recording Software for Skype. Test audio recorder before each interview.

***Note to interviewer:*** *All the introductory material and background questions (Sections B-D) should take a total of 10 minutes.*

**B. Introduction – 4 minutes**

**Say:** You have been asked to participate in a user testing interview to help provide feedback on material that is intended to help with presenting information of diagnostic test accuracy (DTA) reviews. We really appreciate you giving us a bit of your time.

**What we are testing and why:**

**Say:** We will be evaluating the usability and usefulness of Summary of Findings tables. I will refer to these as “SoF” tables for short throughout the interview. Summary of Findings tables are used in the GRADE approach to summarize and present outcome data from systematic reviews and are now a key element of Cochrane Intervention Systematic Reviews. They are are intended to accompany a systematic review and be a tool to support tranparent and facilitate access to the key information of a systematic review. The format that SoF Tables should have in DTA reviews is incompletely explored.

You are one of about 20 people from a variety of backgrounds that represent users of systematic reviews, including clinicians, researchers, guideline developers and decision makers, that we are collecting feedback from. We will use your feedback to help determine the prefererd content and presentation of information in Summary of Findings tables for diagnostic tests with the aim to maximize their usablity and usefulness for authors and users like you.

**What the interview session will consist of:**

**Say:** For this interview, I will first ask some questions about your background and experience with systematic reviews. Then, I will present to you alternative formats of the SoF table with different ways of presenting information in the tables. I will ask you questions about these different formats. Questions will be in the form of statements, with which you will be asked to agree or disagree, open ended questions seeking your comments, or questions asking about your preferences. We have provided you with an Answer Options sheet which will help with choosing the appropriate answer for some questions.

**Participant Consent:**

**Say:** The research study has been reviewed by the Hamilton Health Sciences Research Ethics Board. I have provided you with an introduction to the study and I have informed you about the study purpose and what is being asked of you as a participant. With your permission, the session will be recorded on tape. The recording will only be used for transcribing the interview and will be erased afterwards. Do you agree to have the interview recorded and for the data collected to be used for the research study and to be summarized anonymously in publication?

□ Yes □ No Notes:__________________________________

May we also contact you in the future in case we need to follow up with you about the interview, for example if we need to clarify any of your comments or if any of your comments did not get recorded properly?

□ Yes □ No Notes:__________________________________

**Ask:** Do you have any questions before we proceed?

**> As soon as you have finished the introduction, TURN ON AUDIORECORDER.**

**C. Background questions – 5 minutes**

**Say:** I will first ask a few questions about your background.

1. **Ask:** What is your current position?: _________**________________________**

**_______________________________________________________**

1. **Ask:** What is your formal education?: __________________________________

*(e.g. MSc, PhD Edpidemiology, Health Economics, etc.)*

1. **Ask:** We wouldalso like to know about your professional background, and you may fit into more than one of the following categories, so select all that apply. Of the following 3 options:
2. Would you identify yourself as a **Researcher**: □ Yes □ No
3. Would you identify yourself as a **Health Professional**: □ Yes □ No

Please specify (*e.g. profession or clinical specialty*):___________________________

__________________________________________________________

1. Are you an **author of DTA systematic review(s)**: □ Yes □ No
2. Are you a **user of DTA systematic review(s)**: □ Yes □ No

**If Yes**, in what capacity have you used DTA systematic reviews (e.g. to make clinical decisions fr patients, to inform policys, to inform guideline developers):? ________

__________________________________________________________

How many times have you participated in this role: _______________________

1. Are there any other roles that you have had that you would identify as relevant to this interview (Specify): ____________________________________________

__________________________________________________________

1. How long have you been in these roles, in other words how many years of experience do you have overall?: ________ *Years*

**Say:** For the next set of questions, I will ask about your experience with systematic reviews. These questions will mainly be in the form of statements for which I will ask whether you agree or disagree on a 7-point scale. Please refer to the Answer Options Sheet we have provided you, and refer to the BLUE section to help you choose the appropriate answers.

**Ask:** To what extent do you agree or disagree with the following statements.

1. **Say:** I am familiar with systematic reviews. Based on the information in the BLUE section of the answer sheet, the two endpoints for this question would be as follows. Someone who strongly disagrees is someone who has never used or read systematic reviews, or does not have any methods training in systematic reviews. Someone who strongly agrees is someone who frequently uses systematic reviews, or produces systematic review, or has methods training in systematic reviews. Note that in the introduction I mentioned the topic of diagnostic testing, but here I am referring to systematic reviews on any topic, not on diagnosis specifically. ***Note to Interviewer:*** *If needed, read out the 7 answer options.*

□ □ □ □ □ □ □

Strongly Disagree Somewhat Neither Agree Somewhat Agree Strongly

Disagree Disagree or Disagree Agree Agree

1. Approximately how many systematic reviews do you read per week, or per month, or per year? You can answer this question using the time frame you prefer:______________

*Circle time frame used:*  [per week] [per month] [per year]

1. Approximately how often do you access the Cochrane Library per week, or per month, or per year? You can also answer this question using the time frame you prefer:________

*Circle time frame used:*  [per week] [per month] [per year]

1. For what purpose do you read the systematic reviews?: _______________________

_____________________________________________________________

***Note to interviewer:*** *If the participant has difficulty answering, provide the cue*: Someone might read a systematic review for their own learning, or to make policy decisions, or to make recommendations for clinical practice guidelines or coverage decisions, or to make clinical decisions for a patient.

**Say:** For the next questions I will ask again to what extent do you agree with the statements. For these, refer also to the BLUE section of the answer sheet to help you choose the appropriate answers.

1. I am familiar with diagnostic test accuracy (DTA) systematic reviews. Please note that from now on I will refer to diagnostic test accuracy as “DTA” to simplify things. For this question, someone who strongly disagrees is someone who has never used or read DTA systematic reviews, or does not have any methods training in DTA systematic reviews. Someone who strongly agrees is someone who frequently uses DTA systematic reviews, or produces DTA systematic reviews, or has methods training in DTA systematic reviews.

□ □ □ □ □ □ □

Strongly Disagree Somewhat Neither Agree Somewhat Agree Strongly

Disagree Disagree or Disagree Agree Agree

1. I am familiar with the GRADE approach. Referring again to the BLUE section of the answer sheet, someone who strongly disagrees is someone who has never heard about, read, or used the GRADE approach. Someone who strongly agrees is someone who has received training in applying GRADE and has used the GRADE approach in practice.

□ □ □ □ □ □ □

Strongly Disagree Somewhat Neither Agree Somewhat Agree Strongly

Disagree Disagree or Disagree Agree Agree

1. I am familiar with using Summary of Findings (SoF) tables. For this question, someone who strongly disagrees is someone who has never read or used SoF tables. Someone who strongly agrees is someone who frequently uses SoF tables in decision-making or has produced SoF tables.

□ □ □ □ □ □ □

Strongly Disagree Somewhat Neither Agree Somewhat Agree Strongly

Disagree Disagree or Disagree Agree Agree

**D. Instructions for Evaluation of SoF Formats – 30 seconds**

**Say:**

For the following part of the interview we aim to find what works well and what doesn’t work well, both regarding content, use of language or terminology, as well as presentation and formating of the tables. We want to know what are any major problems for all users regardless of experience and are there any striking things that don’t make sense or are problematic. Think out loud, we want to capture your opinion. There are no right or wrong answers, we are not testing your knowledge, we are testing our material.

From our experience, we are fairly certain that things you find difficult to understand, other people will also find difficult. When we ask for your comments describe your interpretation of the tables, if you are unsure or surprised by anything, if there are things you don’t understand, just say “I don’t know what this means…”, if you think something is easy or difficult, clear or confusing, please point it out. This is the information we can use to improve the summary of findings tables.

My role is to ask the interview questions. But, since it is your opinion we are interested in, I will be otherwise saying as little as possible during this part of the interview.

**[Layer 1: (mandatory) assessing different formats of SoF tables to summarise DTA SR results]**

**E. Review Single Test SoF Format 1 – 7 minutes**

**Say**: First, please refer to the printout of SoF Format 1 – Layout 1 on Page 1. Review this format of the SoF table for about 2 minutes to grasp the information being presented. Let me know when you are ready. You will give us your thoughts by answering the following question, and we will go more in depth later in the interview regarding these topics:

***Note to interviewer****: Time the 2 minutes and notify participant when time is up.*

1. **Ask:** What is your overall impression of this SoF table format?

***Note to interviewer****: If interviewee is unsure of how to answer provide cue: What do you think about the table overall, do you understand everything the table is presenting, what do you think about it in terms of content and presentation/aesthetics?*

|  |
| --- |

1. **Ask:** Which features of the table helped you to better understand the systematic review data displayed? Are there any parts of the table that are not necesssary?

|  |
| --- |

1. **Ask:** Would you recommend or use this diagnostic test? Why or why not:

***Note to Interviewer:*** *This question is to gauge the overall understanding of the table. We would like to know what components of the table the user looked at and considered in making the decision. First see if the interviewee offers up the option of ‘insufficient or need more information’ as this is what we think should be the response, but we want to see if some users for example find only sensitivity and specificity sufficient and do not identify needing to know impact on downstream patient-important outcomes.*

Yes □ No □ Need More Information □ Why?

|  |
| --- |

**Say**: For the following set of questions I will ask to what extent do you agree with these statements on a 7-point scale. The answer options for each question are as before; Strongly Disagree, Disagree, Somewhat Disagree, I am not sure, Somewhat Agree, Agree, Strongly Agree. Please refer to the GREEN section of the answer sheet now to help you choose the appropriate answers.

***Note to interviewer****: Repeat the answer options for each question (“The answer options again are Strongly Disagree, Disagree, Somewhat Disagree…”) until not necessary.*

1. The purpose of the different prevalence values presented was clear to me:

□ □ □ □ □ □ □

Strongly Disagree Somewhat Neither Agree Somewhat Agree Strongly

Disagree Disagree or Disagree Agree Agree

Why?: ________________________________________________________

_____________________________________________________________

1. The reason for the assigned quality of evidence scores assigned in the SoF table was clear to me:

***Note to interviewer:*** *If interviewee has difficulty answering this question provide cue: “Did you understand why the quality of evidence was rated as high?”*

□ □ □ □ □ □ □

Strongly Disagree Somewhat Neither Agree Somewhat Agree Strongly

Disagree Disagree or Disagree Agree Agree

Why?: ________________________________________________________

_____________________________________________________________

1. The purpose of the footnotes was clear to me:

□ □ □ □ □ □ □

Strongly Disagree Somewhat Neither Agree Somewhat Agree Strongly

Disagree Disagree or Disagree Agree Agree

Why?: ________________________________________________________

_____________________________________________________________

1. **Say**: For the following question the answer options are Yes or No and I will ask you the reason for the answer.

Did you read the content in the footnotes?:

Yes □ No □ Why or Why Not?:

|  |
| --- |

**Say:** For the next 2 questions, refer to the RED section of the answer sheet now and I will ask to what extent do you agree with these statements.

1. The content in the footnotes helped me to better understand the data:

□ □ □ □ □ □ □

Strongly Disagree Somewhat Neither Agree Somewhat Agree Strongly

Disagree Disagree or Disagree Agree Agree

Why?: ________________________________________________________

_____________________________________________________________

1. The content in the comments column helped me to better understand the data:

□ □ □ □ □ □ □

Strongly Disagree Somewhat Neither Agree Somewhat Agree Strongly

Disagree Disagree or Disagree Agree Agree

Why?: ________________________________________________________

_____________________________________________________________

**Say:** For the following 3 questions, refer to the YELLOW section of the answer sheet. For these questions we are simply asking about the extent of your agreement with the statement.

1. Presenting the SoF table as part of a DTA systematic review is a helpful way of reporting the outcomes:

□ □ □ □ □ □ □

Strongly Disagree Somewhat Neither Agree Somewhat Agree Strongly

Disagree Disagree or Disagree Agree Agree

Why?: ________________________________________________________

_____________________________________________________________

1. The density of information presented in this SoF table format is appropriate:

□ □ □ □ □ □ □

Strongly Disagree Somewhat Neither Agree Somewhat Agree Strongly

Disagree Disagree or Disagree Agree Agree

Why?: ________________________________________________________

_____________________________________________________________

1. The information presented in this SoF table format is easy to read:

□ □ □ □ □ □ □

Strongly Disagree Somewhat Neither Agree Somewhat Agree Strongly

Disagree Disagree or Disagree Agree Agree

Why?: ________________________________________________________

_____________________________________________________________

**Say**: We have also proposed an alternative look for this SoF format which I will ask you some questions about. Please refer to the printout for SoF Format 1 – Layout 2 on Page 2.

1. **Ask:** Of the two layouts which do you prefer? Layout 1 where the sensitivity and specificity information is presented similarly as other information in the header in the same font, or Layout 2 where the sensitivity and specificy information is boxed from the rest of the information in the header and the number of participants and studies is also placed in the header instead of in a column?:

Layout 1 □ Layout 2 □ Any comment?:_____________________________

_______________________________________________________________

_______________________________________________________________

**F. Instructions for Evaluation of Components – 20 seconds**

**Say:** In addition to evaluating the SoF table formats overall, we are also interested in gathering detailed feedback on specific parts of the table. For these next questions, we will be focusing on one component of the table.

**G. Quality of Evidence Rating Component – 4 minutes**

**Say**: The component we will evaluate is the Quality of Evidence Rating. Please refer to the printout of SoF Table Component: Quality of Evidence Rating on Page 3. This SoF table uses a different systematic review example with different results, so review the table for about a minute, paying attention specifically to the Quality of the Evidence Column.

**Say**: For the first question I will ask to what extent do you agree with these statements on the 7-point scale. Please refer to the RED section of the answer sheet to help you choose the appropriate answer.

1. The brief reason provided in the table for the quality of evidence rating helps me to better understand the assigned grade.

□ □ □ □ □ □ □

Strongly Disagree Somewhat Neither Agree Somewhat Agree Strongly

Disagree Disagree or Disagree Agree Agree

Why?: ________________________________________________________

_____________________________________________________________

**Say**: For the next question, refer to the YELLOW section of the answer sheet.

1. SoF table users will be more likely to read the footnotes if a brief reason for the quality of evidence rating is provided in the Quality of Evidence Column.

□ □ □ □ □ □ □

Strongly Disagree Somewhat Neither Agree Somewhat Agree Strongly

Disagree Disagree or Disagree Agree Agree

Why?: ________________________________________________________

_____________________________________________________________

**Say**: I will now ask you a few open ended questions about the quality of evidence rating.

1. **Ask:** How detailed should the explanation of the quality of evidence rating in the footnotes be? What information would you like to have presented in the explanation?

|  |
| --- |

1. **Say:** The two footnotes in this table referred to the same reasons for downgrading quality of evidence, but one footnote was more deatiled than the other. Given what you mentioned above, we would like to confirm your preference. Do you prefer a more detailed explanation about the quality of evidence rating, with specific information about the studies, for example a statement explaining that 65% of the studies reported blinding or how wide the confidence intervals were, as is demonstrated in Footnote #1. Or, do you prefer a more minimal explanation for the quality of evidence rating, as is demonstrated in Footnote #2?

Detailed (Footnote 1) □ Minimal (Footnote 2) □ Any comment?:___________

_______________________________________________________________

_______________________________________________________________

1. **Ask:** Would you change anything about the presentation of the brief reason provided in the Quality of Evidence column inside the table? Was this a helpful feature of the table?

|  |
| --- |

1. **Ask:** The rationale for the GRADE quality of evidence rating may not be found anywhere else in a systematic review except in the SoF table footnotes. Should the information be repeated elsewhere, and if so,where?

|  |
| --- |

**H. Review Single Test SoF Format 2 – 7 minutes**

**Say:** We will now return to reviewing alternative formats of the SoF table. Please refer to the printout of SoF Format 2 – Layout 1 on Page 4. Review this format for about 1 minute to familiarize yourself with it. This SoF format uses the same systematc review example as the first format you reviewed. Let me know when you are ready.

**Say**: For the following questions I will ask to what extent do you agree with these statements on the 7-point scale. Please refer to the RED section of the answer sheet to help choose the appropriate answer for the first question.

1. Presenting the sensitivity and specificity test properties as rows in the table helped me to better understand the data:

□ □ □ □ □ □ □

Strongly Disagree Somewhat Neither Agree Somewhat Agree Strongly

Disagree Disagree or Disagree Agree Agree

Why?: ________________________________________________________

_____________________________________________________________

**Say**: For the following 3 questions, refer to the YELLOW section of the answer sheet to help choose the appropriate answer.

1. Presenting the sensitivity and specificity as rows in the table as opposed to inside the header of the table improves accessibility of the systematic review results:

□ □ □ □ □ □ □

Strongly Disagree Somewhat Neither Agree Somewhat Agree Strongly

Disagree Disagree or Disagree Agree Agree

Why?: ________________________________________________________

_____________________________________________________________

1. The density of information presented in this SoF table format is appropriate:

□ □ □ □ □ □ □

Strongly Disagree Somewhat Neither Agree Somewhat Agree Strongly

Disagree Disagree or Disagree Agree Agree

Why?: ________________________________________________________

_____________________________________________________________

1. The information in this SoF table format is easy to read:

□ □ □ □ □ □ □

Strongly Disagree Somewhat Neither Agree Somewhat Agree Strongly

Disagree Disagree or Disagree Agree Agree

Why?: ________________________________________________________

_____________________________________________________________

**Say:** I will now ask a few open ended questions seeking your thoughts about SoF format 2. Remember to think aloud so we can capture your opinion:

1. **Ask**: What is your overall impression of this SoF table format? Are sensitivity and specificity properties necessary in a SoF table at all? Why or why not? Does having this information inside the table help you make a link between the sensitivity and specificity and the test results?

Sens/Spec are Necessary in a SoF □ Sens/Spec are Not Necessary in a SoF □

|  |
| --- |

**Say**: We have also proposed an alternative layout for this SoF format. Please refer to the printout for SoF Format 2 – Layout 2 on Page 5.

1. **Ask:** Consider the presentation order of the columns in SoF Format 2. Do you prefer for the TP, FN, TN, FP test results to be presented on the left side of the table, adjacent to the sensitivity and specificty column, as in Layout 2 (Pg. 5), or do you prefer Layout 1 (Pg. 4) where the test result columns are on the right side of the table adjacent to the comments column? Please compare the two layouts on Pages 4 and 5 to help visualize the difference.

Test Result Columns on Left (Layout 2) □ Columns on Right (Layout 1) □

Any comment?____________________________________________________

_______________________________________________________________

**Say:** I will now ask you about your overall preference between SoF Format 1 that we first reviewed on Page 1 and 2, and SoF Format 2 that we just reviewed now.

1. **Ask:** Do you prefer SoF Format 1 (on Pg. 1 and 2) with sensitivity and specificity information in the header or SoF Format 2 (on Pg. 4 and 5) with sensitivity and specificity as rows inside the table?

SoF Format 1 □ SoF Format 2 □ Any comment?:_______________________

_______________________________________________________________

_______________________________________________________________

**I. Review Single Test SoF Format 3 (SoR Table) – 3 minutes**

**Say:** We will now review a third alternative format of the SoF table. This SoF table is a bit different from the first two formats you have reviewed. Please refer to the printout of SoF Table Format 3 on Page 6. Review this format for about 1 minute. Let me know when you are ready to answer the questions about this format.

**Say:** I will now ask a few open ended questions seeking your thoughts about SoF format 3.

1. **Ask**: What is your overall impression of this SoF table format?

|  |
| --- |

1. **Ask**: Does this SoF table format have specific features or does it present information that you find more helpful to ***understanding the systematic review results*** when compared to the features in the previous two SoF formats? Which features were more helpful?

|  |
| --- |

Is there something missing?

|  |
| --- |

**Say:** I will now ask you about your overall preference between this SoF format, format 3, and the previous two SoF formats, 1 and 2, we reviewed.

1. Ask: Do you prefer SoF format 3 (Pg. 6) over SoF formats 1 (Pg. 1) and 2 (Pg. 4)?

Prefer SoF Format 3 □ Prefer SoF Format 1 or 2 □ Any comment?:___________

_______________________________________________________________

**J. Prevalence Value Component – 5 minutes**

**Say**: We would like to focus on a specific component of the SoF table. The component we will evaluate now is the prevalence values presented in the SoF tables. Please refer to the printout of SoF Table Component: Prevalence – Layout 1 on Page 7. Review the SoF table, noting the example clinical scenario provided, and let me know when you are ready.

**Say**: For the following questions I will ask to what extent you agree with these statements on the 7-point scale. For the first question, please refer to the RED section of the answer sheet to help choose the appropriate answer.

1. The presentation of a clinical scenario is valuable in helping with the interpretation of the different prevalence values:

□ □ □ □ □ □ □

Strongly Disagree Somewhat Neither Agree Somewhat Agree Strongly

Disagree Disagree or Disagree Agree Agree

Why?: ________________________________________________________

_____________________________________________________________

**Say**: For the next question, please refer to the YELLOW section of the answer sheet to help choose the appropriate answer.

1. The presentation of various prevalence estimates is important for decision making as it helps to demonstrate the diagnostic tests accuracy outcomes in different clinical settings

□ □ □ □ □ □ □

Strongly Disagree Somewhat Neither Agree Somewhat Agree Strongly

Disagree Disagree or Disagree Agree Agree

Why?: ________________________________________________________

_____________________________________________________________

**Say**: I will also ask you a few open ended questions about the prevalence component.

1. **Ask:** Do you think that different prevalence estimates should be presented in a SoF table? Why or why not?

|  |
| --- |

1. **Ask:** Ideally, how many estimates should be presented?

|  |
| --- |

1. **Ask:** What do you think should be the source for obtaining the prevalence value estimates, and what should be considered when choosing the prevalence values to present?

|  |
| --- |

1. **Ask:** Do you think that an example clinical scenario should be presented in a SoF table to accompany the prevalence value estimate?

Yes, present clinical scenario □ No, clinical scenario is unnecessary □

If you answered “yes” what should be the source for the clinical scenario that is presented?

|  |
| --- |

**Say**: We have also proposed alternative layouts for presenting the prevalence value estimates and would like to ask you about your preferences. Please refer to the printouts of SoF Table Component: Prevalence – Layout 2 Table 1 on Page 8 and SoF Table Component: Prevalence – Layout 2 Table on Page 9.

1. **Ask:** Do you prefer for the different prevalence value estimates to be presented in a single table, as in Layout 1, or do you prefer the presentation of separate tables for different prevalence values, as in Layout 2?

Layout 1 (Single Table) □ Layout 2 (Separate Tables) □

**Ask:** What is the reason for your preference?:________________________________

_______________________________________________________________

_______________________________________________________________

1. **Ask:** Finally, what is your preference for the labelling of the prevalence value estimate, should it be called:

Prevalence □ Pre-test Probability □ Initial Probability □ Baseline Risk □

Or, Other □ (specify): _________________________________________

Any comment?:_________________________________________________

____________________________________________________________

**[This can be the end of the interview if time only allows for part of the interviw]**

**K. Review Comparative Test SoF Format 1 – 3 minutes**

**Say:** We will now move on to reviewing a SoF table format for comparative diagnostic tests. These SoF table formats present data from DTA systematic reviews in which two tests are compared against a reference standard. Please refer to the printout of Comparative Test SoF Table Format 1 on Page 10. Review this format for about 1 minute. Let me know when you are ready to give us your thoughts by answering the following questions.

1. **Ask:** What is your overall impression of this SoF table format?

|  |
| --- |

**Say**: For the following questions I will ask to what extent do you agree with these statements on the 7-point scale. For the first 3 questions, refer to the RED section of the answer sheet to help choose the appropriate answer.

1. The presentation of the absolute difference helped me to better understand the data:

□ □ □ □ □ □ □

Strongly Disagree Somewhat Neither Agree Somewhat Agree Strongly

Disagree Disagree or Disagree Agree Agree

Why?: ________________________________________________________

_____________________________________________________________

1. The confidence intervals presented in the table are easy to understand:

□ □ □ □ □ □ □

Strongly Disagree Somewhat Neither Agree Somewhat Agree Strongly

Disagree Disagree or Disagree Agree Agree

Why?: ________________________________________________________

_____________________________________________________________

1. Presenting confidence intervals in the SoF table is helpful for decision making about the diagnostic tests:

□ □ □ □ □ □ □

Strongly Disagree Somewhat Neither Agree Somewhat Agree Strongly

Disagree Disagree or Disagree Agree Agree

Why?: ________________________________________________________

_____________________________________________________________

**Say**: For the next 3 questions, refer to the YELLOW section of the answer sheet to help choose the appropriate answer.

1. The SoF table is clear in showing how the diagnostic test accuracy results compare between the two tests:

□ □ □ □ □ □ □

Strongly Disagree Somewhat Neither Agree Somewhat Agree Strongly

Disagree Disagree or Disagree Agree Agree

Why?: ________________________________________________________

_____________________________________________________________

1. The density of information presented in this SoF table format is appropriate:

□ □ □ □ □ □ □

Strongly Disagree Somewhat Neither Agree Somewhat Agree Strongly

Disagree Disagree or Disagree Agree Agree

Why?: ________________________________________________________

_____________________________________________________________

1. The information in this SoF table format is easy to read:

□ □ □ □ □ □ □

Strongly Disagree Somewhat Neither Agree Somewhat Agree Strongly

Disagree Disagree or Disagree Agree Agree

Why?: ________________________________________________________

_____________________________________________________________

**Say:** I will now ask one more open ended question relating to Comparative Test SoF table Format 1.

1. **Ask:** What is your interpretation of the confidence intervals presented in the table? How does this data affect your decision making about the diagnostic tests?

|  |
| --- |

**L. Review Comparative Test SoF Format 2 – 3 Minutes**

**Say:** We will now briefly review an alternative format of the Comparative Test SoF table. Please refer to the printout of Comparative Test SoF Table Format 2 on Page 11. Review the SoF table and let me know when you are ready for the questions about this format.

**Say:** I will now ask you about your preferences between the two Comparative Test SoF formats we have reviewed.

1. **Ask:** Do you prefer Comparative Test SoF Format 1 (on Pg. 10) with sensitivity and specificity information in the header, or Comparative Test SoF Format 2 (on Pg.11) with sensitivity and specificity as rows inside the table?

Comparative Test SoF Format 1 □ Comparative Test SoF Format 2 □ Any comment?:___

_______________________________________________________________

_______________________________________________________________

1. **Ask:** Should confidence intervals also be included for the absolute difference as in Comparative Test SoF Format 2, or should they be left out as in Comparative Test SoF Format 1? Why?

Include (Format 2) □ Exclude (Format 1) □ Why?:_____________________

_______________________________________________________________

_______________________________________________________________

1. **Ask:** Is there any additional information you would like to see presented in a SoF table comparing two tests? Would you change anything about the presentation of Comparative Test SoF Format 1 or 2?

|  |
| --- |

**M. Review Comparative Test SoF Format 3 – 2 Minutes**

**Say:** We will now review a third alternative format of the Comparative Test SoF table. Please refer to the printout of Comparative Test SoF Table Format 3 on Page 12. Review this format for about 1 minute. Let me know when you are ready to answer the questions about this format.

**Say:** I will now ask a few open ended questions seeking your thoughts about SoF format 3.

1. **Ask**: What is your overall impression of this SoF table format?

|  |
| --- |

1. **Ask**: Does this SoF table format have specific features or does it present information that you find more helpful to ***understanding the systematic review results*** and ***decision making about the two diagnostic tests*** when compared to the features in the previous two SoF formats? Which features were more helpful?

|  |
| --- |

**Say:** I will now ask you about your overall preference between this Comparative Test SoF format, format 3, and the previous two Comparative Test SoF formats, 1 and 2, we reviewed.

1. Ask: Do you prefer SoF format 3 (Pg. 12) over SoF formats 1 (Pg. 10) and 2 (Pg. 11)?

Prefer SoF Format 3 □ Prefer SoF Format 1 or 2 □ Any comment?:___________

_______________________________________________________________

_______________________________________________________________

**N. Review Indirect Comparative Test SoF Formats – 5 minutes**

**Say:** There are also instances when we would like to compare two diagnostic tests, but they may not have been evaluated in the same diagnostic test accuracy study. For this, we must draw an indirect comparison using data on the tests from separate studies in different populations. We would like to ask a few questions about your thoughts on the best approach for the indirect comparison of diagnostic tests.

**Say:** Please refer to the printouts of Indirect Comparative Tests: SoF Table 1 – ParaCheck Test and Indirect Comparative Tests: SoF Table 2 – ParaSight Test on Pages 13 and 14. Review the two SoF tables for about 1 minute to grasp the information presented.

**Say:** Now take a look at Indirect Comparative Tests: SoF Table 3 - Combined Tests on Page 15. Review this table for about another minute and let me know when you are ready to answer the questions about this approach.

**Say**: For the following 3 questions I will ask to what extent do you agree with these statements on the 7-point scale. Please refer to the YELLOW section of the answer sheet to help choose the appropriate answer.

1. Using the two separate SoF tables for the individual tests, it is clear which test has better diagnostic test accuracy results.

□ □ □ □ □ □ □

Strongly Disagree Somewhat Neither Agree Somewhat Agree Strongly

Disagree Disagree or Disagree Agree Agree

Why?: ________________________________________________________

_____________________________________________________________

1. Presenting two separate SoF tables for the individual diagnostic tests is sufficient for comparing and decision making about the tests.

□ □ □ □ □ □ □

Strongly Disagree Somewhat Neither Agree Somewhat Agree Strongly

Disagree Disagree or Disagree Agree Agree

Why?: ________________________________________________________

_____________________________________________________________

1. Presenting a combined SoF table with data for both tests and absolute difference in test results is necessary for decision making about the tests.

□ □ □ □ □ □ □

Strongly Disagree Somewhat Neither Agree Somewhat Agree Strongly

Disagree Disagree or Disagree Agree Agree

Why?: ________________________________________________________

_____________________________________________________________

**Say:** I will now ask a few open ended questions seeking your thoughts about the Indirect Comparative Test SoF formats.

1. **Ask:** Do you think that presenting one combined SoF table for the indirect comparison is sufficient for decision making about the two tests? Why or why not? In your opinion, what does the combined SoF table add over the two individual SoF tables for each test, or what is the advantage to having the two individual SoF tables for decision making?

|  |
| --- |

1. **Ask:** In the GRADE approach, in the case of an indirect comparison it is recommended to downgrade the quality of evidence by one level for indirectness of comparison. This is explained in the first footnote in Indirect Comparative Tests: SoF Table 3 - Combined Tests on Page 1. Did you notice this difference between the individual test SoF tables and the Combined Table? How does this affect your opinion on the presentation of individual and a combined SoF table for indirect comparative tests?

Yes, Noticed the Difference □ Do, Did Not Notice Difference □

|  |
| --- |

**Say:** In summary of these questions, I will now confirm your preference for which SoF tables to present when making an indirect comparison between two diagnostic tests.

1. **Ask:** Do you prefer having the individual SoF tables for each test (on Pg. 13 and 14), or only the combined SoF table (on Pg. 15), or all 3 SoF tables available in order to make the indirect comparison between two diagnostic tests?

Individual SoF Formats □ Combined SoF Format Only □ All 3 SoF Formats □

Any comment?:____________________________________________________

_______________________________________________________________

**[Layer 2: (Optional) to assess usefulness of SoF tables for decision making]**

**O. Comments Column Component – 4 minutes**

**Say**: The next component we would like some insight about is the Comments Column. Please refer to the printouts of Comments Column – Example Table 1 on Page 16 and Comments Column – Example Table 2 on Page 17 . Review the SoF tables, noting the information provided in the comments column of each table.

**Say**: I will now ask you a few open ended questions about the Comments Column.

1. **Ask:** As the two tables demonstrate, the contents of the comments column can be very simple, or more detailed, for instance providing some considerations about the perceived downstream consequences of the diagnostic test results. What content do you think should be presented in this column?

|  |
| --- |

1. **Ask:** Diagnostic accuracy studies do not provide direct evidence about patient-important outcomes and one must make deductions about the downstream impact on patient-important outcomes. What do you think should be considered in making the link between diganostic test accuracy results and downstream patient-important outcomes? How do you think this link between the diagnostic test accuracy results and patient outcomes should be presented in a SoF table?

|  |
| --- |

1. **Ask:** Is there any content from the footnotes that you would like to see presented in the comments column instead? Or, vise versa, is there any content from the comments column that should be placed in the footnotes?

|  |
| --- |

**P. Component: Additional Outcomes – 2 minutes**

1. **Ask:**Is there any additional information you would like to see presented in a SoF table to better evaluate diagnostic tests and make decisions? What information would be more helpful?

|  |
| --- |

1. **Say**: Take a brief look at the SoF Table on Page 18, SoF Table Component: Presentation of Outcomes for an example. This table aims to present additional outcomes about a diagnostic test, including inconclusive results, complications of the test, and resource use, to help with decision making. Is additional information like this necessary for decision making, or is DTA sufficient? Why or why not?

Yes, Necessary □ No, DTA is Sufficient □

|  |
| --- |

1. **Ask:** Currently, these types of outcomes are often not reported in diagnostic test accuracy studies. Do you think more effort should be made to evaluate these outcomes in DTA studies? Why or why not?

Yes □ No □

|  |
| --- |

**Q. Conclusion – 2 minutes**

- - 1. **Ask:** Do you have any final comments that you would like to make about the content and presentation of information in SoF tables, keeping in mind that the goal of this interview was to identify problematic areas for users and to determine the usefulness and usability of the tables for presentation of DTA systematic review data?

|  |
| --- |

- - 1. **Say:** We would also like your feedback on how we might have organised this session better. Do you have any suggestions for improving the user testing and this interview?

|  |
| --- |

**Say:** That marks the end of our interview. Thank you very much for your participation, we really appreciate you dedicating your time to this.

Appendix C S1: enhancing the usability and usefulness of summary of findings tables and evidence profiles for decision making about diagnostic tests

**Summary of Findings (SoF) Table Format 1 – Layout 1**

| **Galactomannan ELISA for the diagnosis of invasive aspergillosis** | | | | | | |
| --- | --- | --- | --- | --- | --- | --- |
| **Patients or population:** immunocompromized patients, mostly hematology patients^1^ **Settings:** mainly hematology or cancer departments, mainly inpatients **New Test:** commercial Platelia® sandwich ELISA detecting galactomannan in serum^2^ \| **Cut-off value:** 1.5 ODI  **Reference Test:** composite of EORTC/MSG clinical and histological criteria^3^ **\| Threshold:** Proven or probable invasive aspergillosis  **Pooled Sensitivity:** 0.64 (95% CI 0.50 to 0.77) \| **Pooled Specificity:** 0.95 (95% CI 0.91 to 0.97) | | | | | | |
| **Test Result** | **Number of results**  **per 1000 patients tested**  **(95% CI)** | | | **Number of participants (studies)** | **Quality of the Evidence**  **(GRADE)** | **Comments** |
|  | Assumed numbers with galactomannan ELISA compared to reference test (EORTC/MSG criteria) | | |  |  |  |
|  | **Prevalence 2%^4^** | **Prevalence 12%^4^** | |  |  |  |
| **True positives** | **6** (5 to 8) | | **77** (60 to 92) | 2777 (18) | ⊕⊕⊕⊕  **High^5^** | True positives are important, because patients will be treated with improved survival and spared the invasive diagnostic procedures, however they will suffer the toxicity of the treatment. |
| **False negatives** | **4** (2 to 5) | | **43** (28 to 60) |  |  | False negatives are important, because of missed diagnosis and either will not be treated or treatment will be delayed with uncertain consequences on mortality. |
| **True negatives** | **941** (901 to 960) | | **836** (801 to 854) | 2777 (18) | ⊕⊕⊕⊕  **High^5^** | True negatives are important, because patients will not be treated unnecessarily, will be spared the toxic effects of treatment, and will be reassured. |
| **False positives** | **50** (30 to 89) | | **44** (26 to 79) |  |  | False positives are important, because patients will be treated unnecessarily and many will be exposed to nephrotoxic drugs, also because of a possibly delayed diagnosis and treatment of true cause of symptoms. |
| **CI:** Confidence interval; **ODI:** Optical density index; **EORTC:** European Organization for Research and Treatment of Cancer; **MSG:** Mycoses Study Group | | | | | | |
| Leeflang M, Debets-Ossenkopp Y, Visser C, Scholten R, Hooft L, Bijlmer H, et al. Galactomannan detection for invasive aspergillosis in immunocompromized patients. Cochrane Database of Systematic Reviews. 2008; 4 (Art. No.: CD007394). | | | | | | |
| **Footnotes:**  ^1^ One study that included only patients with HIV/AIDS was excluded, because this patient group and setting differed from the other included patient groups in such a way that authors of the review regarded the study population as not being representative.  ^2^ A diagnostic test for invasive aspergillosis (IA) needs to be not too invasive or a burden to immunocompromised patients. The galactomannan ELISA test on a serum specimen is less burdensome than the current reference standard.  ^3^ A true reference standard is autopsy combined with culture from autopsy specimens; a clinical reference standard is composed of EORTC/MSG clinical and histological criteria.  ^4^ Estimates of prevalence of IA were based on the median and range of values in included studies.  ^5^ Included studies were valid diagnostic accuracy studies in patients with diagnostic uncertainty and direct comparison of test results with an appropriate reference standard. Quality was not downgraded based on the GRADE criteria. | | | | | | |

**SoF Table Format 1 – Layout 2**

| **Galactomannan ELISA for the diagnosis of invasive aspergillosis** | | | | |
| --- | --- | --- | --- | --- |
| **Patients or population:** immunocompromized patients, mostly hematology patients^1^ **Settings:** mainly hematology or cancer departments, mainly inpatients **New Test:** commercial Platelia® sandwich ELISA detecting galactomannan in serum^2^ **\| Cut-off value:** 1.5 ODI  **Reference Test:** composite of EORTC/MSG clinical and histological criteria^3^ **\| Threshold:** Proven or probable invasive aspergillosis   \| **Number of Participants**  **(Studies)** \| 2777 (18) \| **Pooled Sensitivity** \| 0.64 (95% CI 0.50 to 0.77) \| **Pooled Specificity** \| 0.95 (95% CI 0.91 to 0.97) \| \| --- \| --- \| --- \| --- \| --- \| --- \| | | | | |
| **Test Result** | **Number of Results per 1000 patients tested**  **(95% CI)** | | **Quality of the Evidence**  **(GRADE)** | **Comments** |
|  | Assumed numbers with galactomannan ELISA compared to reference test (EORTC/MSG criteria) | |  |  |
|  | **Prevalence 2%^4^** | **Prevalence 12%^4^** |  |  |
| **True positives** | **6** (5 to 8) | **77** (60 to 92) | ⊕⊕⊕⊕  **High^5^** | True positives are important, because patients will be treated with improved survival and spared the invasive diagnostic procedures, however they will suffer the toxicity of the treatment. |
| **False negatives** | **4** (2 to 5) | **43** (28 to 60) |  | False negatives are important, because of missed diagnosis and either will not be treated or treatment will be delayed with uncertain consequences on mortality. |
| **True negatives** | **941** (901 to 960) | **836** (801 to 854) | ⊕⊕⊕⊕  **High^5^** | True negatives are important, because patients will not be treated unnecessarily, will be spared the toxic effects of treatment, and will be reassured. |
| **False positives** | **50** (30 to 89) | **44** (26 to 79) |  | False positives are important, because patients will be treated unnecessarily and many will be exposed to nephrotoxic drugs, also because of a possibly delayed diagnosis and treatment of true cause of symptoms. |
| **CI:** Confidence interval; **ODI:** Optical density index; **EORTC:** European Organization for Research and Treatment of Cancer; **MSG:** Mycoses Study Group | | | | |
| Leeflang M, Debets-Ossenkopp Y, Visser C, Scholten R, Hooft L, Bijlmer H, et al. Galactomannan detection for invasive aspergillosis in immunocompromized patients. Cochrane Database of Systematic Reviews. 2008; 4 (Art. No.: CD007394). | | | | |
| **Footnotes:**  ^1^ One study that included only patients with HIV/AIDS was excluded, because this patient group and setting differed from the other included patient groups in such a way that authors of the review regarded the study population as not being representative.  ^2^ A diagnostic test for invasive aspergillosis (IA) needs to be not too invasive or a burden to immunocompromised patients. The galactomannan ELISA test on a serum specimen is less burdensome than the current reference standard.  ^3^ A true reference standard is autopsy combined with culture from autopsy specimens; a clinical reference standard is composed of EORTC/MSG clinical and histological criteria.  ^4^ Estimates of prevalence of IA were based on the median and range of values in included studies.  ^5^ Included studies were valid diagnostic accuracy studies in patients with diagnostic uncertainty and direct comparison of test results with an appropriate reference standard. Quality was not downgraded based on the GRADE criteria. | | | | |

**SoF Table Component: Quality of Evidence Rating**

| **ParaSight-F compared to microscopy in diagnosis of malaria in outpatients with symptoms suggestive of malaria in P. falciparum endemic areas** | | | | |
| --- | --- | --- | --- | --- |
| **Patients or population:** patients with symptoms suggestive of malaria  **Settings:** ambulatory health facilities in P. falciparum endemic areas **New Test:** ParaSight-F  **Cut-off value:** -  **Reference Test:** microscopy or PCR  **Threshold:** Proven or probable malaria   \| **Number of Participants**  **(Studies)** \| 12,591 (17) \| **Pooled Sensitivity** \| 0.94 (0.90 to 0.97) \| **Pooled Specificity** \| 0.95 (0.90 to 0.97) \| \| --- \| --- \| --- \| --- \| --- \| --- \| | | | | |
| **Test Result** | **Number of results**  **per 1000 patients tested**  **(95% CI)** | | **Quality of the Evidence**  **(GRADE)** | **Comments** |
|  | **Prevalence 10%** | **Prevalence 30%** |  |  |
| **True positives** | **94** (90 to 97) | **283** (269 to 290) | ⊕⊕⊕O  **Moderate**  Due to Risk of Bias^1,2^ | True positives are important, because patients will be treated with improved survival. |
| **False negatives** | **6** (3 to 10) | **17** (10 to 31) |  | False negatives are important, because of missed diagnosis and either will not be treated or treatment will be delayed with uncertain consequences on morbidity and mortality. |
| **True negatives** | **851** (813 to 872) | **662** (632 to 678) | ⊕⊕⊕O  **Moderate**  Due to Risk of Bias,^1,2^ | True negatives are important, because patients will not be treated unnecessarily and will be reassured. |
| **False positives** | **50** (28 to 87) | **39** (22 to 68) |  | False positives are important, because patients will be treated unnecessarily, also because of a possibly delayed diagnosis and treatment of true cause of symptoms. |
| **CI:** Confidence interval; **PCR:** Polymerase chain reaction | | | | |
| Abba K, Deeks JJ, Olliaro PL, Naing CM, Jackson SM, Takwoingi Y, Donegan S, Garner P. Rapid diagnostic tests for diagnosing uncomplicated P. falciparum malaria in endemic countries. Cochrane Database of Systematic Reviews 2011; 7 (Art. No.: CD008122). | | | | |
| **Footnotes:**  ^1^ Downgraded for risk of bias. Downgraded based on the assessment of all Type 1 studies included in the review together (only 25% reported an adequate reference standard, only half of the included studies were explicit about patient recruitment involving a consecutive or random series of patients, blinding of the index and reference tests was reported in 65% and 70%, respectively).  ^2^ Downgraded for risk of bias due to lack of adequate reference standard, blinding, and lack of information about patient recruitment in studies. | | | | |

**SoF Table Format 2 – Layout 1**

| **Galactomannan ELISA for the diagnosis of invasive aspergillosis** | | | | | | |  | |  |
| --- | --- | --- | --- | --- | --- | --- | --- | --- | --- |
| **Patients or population:** immunocompromized patients, mostly hematology patients^1^ **Settings:** mainly hematology or cancer departments, mainly inpatients **New Test:** commercial Platelia® sandwich ELISA detecting galactomannan in serum^2^**. Cut-off value:** 1.5 ODI  **Reference Test:** composite of EORTC/MSG clinical and histological criteria^3^  **Threshold:** Proven or probable invasive aspergillosis | | | | | | | |  |  |
| **Test Property**  **(95% CI)** | **Number of participants (studies)** | **Quality**  **of the Evidence**  **(GRADE)** | | **Test Result** | **Number of results**  **per 1000 patients tested**  **(95% CI)**  Assumed numbers with galactomannan ELISA compared to reference test (EORTC/MSG criteria) | | | | **Comments** |
|  |  |  |  |  | **Prevalence 2%^4^** | **Prevalence 12%^4^** | | |  |
| **Sensitivity**  0.64  (0.50 to 0.77) | 2777  (18) | ⊕⊕⊕⊕  **High^5^** | True positives | | **6** (5 to 8) | **77** (60 to 92) | | | True positives are important, because patients will be treated with improved survival and spared the invasive diagnostic procedures, however they will suffer the toxicity of the treatment. |
|  |  |  | False negatives | | **4** (2 to 5) | **43** (28 to 60) | | | False negatives are important, because of missed diagnosis and either will not be treated or treatment will be delayed with uncertain consequences on mortality. |
| **Specificity**  0.95  (0.91 to 0.97) | 2777  (18) | ⊕⊕⊕⊕  **High^5^** | True negatives | | **941** (901 to 960) | **836** (801 to 854) | | | True negatives are important, because patients will not be treated unnecessarily, will be spared the toxic effects of treatment, and will be reassured. |
|  |  |  | False positives | | **50** (30 to 89) | **44** (26 to 79) | | | False positives are important, because patients will be treated unnecessarily and many will be exposed to nephrotoxic drugs, also because of a possibly delayed diagnosis and treatment of true cause of symptoms. |
| **CI:** Confidence interval; **ODI:** Optical density index; **EORTC:** European Organization for Research and Treatment of Cancer; **MSG:** Mycoses Study Group | | | | | | | | | |
| Leeflang M, Debets-Ossenkopp Y, Visser C, Scholten R, Hooft L, Bijlmer H, et al. Galactomannan detection for invasive aspergillosis in immunocompromized patients. Cochrane Database of Systematic Reviews. 2008; 4 (Art. No.: CD007394). | | | | | | | | | |
| **Footnotes:**  ^1^ One study that included only patients with HIV/AIDS was excluded, because this patient group and setting differed from the other included patient groups in such a way that authors of the review regarded the study population as not being representative.  ^2^ A diagnostic test for invasive aspergillosis (IA) needs to be not too invasive or a burden to immunocompromised patients. The galactomannan ELISA test on a serum specimen is less burdensome than the current reference standard.  ^3^ A true reference standard is autopsy combined with culture from autopsy specimens; a clinical reference standard is composed of EORTC/MSG clinical and histological criteria.  ^4^ Estimates of prevalence of IA were based on the median and range of values in included studies.  ^5^ Included studies were valid diagnostic accuracy studies in patients with diagnostic uncertainty and direct comparison of test results with an appropriate reference standard. Quality was not downgraded based on the GRADE criteria. | | | | | | | | | |

**SoF Table Format 2 – Layout 2**

| **Galactomannan ELISA for the diagnosis of invasive aspergillosis** | | | | | | | | |
| --- | --- | --- | --- | --- | --- | --- | --- | --- |
| **Patients or population:** immunocompromized patients, mostly hematology patients^1^ **Settings:** mainly hematology or cancer departments, mainly inpatients **New Test:** commercial Platelia® sandwich ELISA detecting galactomannan in serum^2^**. Cut-off value:** 1.5 ODI  **Reference Test:** composite of EORTC/MSG clinical and histological criteria^3^  **Threshold:** Proven or probable invasive aspergillosis | | | | | |  |  |  |
| **Test Property**  **(95% CI)** | **Test Result** | **Number of results**  **per 1000 patients tested**  **(95% CI)**  Assumed numbers with galactomannan ELISA compared to reference test (EORTC/MSG criteria) | | **Number of participants (studies)** | **Quality**  **of the Evidence**  **(GRADE)** | | **Comments** | |
|  |  | **Prevalence 2%^4^** | **Prevalence 12%^4^** |  |  |  |  |  |
| Sensitivity  0.64  (0.50 to 0.77) | True positives | 6 (5 to 8) | 77 (60 to 92) | 2777  (18) | ⊕⊕⊕⊕  **High^5^** | | True positives are important, because patients will be treated with improved survival and spared the invasive diagnostic procedures, however they will suffer the toxicity of the treatment. | |
|  | False negatives | 4 (2 to 5) | 43 (28 to 60) |  |  |  | False negatives are important, because of missed diagnosis and either will not be treated or treatment will be delayed with uncertain consequences on mortality. | |
| Specificity  0.95  (0.91 to 0.97) | True negatives | 941 (901 to 960) | 836 (801 to 854) | 2777  (18) | ⊕⊕⊕⊕  **High^5^** | | True negatives are important, because patients will not be treated unnecessarily, will be spared the toxic effects of treatment, and will be reassured. | |
|  | False positives | 50 (30 to 89) | 44 (26 to 79) |  |  |  | False positives are important, because patients will be treated unnecessarily and many will be exposed to nephrotoxic drugs, also because of a possibly delayed diagnosis and treatment of true cause of symptoms. | |
| **CI:** Confidence interval; **ODI:** Optical density index; **EORTC:** European Organization for Research and Treatment of Cancer; **MSG:** Mycoses Study Group | | | | | | | | |
| Leeflang M, Debets-Ossenkopp Y, Visser C, Scholten R, Hooft L, Bijlmer H, et al. Galactomannan detection for invasive aspergillosis in immunocompromized patients. Cochrane Database of Systematic Reviews. 2008; 4 (Art. No.: CD007394). | | | | | | | | |
| **Footnotes:**  ^1^ One study that included only patients with HIV/AIDS was excluded, because this patient group and setting differed from the other included patient groups in such a way that authors of the review regarded the study population as not being representative.  ^2^ A diagnostic test for invasive aspergillosis (IA) needs to be not too invasive or a burden to immunocompromised patients. The galactomannan ELISA test on a serum specimen is less burdensome than the current reference standard.  ^3^ A true reference standard is autopsy combined with culture from autopsy specimens; a clinical reference standard is composed of EORTC/MSG clinical and histological criteria.  ^4^ Estimates of prevalence of IA were based on the median and range of values in included studies.  ^5^ Included studies were valid diagnostic accuracy studies in patients with diagnostic uncertainty and direct comparison of test results with an appropriate reference standard. Quality was not downgraded based on the GRADE criteria. | | | | | | | | |

**SoF Table Format 3**

| **What is the diagnostic accuracy of the galactomannan ELISA for invasive aspergillosis for different cut-off values?** | | | | |  |
| --- | --- | --- | --- | --- | --- |
| **Patients/population:** immunocompromized patients, mostly hematology patients.  **Prior testing:** varied, mostly underlying disease or symptoms (fever, neutropenia)  **Settings:** mainly hematology or cancer departments, mainly inpatients.  **Index test:** a sandwich ELISA for galactomannan, an *Aspergillus* antigen.  **Importance:** depends on the time-gain the test may give.  **Reference standard:** gold standard is autopsy, but that is nearly never done; so in most studies the reference standard is composed of clinical and microbiological criteria  **Studies:** patient series or case-control studies, not using an in-house test and not excluding possibly infected patients. Studies (n = 29) had to report cut-off values that were used; 0.5 ODI, 1.0 ODI, or 1.5 ODI. Each study can be present in more than one subgroup. The results presented in this table are for the 1.5 ODI subgroup only. | | | | |  |
| **Subgroup** | **Effect**  **(95% CI)** | **Number of participants (studies)** | **Prevalence**  **(median, range)** | **What do these results mean?** |  |
| Cut-off 1.5 ODI | **Sensitivity 0.64**  (0.50 to 0.77)  Specificity 0.95  (0.91 to 0.97) | 2777  (18) | **Median 12.4%**  (0.8% to 44%) | With a prevalence of 12%^1^, 12 out of 100 patients will develop IA. Of these, 4 will be missed by the Platelia test (36% of 12), but will be tested again. Of the 88 patients without IA, only 4 will be unnecessarily referred for CT scanning.  *In children (1 study, 17 participants), the sensitivity was higher (100%) and the specificity was lower (50%).* |  |
| **CI:** Confidence interval; **ODI:** Optical density index; | | | | |  |
| Leeflang M, Debets-Ossenkopp Y, Visser C, Scholten R, Hooft L, Bijlmer H, et al. Galactomannan detection for invasive aspergillosis in immunocompromized patients. Cochrane Database of Systematic Reviews. 2008; 4 (Art. No.: CD007394). | | | | |  |
| **Footnotes:**  ^1^ Prevalence over all 28 studies (children-studies excluded): 4501 participants; median 12% (range 0.8% to 44%). | | | | |  |

**SoF Table Component: Prevalence – Layout 1**

| **Galactomannan ELISA for the diagnosis of invasive aspergillosis** | | | | |
| --- | --- | --- | --- | --- |
| **Patients or population:** immunocompromized patients, mostly hematology patients. **Settings:** mainly hematology or cancer departments, mainly inpatients **New Test:** commercial Platelia® sandwich ELISA detecting galactomannan in serum^1^**. Cut-off value:** 1.5 ODI  **Reference Test:** composite of EORTC/MSG clinical and histological criteria^2^**. Threshold:** Proven or probable invasive aspergillosis   \| **Number of Participants**  **(Studies)** \| 2777 (18) \| **Pooled Sensitivity** \| 0.64 (95% CI 0.50 to 0.77) \| **Pooled Specificity** \| 0.95 (95% CI 0.91 to 0.97) \| \| --- \| --- \| --- \| --- \| --- \| --- \| | | | | |
| **Test Result** | **Number of results per 1000 patients tested**  **(95% CI)^3^** | | **Quality of the Evidence**  **(GRADE)** | **Comments** |
|  | **Prevalence 2%^4^** | **Prevalence 12%^4^** |  |  |
|  | **Exaple Clinical Scenario:** Adults undergoing transplant, no neutropenic patients. | **Example Clinical Scenario:** Adults with hematological disorders that were neutropenic, underwent chemotherapy, had persistent fever despite antibiotics, acute graft versus host disease, or received corticosteroids. |  |  |
| **True positives** | **6** (5 to 8) | **77** (60 to 92) | ⊕⊕⊕⊕  **High^5^** | Patients will be correctly classified and treated; mortality and disability will be reduced, quality of life will be improved. |
| **False negatives** | **4** (2 to 5) | **43** (28 to 60) |  | Patients may receive unnecessary treatment and suffer its adverse effects; true cause of symptoms may be missed and correct diagnosis will be delayed. |
|  | | | | |
| **True negatives** | **941** (901 to 960) | **836** (801 to 854) | ⊕⊕⊕⊕  **High^5^** | Patients will be reassured that they do not have stroke and will undergo investigation for other causes of symptoms and likely will be treated accordingly. |
| **False positives** | **50** (30 to 89) | **44** (26 to 79) |  | Patients will be falsely reassured that they do not have stroke, unnecessary further testing for other causes of symptoms may be performed and the potentially beneficial treatment will be delayed. |
| **CI:** Confidence interval; **ODI:** Optical density index; **EORTC:** European Organization for Research and Treatment of Cancer; **MSG:** Mycoses Study Group | | | | |
| Leeflang M, Debets-Ossenkopp Y, Visser C, Scholten R, Hooft L, Bijlmer H, et al. Galactomannan detection for invasive aspergillosis in immunocompromized patients. Cochrane Database of Systematic Reviews. 2008; 4 (Art. No.: CD007394). | | | | |
| **Footnotes:**  ^1^ A diagnostic test for invasive aspergillosis (IA) needs to be not too invasive or a burden to immunocompromised patients. The galactomannan ELISA test on a serum specimen is less burdensome than the current reference standard.  ^2^ A true reference standard is autopsy combined with culture from autopsy specimens; a clinical reference standard is composed of EORTC/MSG clinical and histological criteria.  ^3^ Assumed numbers with galactomannan ELISA compared to reference test (EORTC/MSG criteria).  ^4^ Estimates of prevalence of IA were based on the median and range of values in included studies.  ^5^ Included studies were valid diagnostic accuracy studies in patients with diagnostic uncertainty and direct comparison of test results with an appropriate reference standard. Quality was not downgraded based on the GRADE criteria. | | | | |

**SoF Table Component: Prevalence – Layout 2 Table 1**

| **Galactomannan ELISA for the diagnosis of invasive aspergillosis** | | | | |
| --- | --- | --- | --- | --- |
| **Patients or population:** immunocompromized patients, mostly hematology patients **Settings:** mainly hematology or cancer departments, mainly inpatients **New Test:** commercial Platelia® sandwich ELISA detecting galactomannan in serum^1^  **Cut-off value:** 1.5 ODI  **Reference Test:** composite of EORTC/MSG clinical and histological criteria^2^  **Threshold:** Proven or probable invasive aspergillosis   \| **Prevalence:** \| **2%^3^** \| **Pooled Sensitivity:** \| 0.64 (95% CI 0.50 to 0.77) \| \| --- \| --- \| --- \| --- \| \| **Example Clinical Scenario:** \| Adults undergoing transplant, no neutropenic patients. \| **Pooled Specificity:** \| 0.95 (95% CI 0.91 to 0.97) \| | | | | |
| **Test Result** | **Number of results**  **per 1000 patients tested**  **(95% CI)^4^** | **Number of participants (studies)** | **Quality of the Evidence**  **(GRADE)** | **Comments** |
| **True positives** | **6** (5 to 8) | 2777 (18) | ⊕⊕⊕⊕  **High^5^** | True positives are important, because patients will be treated with improved survival and spared the invasive diagnostic procedures, however they will suffer the toxicity of the treatment. |
| **False negatives** | **4** (2 to 5) |  |  | False negatives are important, because of missed diagnosis and either will not be treated or treatment will be delayed with uncertain consequences on mortality. |
|  |  |  |  |  |
| **True negatives** | **941** (901 to 960) | 2777 (18) | ⊕⊕⊕⊕  **High^5^** | True negatives are important, because patients will not be treated unnecessarily, will be spared the toxic effects of treatment, and will be reassured. |
| **False positives** | **50** (30 to 89) |  |  | False positives are important, because patients will be treated unnecessarily and many will be exposed to nephrotoxic drugs, also because of a possibly delayed diagnosis and treatment of true cause of symptoms. |
| **CI:** Confidence interval; **ODI:** Optical density index; **EORTC:** European Organization for Research and Treatment of Cancer; **MSG:** Mycoses Study Group | | | | |
| Leeflang M, Debets-Ossenkopp Y, Visser C, Scholten R, Hooft L, Bijlmer H, et al. Galactomannan detection for invasive aspergillosis in immunocompromized patients. Cochrane Database of Systematic Reviews. 2008; 4 (Art. No.: CD007394). | | | | |
| **Footnotes:**  ^1^ A diagnostic test for invasive aspergillosis (IA) needs to be not too invasive or a burden to immunocompromised patients. The galactomannan ELISA test on a serum specimen is less burdensome than the current reference standard.  ^2^ A true reference standard is autopsy combined with culture from autopsy specimens; a clinical reference standard is composed of EORTC/MSG clinical and histological criteria.  ^3^ Estimate of prevalence of IA was based on the median and range of values in included studies.  ^4^ Assumed numbers with galactomannan ELISA compared to reference test (EORTC/MSG criteria).  ^5^ Included studies were valid diagnostic accuracy studies in patients with diagnostic uncertainty and direct comparison of test results with an appropriate reference standard. Quality was not downgraded based on the GRADE criteria. | | | | |

**SoF Table Component: Prevalence – Layout 2 Table 2**

| **Galactomannan ELISA for the diagnosis of invasive aspergillosis** | | | | |
| --- | --- | --- | --- | --- |
| **Patients or population:** immunocompromized patients, mostly hematology patients **Settings:** mainly hematology or cancer departments, mainly inpatients **New Test:** commercial Platelia® sandwich ELISA detecting galactomannan in serum^1^  **Cut-off value:** 1.5 ODI  **Reference Test:** composite of EORTC/MSG clinical and histological criteria^2^  **Threshold:** Proven or probable invasive aspergillosis   \| **Prevalence:** \| **12%^3^** \| **Pooled Sensitivity:** \| 0.64 (95% CI 0.50 to 0.77) \| \| --- \| --- \| --- \| --- \| \| **Example Clinical Scenario:** \| Adults with hematological disorders that were neutropenic, underwent chemotherapy, had persistent fever despite antibiotics, acute graft versus host disease, or received corticosteroids. \| **Pooled Specificity:** \| 0.95 (95% CI 0.91 to 0.97) \| | | | | |
| **Test Result** | **Number of results**  **per 1000 patients tested**  **(95% CI)^4^** | **Number of participants (studies)** | **Quality of the Evidence**  **(GRADE)** | **Comments** |
| **True positives** | **6** (5 to 8) | 2777 (18) | ⊕⊕⊕⊕  **High^5^** | True positives are important, because patients will be treated with improved survival and spared the invasive diagnostic procedures, however they will suffer the toxicity of the treatment. |
| **False negatives** | **4** (2 to 5) |  |  | False negatives are important, because of missed diagnosis and either will not be treated or treatment will be delayed with uncertain consequences on mortality. |
|  |  |  |  |  |
| **True negatives** | **836** (801 to 854) | 2777 (18) | ⊕⊕⊕⊕  **High^5^** | True negatives are important, because patients will not be treated unnecessarily, will be spared the toxic effects of treatment, and will be reassured. |
| **False positives** | **44** (26 to 79) |  |  | False positives are important, because patients will be treated unnecessarily and many will be exposed to nephrotoxic drugs, also because of a possibly delayed diagnosis and treatment of true cause of symptoms. |
| **CI:** Confidence interval; **ODI:** Optical density index; **EORTC:** European Organization for Research and Treatment of Cancer; **MSG:** Mycoses Study Group | | | | |
| Leeflang M, Debets-Ossenkopp Y, Visser C, Scholten R, Hooft L, Bijlmer H, et al. Galactomannan detection for invasive aspergillosis in immunocompromized patients. Cochrane Database of Systematic Reviews. 2008; 4 (Art. No.: CD007394). | | | | |
| **Footnotes:**  ^1^ A diagnostic test for invasive aspergillosis (IA) needs to be not too invasive or a burden to immunocompromised patients. The galactomannan ELISA test on a serum specimen is less burdensome than the current reference standard.  ^2^ A true reference standard is autopsy combined with culture from autopsy specimens; a clinical reference standard is composed of EORTC/MSG clinical and histological criteria.  ^3^ Estimates of prevalence of IA were based on the median and range of values in included studies.  ^4^ Assumed numbers with galactomannan ELISA compared to reference test (EORTC/MSG criteria).  ^5^ Included studies were valid diagnostic accuracy studies in patients with diagnostic uncertainty and direct comparison of test results with an appropriate reference standard. Quality was not downgraded based on the GRADE criteria. | | | | |

**Comparative Test SoF Table Format 1**

| **Magnetic resonance imaging compared to computed tomography for detection of acute vascular lesions in patients presenting with acute stroke symptoms** | | | | | | |
| --- | --- | --- | --- | --- | --- | --- |
| **Patients or population:** adult patients suspected of acute stroke within 12 hours of the onset of symptoms. **Settings:** hospital emergency departments  **New Test:** diffusion-weighted magnetic resonance imaging **Cut-off value:** –  **Comparison Test:** Non-contrast computed tomography **Cut-off value:** –  **Reference Test:** a combination of clinical and imaging information supported by clinical or imaging follow up (CT or MRI)   \| **Number of Participants**  **(Studies)** \| 226  (7) \| **Pooled Sensitivity CT** \| 0.39 (95% CI 0.16 to 0.69) \| **Pooled Sensitivity MRI** \| 0.99 (95% CI 0.23 to 1.00) \| \| --- \| --- \| --- \| --- \| --- \| --- \| \| **Pooled Specificity CT** \| 1.00 (95% CI 0.94 to 1.00) \| **Pooled Specificity MRI** \| 0.92 (95% CI 0.83 to 0.97) \| | | | | | | |
| **Test Result** | **Number of results per 1000 patients tested**  **(95% CI)** | | | | **Quality**  **of the Evidence**  **(GRADE)** | **Comments** |
|  | **Prevalence 50%^1^** | | **Prevalence 70%^2^** | |  |  |
|  | **CT** | **MRI** | **CT** | **MRI** |  |  |
| **True positives** (TP)^3^ | **195**  (80 to 345) | **495**  (115 to 500) | **273**  (112 to 483) | **693**  (161 to 700) | ⊕OOO  **Very Low^4,5^** | Patients will be correctly classified and treated; mortality and disability will be reduced, quality of life will be improved. |
| TP absolute difference | **300 more** | | **420 more** | |  |  |
| **False negatives** (FN) | **305**  (155 to 420) | **5**  (0 to 385) | **427**  (217 to 588) | **7**  (0 to 539) |  | Patients may receive unnecessary treatment and suffer its adverse effects; true cause of symptoms may be missed and correct diagnosis will be delayed. |
| FN absolute difference | **300 fewer** | | **420 fewer** | |  |  |
|  | | | | | | |
| **True negatives** (TN) | **500**  (470 to 500) | **460**  (415 to 485) | **300**  (282 to 300) | **276**  (249 to 291) | ⊕OOO  **Very Low^4,5^** | Patients will be reassured that they do not have stroke and will undergo investigation for other causes of symptoms and likely will be treated accordingly. |
| TN absolute difference | **40 fewer** | | **24 fewer** | |  |  |
| **False positives** (FP)^3^ | **0**  (0 to 30) | **40**  (15 to 85) | **0**  (0 to 18) | **24**  (9 to 51) |  | Patients will be falsely reassured that they do not have stroke, unnecessary further testing for other causes of symptoms may be performed and the potentially beneficial treatment will be delayed. |
| FP absolute difference | **40 more** | | **24 more** | |  |  |
| **CI:** Confidence interval; **CT**: Non-contrast computed tomography; **MRI**: diffusion-weighted magnetic resonance imaging | | | | | | |
| Brazelli M, Sandercock P, Chappell F, Celani M, Righetti E, Arestis N, et al. Magnetic resonance imaging versus computed tomography for detection of acute vascular lesions in patients presenting with stroke symptoms. Cochrane Database of Systematic Reviews. 2009; 4 (Art. No.: CD007424). | | | | | | |
| **Footnotes:**  ^1^ Prevalence of 50% was assumed to be the average prevalence in a representative population (this assumed prevalence should ideally be based on observational studies done in the target population)  ^2^ Prevalence of 70% was estimated based on the mean prevalence of ischemic stroke in the included studies.  ^3^ In some studies patients whose symptoms lasted less than 24 hours but who had evidence of an ischemic lesion on imaging were counted as having had strokes and hence analyzed as true positive cases. In other studies, however, patients with symptom duration less than 24 hours and an ischemic lesion on imaging were analyzed as being false positive cases.  ^4^ Serious risk of bias, because of unblinded reference standard results and clinical information available that otherwise would not be available; serious indirectness (not representative population); serious imprecision (only 226 patients and very wide confidence intervals); serious inconsistency (sensitivity of CT varied very widely)  ^5^ Serious risk of bias, because of unblinded reference standard results and clinical information available that otherwise would not be available; serious indirectness (not representative population); serious imprecision (only 226 patients and very wide confidence intervals) | | | | | | |

**Comparative Test SoF Table Format 2**

| **Magnetic resonance imaging compared to computed tomography for detection of acute vascular lesions in patients presenting with acute stroke symptoms** | | | | | | | | | | |
| --- | --- | --- | --- | --- | --- | --- | --- | --- | --- | --- |
| **Patients or population:** adult patients suspected of acute stroke within 12 hours of the onset of symptoms  **Settings:** hospital emergency departments  **New Test:** diffusion-weighted magnetic resonance imaging **Cut-off value:** –  **Comparison Test:** Non-contrast computed tomography **Cut-off value:** –  **Reference Test:** a combination of clinical and imaging information supported by clinical or imaging follow up (CT or MRI) | | | | | | | | | | |
| **Test Property** | **Summary Estimate**  **(95% CI)** | | **Test Result** | **Number of results**  **per 1000 patients tested (95% CI)** | | | | **Number of participants  (studies)** | **Quality of the Evidence**  **(GRADE)** | **Comments** |
|  |  |  |  | **Prevalence 50%^1^** | | **Prevalence 70%^2^** | |  |  |  |
|  | **CT** | **MRI** |  | **CT** | **MRI** | **CT** | **MRI** |  |  |  |
| **Sensitivity** | **0.39**  (0.16 to 0.69) | **0.99**  (0.23 to 1.00) | **True positives** (TP)^3^ | **195**  (80 to 345) | **495**  (115 to 500) | **273**  (112 to 483) | **693**  (161 to 700) | 226  (7) | ⊕OOO  **Very Low^4,5^** | Patients will be correctly classified and treated; mortality and disability will be reduced, quality of life will be improved. |
|  |  |  | TP absolute difference | **300 more**  (230 fewer to 420 more) | | **420 more**  (322 fewer to 588 more) | |  |  |  |
|  |  |  | **False negatives** (FN) | **305**  (155 to 420) | **5**  (0 to 385) | **427**  (217 to 588) | **7**  (0 to 539) |  |  | Patients may receive unnecessary treatment and suffer its adverse effects; true cause of symptoms may be missed and correct diagnosis will be delayed. |
|  |  |  | FN absolute difference | **300 fewer**  (230 more to 420 fewer) | | **420 fewer**  (322 more to 588 fewer) | |  |  |  |
|  | | |  | | | | | | | |
| **Specificity** | **1.00**  (0.94 to 1.00) | **0.92**  (0.83 to 0.97) | **True negatives** (TN) | **500**  (470 to 500) | **460**  (415 to 485) | **300**  (282 to 300) | **276**  (249 to 291) | 226  (7) | ⊕OOO  **Very Low^4,5^** | Patients will be reassured that they do not have stroke and will undergo investigation for other causes of symptoms and likely will be treated accordingly. |
|  |  |  | TN absolute difference | **40 fewer**  (15 more to 85 fewer) | | **24 fewer**  (9 more to 51 fewer) | |  |  |  |
|  |  |  | **False positives** (FP)^3^ | **0**  (0 to 30) | **40**  (15 to 85) | **0**  (0 to 18) | **24**  (9 to 51) |  |  | Patients will be falsely reassured that they do not have stroke, unnecessary further testing for other causes of symptoms may be performed and the potentially beneficial treatment will be delayed. |
|  |  |  | FP absolute difference | **40 more**  (15 fewer to 85 more) | | **24 more**  (9 fewer to 51 more) | |  |  |  |
| **CI:** Confidence interval; **CT**: Non-contrast computed tomography; **MRI**: diffusion-weighted magnetic resonance imaging | | | | | | | | | | |
| Brazelli M, Sandercock P, Chappell F, Celani M, Righetti E, Arestis N, et al. Magnetic resonance imaging versus computed tomography for detection of acute vascular lesions in patients presenting with stroke symptoms. Cochrane Database of Systematic Reviews. 2009; 4 (Art. No.: CD007424). | | | | | | | | | | |
| **Footnotes:**  ^1^ Prevalence of 50% was assumed to be the average prevalence in a representative population (this assumed prevalence should ideally be based on observational studies done in the target population)  ^2^ Prevalence of 70% was estimated based on the mean prevalence of ischemic stroke in the included studies.  ^3^ In some studies patients whose symptoms lasted less than 24 hours but who had evidence of an ischemic lesion on imaging were counted as having had strokes and hence analyzed as true positive cases. In other studies, however, patients with symptom duration less than 24 hours and an ischemic lesion on imaging were analyzed as being false positive cases.  ^4^ Serious risk of bias, because of unblinded reference standard results and clinical information available that otherwise would not be available; serious indirectness (not representative population); serious imprecision (only 226 patients and very wide confidence intervals); serious inconsistency (sensitivity of CT varied very widely)  ^5^ Serious risk of bias, because of unblinded reference standard results and clinical information available that otherwise would not be available; serious indirectness (not representative population); serious imprecision (only 226 patients and very wide confidence intervals) | | | | | | | | | | |

**Comparative Test SoF Table Format 3**

| **Comparison of diffusion-weighted magnetic resonance imaging with conventional computer tomography for the early detection of ischaemic brain lesions in patients suspected of stroke** | | | | | | |
| --- | --- | --- | --- | --- | --- | --- |
| **Patient population:** adults suspected of acute stroke. **Setting:** hospital departments  **Geographical location:** studies were conducted in Europe (3 studies), the USA (3 studies), and in Australia (1 study)  **Index test:** diffusion-weighted magnetic resonance imaging (MRI) performed within 12 hours of stroke onset  **Alternative test:** computer tomography (CT) performed within 12 hours of stroke onset  **Reference standard:** clinical assessment and imaging follow up  **Included studies:** 7 comparative studies that evaluated MRI and CT in the same patients  **Total number of patients assessed:** 226 | | | | | | |
| **Limitations of included studies**   - Limited number of included studies (7 studies); small sample sizes; presence of incorporation bias - MRI and CT were evaluated in highly selected patient samples (patients with high probability of stroke), which therefore are not representative of the typical population of patients presenting with ’suspected acute stroke’ to an emergency department (poor generalisability of results) - The stroke vascular territory was not reported in the majority of included studies although it is likely that they enrolled patients with typical anterior circulation stroke - Only a minority of the studied patients had severe strokes (in whom MRI might be contraindicated) - The high proportion of mild strokes and reclassification of TIA cases with a positive MRI lesion as strokes might have inflated the DWI sensitivity estimate - In most of the studies stroke mimics were not included - In all but one study CT was performed before MRI (reducing the sensitivity of CT to detect ischaemia) | | | | | | |
| **CT Results** | | | **MRI Results** | | **Summary Effect**  **(95% CI)** | |
| **TP** | **73** | **TP** | | **147** | **CT sensitivity** | 0.39 (0.16 to 0.69) |
| **FP** | **0** | **FP** | | **5** | **CT specificity** | 1.00 (0.94 to 1.00) |
| **TN** | **88** | **TN** | | **14** | **MRI sensitivity** | 0.99 (0.23 to 1.00) |
| **FN** | **65** | **FN** | | **60** | **MRI specificity** | 0.92 (0.83 to 0.97) |
| **Total** | **226** | **Total** | | **226** |  |  |
| **Conclusions and comments**  In the small cohort of included studies, MRI is more sensitive than CT - but not more specific - for the early detection of ischaemic stroke. The small amount of data and the presence of methodological biases preclude any reliable calculation - from the sensitivity and specificity estimates of CT and MRI - of a positive or negative stroke diagnosis at different rates of stroke prevalence. | | | | | | |
| **Applicability of tests in clinical practice**  None of the studies addressed practicality. CT is known to be quicker to perform and more readily available in most emergency care settings than magnetic resonance imaging (MRI). MRI is contraindicated in patients with pacemakers and some metal implants. In acutely ill stroke patients it may be difficult to monitor the patient’s condition while being MR scanned (and this increases the risk of any respiratory difficulty or cardiovascular compromise that develops during the scan which passes undetected and may have adverse effects for the patient). If the patient is confused or restless as a result of the stroke, the patient may not be able to co-operate for the longer scan times of MRI. | | | | | | |
| **Costs**  None of the studies included a cost-effectiveness evaluation. MRI is known to be more expensive than CT. | | | | | | |
| **CI:** Confidence interval; **CT**: computed tomography; **MRI**: diffusion-weighted magnetic resonance imaging; **TP**: true positive; **FP**: false positive; **TN**: true negative **FN**: false negative | | | | | | |
| Brazelli M, Sandercock P, Chappell F, Celani M, Righetti E, Arestis N, et al. Magnetic resonance imaging versus computed tomography for detection of acute vascular lesions in patients presenting with stroke symptoms. Cochrane Database of Systematic Reviews. 2009; 4 (Art. No.: CD007424). | | | | | | |

**Indirect Comparative Tests: SoF Table 1 – ParaCheck Test**

| **Paracheck-Pf compared to microscopy in diagnosis of malaria in outpatients with symptoms suggestive of malaria in P. falciparum endemic areas** | | | | |
| --- | --- | --- | --- | --- |
| **Patients or population:** patients with symptoms suggestive of malaria  **Settings:** ambulatory health facilities in P. falciparum endemic areas **New Test:** Paracheck-Pf  **Cut-off value:** -  **Reference Test:** microscopy or PCR  **Threshold:** Proven or probable malaria   \| **Number of Participants**  **(Studies)** \| 22,319 (27) \| **Pooled Sensitivity** \| 0.93 (0.90 to 0.96) \| **Pooled Specificity** \| 0.96 (0.93 to 0.98) \| \| --- \| --- \| --- \| --- \| --- \| --- \| | | | | |
| **Test Result** | **Number of results**  **per 1000 patients tested**  **(95% CI)** | | **Quality of the Evidence**  **(GRADE)** | **Comments** |
|  | **Prevalence 10%** | **Prevalence 30%** |  |  |
| **True positives** | **93** (90 to 96) | **280** (269 to 287) | ⊕⊕⊕O  **Moderate^1^** | True positives are important, because patients will be treated with improved survival. |
| **False negatives** | **7** (4 to 10) | **20** (13 to 31) |  | False negatives are important, because of missed diagnosis and either will not be treated or treatment will be delayed with uncertain consequences on morbidity and mortality. |
| **True negatives** | **861** (834 to 878) | **670** (649 to 683) | ⊕⊕⊕O  **Moderate^1^** | True negatives are important, because patients will not be treated unnecessarily and will be reassured. |
| **False positives** | **39** (23 to 66) | **30** (18 to 51) |  | False positives are important, because patients will be treated unnecessarily, also because of a possibly delayed diagnosis and treatment of true cause of symptoms. |
| **CI:** Confidence interval; **PCR:** Polymerase chain reaction | | | | |
| Abba K, Deeks JJ, Olliaro PL, Naing CM, Jackson SM, Takwoingi Y, Donegan S, Garner P. Rapid diagnostic tests for diagnosing uncomplicated P. falciparum malaria in endemic countries. Cochrane Database of Systematic Reviews 2011; 7 (Art. No.: CD008122). | | | | |
| **Footnotes:**  ^1^ Downgraded for risk of bias. Downgraded based on the assessment of all Type 1 studies included in the review together (only 25% reported an adequate reference standard, only half of the included studies were explicit about patient recruitment involving a consecutive or random series of patients, blinding of the index and reference tests was reported in 65% and 70%, respectively). | | | | |

**Indirect Comparative Tests: SoF Table 2 – ParaSight Test**

| **ParaSight-F compared to microscopy in diagnosis of malaria in outpatients with symptoms suggestive of malaria in P. falciparum endemic areas** | | | | |
| --- | --- | --- | --- | --- |
| **Patients or population:** patients with symptoms suggestive of malaria  **Settings:** ambulatory health facilities in P. falciparum endemic areas **New Test:** ParaSight-F  **Cut-off value:** -  **Reference Test:** microscopy or PCR  **Threshold:** Proven or probable malaria   \| **Number of Participants**  **(Studies)** \| 12,591 (17) \| **Pooled Sensitivity** \| 0.94 (0.90 to 0.97) \| **Pooled Specificity** \| 0.95 (0.90 to 0.97) \| \| --- \| --- \| --- \| --- \| --- \| --- \| | | | | |
| **Test Result** | **Number of results**  **per 1000 patients tested**  **(95% CI)** | | **Quality of the Evidence**  **(GRADE)** | **Comments** |
|  | **Prevalence 10%** | **Prevalence 30%** |  |  |
| **True positives** | **94** (90 to 97) | **283** (269 to 290) | ⊕⊕⊕O  **Moderate^1,2^** | True positives are important, because patients will be treated with improved survival. |
| **False negatives** | **6** (3 to 10) | **17** (10 to 31) |  | False negatives are important, because of missed diagnosis and either will not be treated or treatment will be delayed with uncertain consequences on morbidity and mortality. |
| **True negatives** | **851** (813 to 872) | **662** (632 to 678) | ⊕⊕⊕O  **Moderate^1,2^** | True negatives are important, because patients will not be treated unnecessarily and will be reassured. |
| **False positives** | **50** (28 to 87) | **39** (22 to 68) |  | False positives are important, because patients will be treated unnecessarily, also because of a possibly delayed diagnosis and treatment of true cause of symptoms. |
| **CI:** Confidence interval; **PCR:** Polymerase chain reaction | | | | |
| Abba K, Deeks JJ, Olliaro PL, Naing CM, Jackson SM, Takwoingi Y, Donegan S, Garner P. Rapid diagnostic tests for diagnosing uncomplicated P. falciparum malaria in endemic countries. Cochrane Database of Systematic Reviews 2011; 7 (Art. No.: CD008122). | | | | |
| **Footnotes:**  ^1^ Downgraded for risk of bias. Downgraded based on the assessment of all Type 1 studies included in the review together (only 25% reported an adequate reference standard, only half of the included studies were explicit about patient recruitment involving a consecutive or random series of patients, blinding of the index and reference tests was reported in 65% and 70%, respectively).  ^2^ Low for medium-risk (30% initial probability of malaria) population because of imprecision. | | | | |

**Indirect Comparative Tests: SoF Table 3 - Combined Tests**

| **Pracheck-Pf compared to ParaSight-F in diagnosis of malaria in outpatients with symptoms suggestive of malaria in P. falciparum endemic areas** | | | | | | |
| --- | --- | --- | --- | --- | --- | --- |
| **Patients or population:** patients with symptoms suggestive of malaria  **Settings:** ambulatory health facilities in P. falciparum endemic areas  **Index Test 1:** Paracheck-Pf **Cut-off value:** –  **Index Test 2:** ParaSight-F **Cut-off value:** –  **Reference Test:** microscopy or PCR  **Threshold:** Proven or probable malaria   \| **Number of participants (studies) ParaCheck** \| 22,319 (27) \| **Pooled Sensitivity ParaCheck** \| 0.93 (0.90 to 0.96) \| **Pooled Specificity ParaCheck** \| 0.96 (0.93 to 0.98) \| \| --- \| --- \| --- \| --- \| --- \| --- \| \| **Number of participants (studies) ParaSight** \| 12,591 (17) \| **Pooled Sensitivity ParaSight** \| 0.94 (0.90 to 0.97) \| **Pooled Specificity ParaSight** \| 0.95 (0.90 to 0.97) \| | | | | | | |
| **Test Result** | **Number of results per 1000 patients tested**  **(95% CI)** | | | | **Quality of the Evidence**  **(GRADE)^1^** | **Comments** |
|  | **Prevalence 10%** | | **Prevalence 30%** | |  |  |
|  | **ParaCheck** | **ParaSight** | **ParaCheck** | **ParaSight** |  |  |
| **True positives** (TP) | **93**  (90 to 96) | **94**  (90 to 97) | **280**  (269 to 287) | **283**  (269 to 290) | ⊕⊕OO  **Low^1, 2^** | True positives are important, because patients will be treated with improved survival. |
| TP absolute difference | **1 more** | | **3 more** | |  |  |
| **False negatives** (FN) | **7**  (4 to 10) | **6**  (3 to 10) | **20**  (13 to 31) | **17**  (10 to 31) |  | False negatives are important, because of missed diagnosis and either will not be treated or treatment will be delayed with uncertain consequences on morbidity and mortality. |
| FN absolute difference | **1 fewer** | | **3 fewer** | |  |  |
| **True negatives** (TN) | **861**  (834 to 878) | **851**  (813 to 872) | **670**  (649 to 683) | **662**  (632 to 678) | ⊕⊕OO  **Low^1, 2^** | True negatives are important, because patients will not be treated unnecessarily and will be reassured. |
| TN absolute difference | **10 fewer** | | **8 fewer** | |  |  |
| **False positives** (FP) | **39**  (23 to 66) | **50**  (28 to 87) | **30**  (18 to 51) | **39**  (22 to 68) |  | False positives are important, because patients will be treated unnecessarily, also because of a possibly delayed diagnosis and treatment of true cause of symptoms. |
| FP absolute difference | **11 more** | | **9 more** | |  |  |
| **CI:** Confidence interval; **PCR:** Polymerase chain reaction | | | | | | |
| Abba K, Deeks JJ, Olliaro PL, Naing CM, Jackson SM, Takwoingi Y, Donegan S, Garner P. Rapid diagnostic tests for diagnosing uncomplicated P. falciparum malaria in endemic countries. Cochrane Database of Systematic Reviews 2011; 7 (Art. No.: CD008122). | | | | | | |
| **Footnotes:**  ^1^ For an indirect comparison of 2 index tests we suggest that the score for each domain of QoE be determined as the lower of the scores for that domain for each of index tests compared against a reference standard. We suggest that the overall QoE for an indirect comparison of 2 index tests be further downgraded by one level for indirectness of comparison.  ^2^ Downgraded based on the assessment of all Type 1 studies included in the review together (only 25% reported an adequate reference standard, only half of the included studies were explicit about patient recruitment involving a consecutive or random series of patients, blinding of the index and reference tests was reported in 65% and 70%, respectively). | | | | | | |

**SoF Table Component: Comments Column – Example Table 1**

| **Galactomannan ELISA for the diagnosis of invasive aspergillosis** | | | | |
| --- | --- | --- | --- | --- |
| **Patients or population:** immunocompromized patients, mostly hematology patients^1^. **Settings:** mainly hematology or cancer departments, mainly inpatients **New Test:** commercial Platelia® sandwich ELISA detecting galactomannan in serum^2^**. Cut-off value:** 1.5 ODI  **Reference Test:** composite of EORTC/MSG clinical and histological criteria^3^  **Threshold:** Proven or probable invasive aspergillosis   \| **Number of Participants**  **(Studies)** \| 2777 (18) \| **Pooled Sensitivity** \| 0.64 (95% CI 0.50 to 0.77) \| **Pooled Specificity** \| 0.95 (95% CI 0.91 to 0.97) \| \| --- \| --- \| --- \| --- \| --- \| --- \| | | | | |
| **Test Result** | **Number of results**  **per 1000 patients tested**  **(95% CI)** | | **Quality of the Evidence**  **(GRADE)** | **Comments** |
|  | Assumed numbers with galactomannan ELISA compared to reference test (EORTC/MSG criteria) | |  |  |
|  | **Prevalence 2%^4^** | **Prevalence 12%^4^** |  |  |
| **True positives** | **6** (5 to 8) | **77** (60 to 92) | ⊕⊕⊕⊕  **High^5^** | With a prevalence of 12%, 120 out of 1000 patients will develop IA. Of these, 77 will be correctly classified by the Platelia test as having IA (64% of 120) and treated with improved survival. |
| **False negatives** | **4** (2 to 5) | **43** (28 to 60) |  | With a prevalence of 12%, 120 out of 1000 patients will develop IA. Of these, 43 will be incorrectly classified by the Platelia test as not having IA (36% of 120). Patients will not be treated or treatment will be delayed with uncertain consequences on mortality. |
| **True negatives** | **941** (901 to 960) | **836** (801 to 854) | ⊕⊕⊕⊕  **High^5^** | With a prevalence of 12%, 880 out of 1000 patients will not develop IA. Of these, 836 will be correctly classified by the Platelia test (95% of 880) as not having IA. Patients will not be treated unnecessarily, and will be spared the toxic effects of treatment. |
| **False positives** | **50** (30 to 89) | **44** (26 to 79) |  | With a prevalence of 12%, 880 out of 1000 patients will not develop IA. Of these, 44 will be incorrectly classified by the Platelia test (5% of 880) as having IA. Patients will be treated unnecessarily and may be exposed to nephrotoxic drugs, there will be delayed diagnosis and treatment of true cause of symptoms. |
| **CI:** Confidence interval; **ODI:** Optical density index; **EORTC:** European Organization for Research and Treatment of Cancer; **MSG:** Mycoses Study Group | | | | |
| Leeflang M, Debets-Ossenkopp Y, Visser C, Scholten R, Hooft L, Bijlmer H, et al. Galactomannan detection for invasive aspergillosis in immunocompromized patients. Cochrane Database of Systematic Reviews. 2008; 4 (Art. No.: CD007394). | | | | |
| **Footnotes:**  ^1^ One study that included only patients with HIV/AIDS was excluded, because this patient group and setting differed from the other included patient groups in such a way that authors of the review regarded the study population as not being representative.  ^2^ A diagnostic test for invasive aspergillosis (IA) needs to be not too invasive or a burden to immunocompromised patients. The galactomannan ELISA test on a serum specimen is less burdensome than the current reference standard.  ^3^ A true reference standard is autopsy combined with culture from autopsy specimens; a clinical reference standard is composed of EORTC/MSG clinical and histological criteria.  ^4^ Estimates of prevalence of IA were based on the median and range of values in included studies.  ^5^ Included studies were valid diagnostic accuracy studies in patients with diagnostic uncertainty and direct comparison of test results with an appropriate reference standard. Quality was not downgraded based on the GRADE criteria. | | | | |

**SoF Table Component: Comments Column – Example Table 2**

| **Galactomannan ELISA for the diagnosis of invasive aspergillosis** | | | | |
| --- | --- | --- | --- | --- |
| **Patients or population:** immunocompromized patients, mostly hematology patients^1^ **Settings:** mainly hematology or cancer departments, mainly inpatients **New Test:** commercial Platelia® sandwich ELISA detecting galactomannan in serum^2^  **Cut-off value:** 1.5 ODI  **Reference Test:** composite of EORTC/MSG clinical and histological criteria^3^  **Threshold:** Proven or probable invasive aspergillosis   \| **Number of Participants**  **(Studies)** \| 2777 (18) \| **Pooled Sensitivity** \| 0.64 (95% CI 0.50 to 0.77) \| **Pooled Specificity** \| 0.95 (95% CI 0.91 to 0.97) \| \| --- \| --- \| --- \| --- \| --- \| --- \| | | | | |
| **Test Result** | **Number of results**  **per 1000 patients tested**  **(95% CI)** | | **Quality of the Evidence**  **(GRADE)** | **Comments** |
|  | Assumed numbers with galactomannan ELISA compared to reference test (EORTC/MSG criteria) | |  |  |
|  | **Prevalence 2%^4^** | **Prevalence 12%^4^** |  |  |
| **True positives** | **6** (5 to 8) | **77** (60 to 92) | ⊕⊕⊕⊕  **High^5^** | Detected by the test and referred for treatment. |
| **False negatives** | **4** (2 to 5) | **43** (28 to 60) |  | Not detected by the test, with a delay in treatment. |
| **True negatives** | **941** (901 to 960) | **836** (801 to 854) | ⊕⊕⊕⊕  **High^5^** | No referral, patient reassurance. |
| **False positives** | **50** (30 to 89) | **44** (26 to 79) |  | Unnecessary referral for follow-up testing and/or unnecessary treatment. |
| **CI:** Confidence interval; **ODI:** Optical density index; **EORTC:** European Organization for Research and Treatment of Cancer; **MSG:** Mycoses Study Group | | | | |
| Leeflang M, Debets-Ossenkopp Y, Visser C, Scholten R, Hooft L, Bijlmer H, et al. Galactomannan detection for invasive aspergillosis in immunocompromized patients. Cochrane Database of Systematic Reviews. 2008; 4 (Art. No.: CD007394). | | | | |
| **Footnotes:**  ^1^ One study that included only patients with HIV/AIDS was excluded, because this patient group and setting differed from the other included patient groups in such a way that authors of the review regarded the study population as not being representative.  ^2^ A diagnostic test for invasive aspergillosis (IA) needs to be not too invasive or a burden to immunocompromised patients. The galactomannan ELISA test on a serum specimen is less burdensome than the current reference standard.  ^3^ A true reference standard is autopsy combined with culture from autopsy specimens; a clinical reference standard is composed of EORTC/MSG clinical and histological criteria.  ^4^ Estimates of prevalence of IA were based on the median and range of values in included studies.  ^5^ Included studies were valid diagnostic accuracy studies in patients with diagnostic uncertainty and direct comparison of test results with an appropriate reference standard. Quality was not downgraded based on the GRADE criteria. | | | | |

**SoF Table Component: Presentation of Outcomes**

| **Paracheck-Pf compared to microscopy in diagnosis of malaria in outpatients with symptoms suggestive of malaria in P. falciparum endemic areas** | | | | | |
| --- | --- | --- | --- | --- | --- |
| **Patients or population:** patients with symptoms suggestive of malaria. **Settings:** ambulatory health facilities in P. falciparum endemic areas **New Test:** Paracheck-Pf**. Cut-off value:** -  **Reference Test:** microscopy or PCR**. Threshold:** Proven or probable malaria   \| **Pooled Sensitivity** \| 0.93 (0.90 to 0.96) \| **Pooled Specificity** \| 0.96 (0.93 to 0.98) \| \| --- \| --- \| --- \| --- \| | | | | | |
| **Test Result** | **Number of results**  **per 1000 patients tested**  **(95% CI)** | | **Number of participants (studies)** | **Quality of the Evidence**  **(GRADE)** | **Comments** |
|  | **Prevalence 10%** | **Prevalence 30%** |  |  |  |
| **True positives** | **93** (90 to 96) | **280** (269 to 287) | 22,319 (27) | ⊕⊕⊕O  **Moderate^1^** | True positives are important, because patients will be treated with improved survival. |
| **False negatives** | **7** (4 to 10) | **20** (13 to 31) |  |  | False negatives are important, because of missed diagnosis and either will not be treated or treatment will be delayed with uncertain consequences on morbidity and mortality. |
| **True negatives** | **861** (834 to 878) | **670** (649 to 683) | 22,319 (27) | ⊕⊕⊕O  **Moderate^1^** | True negatives are important, because patients will not be treated unnecessarily and will be reassured. |
| **False positives** | **39** (23 to 66) | **30** (18 to 51) |  |  | False positives are important, because patients will be treated unnecessarily, also because of a possibly delayed diagnosis and treatment of true cause of symptoms. |
| **Outcome** | | |  |  |  |
| **Inconclusive Results** | **140 per 1000** (14%) in 1 study;  **10 per 1000** (1%) to **50 per 1000** (5%) in 15 studies;  **Not reported** in 11 studies. | | 11,238 (16) | ⊕OOO  **Very Low^2^** | Uninterpretable, intermediate, or indeterminate test results. They are important because they generate anxiety for the patient, uncertainty as to how to proceed, and likely repeat testing. |
| **Complications** | **Not reported** | | - | - | Complications of the Paracheck-Pf test are unlikely and usually related to blood sampling as it is a noninvasive test. |
| **Resource Use** | **US $0.62 per test**  **Other resource use reported** | | (4) | ⊕⊕⊕O  **Moderate^3^** | The cost of the test is sufficiently low that its routine use for confirmation of P. falciparum malaria is likely to be cost-effective. Other resource use including staffing and facility costs were not reported. |
| **CI:** Confidence interval; **PCR:** Polymerase chain reaction | | | | | |
| Abba K, Deeks JJ, Olliaro PL, Naing CM, Jackson SM, Takwoingi Y, Donegan S, Garner P. Rapid diagnostic tests for diagnosing uncomplicated P. falciparum malaria in endemic countries. Cochrane Database of Systematic Reviews 2011; 7 (Art. No.: CD008122). | | | | | |
| **Footnotes:**  ^1^ Downgraded for risk of bias. Downgraded based on the assessment of all Type 1 studies included in the review together (only 25% reported an adequate reference standard, only half of the included studies were explicit about patient recruitment involving a consecutive or random series of patients, blinding of the index and reference tests was reported in 65% and 70%, respectively).  ^2^ Inconclusive results are inconsistent as they were reported to range from 1% to 14% in different studies.  ^3^ Indirectness of resource use data as cost of test may vary in different settings that those reported in the studies. | | | | | |

Appendix C S1 evidence profile with individual dta outcomes

**Galactomannan ELISA for the diagnosis of invasive aspergillosis**

**Question: Should Galactomannan ELISA be used for the diagnosis of invasive aspergillosis?**

**Authors of the profile**: Nancy Santesso, Jan Brozek, Holger Schünemann

**Population:** immunocompromized patients, mostly hematology patients [1]

**Setting:** mainly hematology or cancer departments, mainly inpatients

**New Test:** commercial Platelia® sandwich ELISA detecting galactomannan in serum [2]. **Cut-off value:** 1.5 ODI

**Reference Test:** composite of EORTC/MSG clinical and histological criteria [3]

**Threshold:** Proven or probable invasive aspergillosis

**Bibliography:** Leeflang et al. Galactomannan detection for invasive aspergillosis in immunocompromized patients. Cochrane Database of Systematic Reviews 2008, Issue 4. Art. No.: CD007394

| **Number of Participants**  **(studies)** | **Study design** | **Risk of bias** | **Indirectness** | **Inconsistency** | **Imprecision** | **Publication**  **bias** | **Effect (number per 1000 patients tested)** | | | **Quality of**  **the evidence**  **(GRADE)** | |  | |
| --- | --- | --- | --- | --- | --- | --- | --- | --- | --- | --- | --- | --- | --- |
|  |  |  |  |  |  |  | **Prevalence**  **1% [4]** | **Prevalence**  **12% [4]** | **Prevalence**  **44% [4]** |  |  |  |  |
| **True positives (Patients correctly classified as having invasive aspergillosis) [6]** | | | | | | | | | | | | | |
| 2777 (18 studies) | cohort, case-control | no | no | no | no | unlikely | **6** (5 to 8) | **77**  (60 to 92) | **282**  (220 to 339) | HIGH |  | | |
| **True negatives (Patients correctly classified as not having invasive aspergillosis) [7]** | | | | | | | | | | | | | |
| 2777 (18 studies) | cohort, case-control | no | no | no | no | unlikely | **941**  (901 to 960) | **836**  (801 to 854) | **532**  (510 to 543) | HIGH | |  |  |
| **False positives (Patients incorrectly classified as having invasive aspergillosis) [8]** | | | | | | | | | | | | | |
| 2777 (18 studies) | cohort, case-control | no | serious [9] | no | no | unlikely | **50**  (89 to 30) | **44**  (79 to 26) | **28**  (50 to 17) | MODERATE | |  |  |
| **False negatives (Patients incorrectly classified as not having the disease) [10]** | | | | | | | | | | | | | |
| 2777 (18 studies) | cohort, case-control | no | serious [11] | no | no | unlikely | **4**  (5 to 2) | **43**  (60 to 28) | **158** (220 to 101) | MODERATE | |  |  |

**Footnotes:**

[1] One study that included only patients with HIV/AIDS was excluded, because this patient group and setting differed from the other included patient groups in such a way that authors of the review regarded the study population as not being representative.

[2] A diagnostic test for invasive aspergillosis (IA) needs to be not too invasive or a burden to immunocompromised patients. The galactomannan ELISA test on a serum specimen is less burdensome that a current reference standard.

[3] A true reference standard is autopsy combined with culture from autopsy specimens; a clinical reference standard is composed of EORTC/MSG clinical and histological criteria.

[4] Estimates of prevalence of IA were based on the median and range of values in included studies.

[5] GRADE recommends classifying outcomes on a 9 point scale: 1-3 is not important; 4-6 is important; and, 7-9 is critical to a decision.

[6] True positives are important because patients will be treated with improved survival and spared the invasive diagnostic procedures, however they will suffer the toxicity of the treatment.

[7] True negatives are important, because patients will not be treated unnecessarily, will be spared the toxic effects of treatment, and will be reassured.

[8] False positives are important, because patients will be treated unnecessarily and many will be exposed to nephrotoxic drugs, also because of a possibly delayed diagnosis and treatment of true cause of symptoms.

[9] There is uncertainty to what extent a delayed diagnosis and treatment of true cause of symptoms would impact on patient-important outcomes.

[10] False negatives important, because of missed diagnosis and either will not be treated with 60–90% mortality rate or treatment will be delayed with uncertain consequences on mortality.

[11] There is some uncertainty to what extent a delayed diagnosis and treatment would impact mortality and other outcomes.

[12] Inconclusive results were not reported. Most likely they would result in repeating the index test and therefore increased cost.

[13] Complications and resource use were not reported.

**Summary of findings table with individual DTA data**

| **Galactomannan ELISA for the diagnosis of invasive aspergillosis** | | | | |
| --- | --- | --- | --- | --- |
| **Patients or population:** immunocompromized patients, mostly hematology patients. **Settings:** mainly hematology or cancer departments, mainly inpatients **New Test:** commercial Platelia® sandwich ELISA detecting galactomannan in serum^1^**. Cut-off value:** 1.5 ODI  **Reference Test:** composite of EORTC/MSG clinical and histological criteria^2^  **Threshold:** Proven or probable invasive aspergillosis   \| **Number of Participants**  **(Studies)** \| 2777 (18) \| **Pooled Sensitivity** \| 0.64 (95% CI 0.50 to 0.77) \| **Pooled Specificity** \| 0.95 (95% CI 0.91 to 0.97) \| \| --- \| --- \| --- \| --- \| --- \| --- \| | | | | |
| **Test Result** | **Number of results per 1000 patients tested**  **(95% CI)^3^** | | **Quality of the Evidence**  **(GRADE)** | **Comments** |
|  | **Prevalence 2%^4^** | **Prevalence 12%^4^** |  |  |
|  | **Exaple Clinical Scenario:** Adults undergoing transplant, no neutropenic patients. | **Example Clinical Scenario:** Adults with hematological disorders that were neutropenic, underwent chemotherapy, had persistent fever despite antibiotics, acute graft versus host disease, or received corticosteroids. |  |  |
| **True positives** | **6** (5 to 8) | **77** (60 to 92) | ⊕⊕⊕⊕  **High^5^** | Patients will be correctly classified and treated; mortality and disability will be reduced, quality of life will be improved. |
| **False negatives** | **4** (2 to 5) | **43** (28 to 60) |  | Patients may receive unnecessary treatment and suffer its adverse effects; true cause of symptoms may be missed and correct diagnosis will be delayed. |
|  | | | | |
| **True negatives** | **941** (901 to 960) | **836** (801 to 854) | ⊕⊕⊕⊕  **High^5^** | Patients will be reassured that they do not have stroke and will undergo investigation for other causes of symptoms and likely will be treated accordingly. |
| **False positives** | **50** (30 to 89) | **44** (26 to 79) |  | Patients will be falsely reassured that they do not have stroke, unnecessary further testing for other causes of symptoms may be performed and the potentially beneficial treatment will be delayed. |
| **CI:** Confidence interval; **ODI:** Optical density index; **EORTC:** European Organization for Research and Treatment of Cancer; **MSG:** Mycoses Study Group | | | | |
| Leeflang M, Debets-Ossenkopp Y, Visser C, Scholten R, Hooft L, Bijlmer H, et al. Galactomannan detection for invasive aspergillosis in immunocompromized patients. Cochrane Database of Systematic Reviews. 2008; 4 (Art. No.: CD007394). | | | | |
| **Footnotes:**  ^1^ A diagnostic test for invasive aspergillosis (IA) needs to be not too invasive or a burden to immunocompromised patients. The galactomannan ELISA test on a serum specimen is less burdensome than the current reference standard.  ^2^ A true reference standard is autopsy combined with culture from autopsy specimens; a clinical reference standard is composed of EORTC/MSG clinical and histological criteria.  ^3^ Assumed numbers with galactomannan ELISA compared to reference test (EORTC/MSG criteria).  ^4^ Estimates of prevalence of IA were based on the median and range of values in included studies.  ^5^ Included studies were valid diagnostic accuracy studies in patients with diagnostic uncertainty and direct comparison of test results with an appropriate reference standard. Quality was not downgraded based on the GRADE criteria. | | | | |

Appendix D S1

**SoF Table – 3 Prevalence estimates in rows (sens and spec in table)**

| **Galactomannan ELISA for the diagnosis of invasive aspergillosis** | | | | | | | | | | |  |
| --- | --- | --- | --- | --- | --- | --- | --- | --- | --- | --- | --- |
| **Patients or population:** immunocompromized patients, mostly hematology patients^1^ **Settings:** mainly hematology or cancer departments, mainly inpatients **New Test:** commercial Platelia® sandwich ELISA detecting galactomannan in serum^2^**.**  **Cut-off value:** 1.5 ODI  **Reference Test:** composite of EORTC/MSG clinical and histological criteria^3^**.**  **Threshold:** Proven or probable invasive aspergillosis | | | | | | | | | | |  |
| **Test Property** | | **Summary Estimate (95% CI)** | | **Number of participants (studies)** | **Quality**  **of the Evidence**  **(GRADE)** | | **Test Result** | | **Illustrative Comparative Numbers per 1000 patients tested**  **(95% CI)^4^**  Assumed numbers with galactomannan ELISA compared to reference test (EORTC/MSG criteria) | | **Comments** |
| **Sensitivity** | | 0.64  (0.50 to 0.77) | | 2777  (18) |  | | **True positives**  (Patients correctly classified as having invasive aspergillosis) | | **Prevalence 1%** | | True positives are important, because patients will be treated with improved survival and spared the invasive diagnostic procedures, however they will suffer the toxicity of the treatment. |
|  |  |  |  |  |  |  |  |  | **6** (5 to 8) | |  |
|  |  |  |  |  | ⊕⊕⊕⊕  **High** | |  |  | **Prevalence 12%** | |  |
|  |  |  |  |  |  |  |  |  | **77** (60 to 92) | |  |
|  |  |  |  |  |  |  |  |  | **Prevalence 44%** | |  |
|  |  |  |  |  |  |  |  |  | **282** (220 to 339) | |  |
|  |  |  |  |  |  |  | **False negatives**  (Patients incorrectly classified as not having invasive aspergillosis) | | **Prevalence 1%** | | False negatives are important, because of missed diagnosis and either will not be treated or treatment will be delayed with uncertain consequences on mortality. |
|  |  |  |  |  |  |  |  |  | **4** (2 to 5) | |  |
|  |  |  |  |  |  |  |  |  | **Prevalence 12%** | |  |
|  |  |  |  |  |  |  |  |  | **43** (28 to 60) | |  |
|  |  |  |  |  |  |  |  |  | **Prevalence 44%** | |  |
|  |  |  |  |  |  |  |  |  | **158** (101 to 220) | |  |
|  | | | | | | | | | | | |
| **Specificity** | 0.95 (0.91 to 0.97) | | 2777  (18) | | ⊕⊕⊕⊕  **High** | **True negatives**  (Patients correctly classified as not having invasive aspergillosis) | | **Prevalence 1%** | | True negatives are important, because patients will not be treated unnecessarily, will be spared the toxic effects of treatment, and will be reassured. | |
|  |  |  |  |  |  |  |  | **941** (901 to 960) | |  |  |
|  |  |  |  |  |  |  |  | **Prevalence 12%** | |  |  |
|  |  |  |  |  |  |  |  | **836** (801 to 854) | |  |  |
|  |  |  |  |  |  |  |  | **Prevalence 44%** | |  |  |
|  |  |  |  |  |  |  |  | **532** (510 to 543) | |  |  |
|  |  |  |  |  |  | **False positives**  (Patients incorrectly classified as having invasive aspergillosis) | | **Prevalence 1%** | | False positives are important, because patients will be treated unnecessarily and many will be exposed to nephrotoxic drugs, also because of a possibly delayed diagnosis and treatment of true cause of symptoms. | |
|  |  |  |  |  |  |  |  | **50** (30 to 89) | |  |  |
|  |  |  |  |  |  |  |  | **Prevalence 12%** | |  |  |
|  |  |  |  |  |  |  |  | **44** (26 to 79) | |  |  |
|  |  |  |  |  |  |  |  | **Prevalence 44%** | |  |  |
|  |  |  |  |  |  |  |  | **28** (17 to 50) | |  |  |
| **CI:** Confidence interval; **ODI:** Optical density index; **EORTC:** European Organization for Research and Treatment of Cancer; **MSG:** Mycoses Study Group | | | | | | | | | | | |
| Leeflang M, Debets-Ossenkopp Y, Visser C, Scholten R, Hooft L, Bijlmer H, et al. Galactomannan detection for invasive aspergillosis in immunocompromized patients. Cochrane Database of Systematic Reviews. 2008; 4 (Art. No.: CD007394). | | | | | | | | | | | |
| **Footnotes:**  ^1^ One study that included only patients with HIV/AIDS was excluded, because this patient group and setting differed from the other included patient groups in such a way that authors of the review regarded the study population as not being representative.  ^2^ A diagnostic test for invasive aspergillosis (IA) needs to be not too invasive or a burden to immunocompromised patients. The galactomannan ELISA test on a serum specimen is less burdensome than the current reference standard.  ^3^ A true reference standard is autopsy combined with culture from autopsy specimens; a clinical reference standard is composed of EORTC/MSG clinical and histological criteria.  ^4^ Estimates of prevalence of IA were based on the median and range of values in included studies. | | | | | | | | | | | |

**SoF Table - 3 Prevalence estimates in rows (sens and spec in header)**

| **Galactomannan ELISA for the diagnosis of invasive aspergillosis** | | | | |
| --- | --- | --- | --- | --- |
| **Patients or population:** immunocompromized patients, mostly hematology patients^1^ **Settings:** mainly hematology or cancer departments, mainly inpatients **New Test:** commercial Platelia® sandwich ELISA detecting galactomannan in serum^2^  **Cut-off value:** 1.5 ODI  **Reference Test:** composite of EORTC/MSG clinical and histological criteria^3^  **Threshold:** Proven or probable invasive aspergillosis  **Pooled Sensitivity:** 0.64 (95% CI 0.50 to 0.77), **Pooled Specificity:** 0.95 (95% CI 0.91 to 0.97) | | | | |
| **Test Result** | **Illustrative Comparative Numbers per 1000 patients tested**  **(95% CI)^4^** | **Number of participants (studies)** | **Quality of the Evidence**  **(GRADE)** | **Comments** |
|  | Assumed numbers with galactomannan ELISA compared to reference test (EORTC/MSG criteria) |  |  |  |
| **True positives**  (Patients correctly classified as having invasive aspergillosis) | **Prevalence 1%** | 2777 (18) | ⊕⊕⊕⊕  **High** | True positives are important, because patients will be treated with improved survival and spared the invasive diagnostic procedures, however they will suffer the toxicity of the treatment. |
|  | **6** (5 to 8) |  |  |  |
|  | **Prevalence 12%** |  |  |  |
|  | **77** (60 to 92) |  |  |  |
|  | **Prevalence 44%** |  |  |  |
|  | **282** (220 to 339) |  |  |  |
| **False negatives**  (Patients incorrectly classified as not having invasive aspergillosis) | **Prevalence 1%** | 2777 (18) |  | False negatives are important, because of missed diagnosis and either will not be treated or treatment will be delayed with uncertain consequences on mortality. |
|  | **4** (2 to 5) |  |  |  |
|  | **Prevalence 12%** |  |  |  |
|  | **43** (28 to 60) |  |  |  |
|  | **Prevalence 44%** |  |  |  |
|  | **158** (101 to 220) |  |  |  |
|  |  |  |  |  |
| **True negatives**  (Patients correctly classified as not having invasive aspergillosis) | **Prevalence 1%** | 2777 (18) | ⊕⊕⊕⊕  **High** | True negatives are important, because patients will not be treated unnecessarily, will be spared the toxic effects of treatment, and will be reassured. |
|  | **941** (901 to 960) |  |  |  |
|  | **Prevalence 12%** |  |  |  |
|  | **836** (801 to 854) |  |  |  |
|  | **Prevalence 44%** |  |  |  |
|  | **532** (510 to 543) |  |  |  |
| **False positives** (Patients incorrectly classified as having invasive aspergillosis) | **Prevalence 1%** | 2777 (18) |  | False positives are important, because patients will be treated unnecessarily and many will be exposed to nephrotoxic drugs, also because of a possibly delayed diagnosis and treatment of true cause of symptoms. |
|  | **50** (30 to 89) |  |  |  |
|  | **Prevalence 12%** |  |  |  |
|  | **44** (26 to 79) |  |  |  |
|  | **Prevalence 44%** |  |  |  |
|  | **28** (17 to 50) |  |  |  |
| **CI:** Confidence interval; **ODI:** Optical density index; **EORTC:** European Organization for Research and Treatment of Cancer; **MSG:** Mycoses Study Group | | | | |
| Leeflang M, Debets-Ossenkopp Y, Visser C, Scholten R, Hooft L, Bijlmer H, et al. Galactomannan detection for invasive aspergillosis in immunocompromized patients. Cochrane Database of Systematic Reviews. 2008; 4 (Art. No.: CD007394). | | | | |
| **Footnotes:**  ^1^ One study that included only patients with HIV/AIDS was excluded, because this patient group and setting differed from the other included patient groups in such a way that authors of the review regarded the study population as not being representative.  ^2^ A diagnostic test for invasive aspergillosis (IA) needs to be not too invasive or a burden to immunocompromised patients. The galactomannan ELISA test on a serum specimen is less burdensome than the current reference standard.  ^3^ A true reference standard is autopsy combined with culture from autopsy specimens; a clinical reference standard is composed of EORTC/MSG clinical and histological criteria.  ^4^ Estimates of prevalence of IA were based on the median and range of values in included studies. | | | | |

**SoF Table – 2 prevalence estimates (sens and spec in header)**

| **Galactomannan ELISA for the diagnosis of invasive aspergillosis** | | | | | | |
| --- | --- | --- | --- | --- | --- | --- |
| **Patients or population:** immunocompromized patients, mostly hematology patients^1^ **Settings:** mainly hematology or cancer departments, mainly inpatients **New Test:** commercial Platelia® sandwich ELISA detecting galactomannan in serum^2^ \| **Cut-off value:** 1.5 ODI  **Reference Test:** composite of EORTC/MSG clinical and histological criteria^3^ **\| Threshold:** Proven or probable invasive aspergillosis  **Pooled Sensitivity:** 0.64 (95% CI 0.50 to 0.77) \| **Pooled Specificity:** 0.95 (95% CI 0.91 to 0.97) | | | | | | |
| **Test Result** | **Number of results**  **per 1000 patients tested**  **(95% CI)** | | | **Number of participants (studies)** | **Quality of the Evidence**  **(GRADE)** | **Comments** |
|  | Assumed numbers with galactomannan ELISA compared to reference test (EORTC/MSG criteria) | | |  |  |  |
|  | **Prevalence 2%^4^** | **Prevalence 12%^4^** | |  |  |  |
| **True positives** | **6** (5 to 8) | | **77** (60 to 92) | 2777 (18) |   **High^5^** | True positives are important, because patients will be treated with improved survival and spared the invasive diagnostic procedures, however they will suffer the toxicity of the treatment. |
| **False negatives** | **4** (2 to 5) | | **43** (28 to 60) |  |  | False negatives are important, because of missed diagnosis and either will not be treated or treatment will be delayed with uncertain consequences on mortality. |
| **True negatives** | **941** (901 to 960) | | **836** (801 to 854) | 2777 (18) |   **High^5^** | True negatives are important, because patients will not be treated unnecessarily, will be spared the toxic effects of treatment, and will be reassured. |
| **False positives** | **50** (30 to 89) | | **44** (26 to 79) |  |  | False positives are important, because patients will be treated unnecessarily and many will be exposed to nephrotoxic drugs, also because of a possibly delayed diagnosis and treatment of true cause of symptoms. |
| **CI:** Confidence interval; **ODI:** Optical density index; **EORTC:** European Organization for Research and Treatment of Cancer; **MSG:** Mycoses Study Group | | | | | | |
| Leeflang M, Debets-Ossenkopp Y, Visser C, Scholten R, Hooft L, Bijlmer H, et al. Galactomannan detection for invasive aspergillosis in immunocompromized patients. Cochrane Database of Systematic Reviews. 2008; 4 (Art. No.: CD007394). | | | | | | |
| **Footnotes:**  ^1^ One study that included only patients with HIV/AIDS was excluded, because this patient group and setting differed from the other included patient groups in such a way that authors of the review regarded the study population as not being representative.  ^2^ A diagnostic test for invasive aspergillosis (IA) needs to be not too invasive or a burden to immunocompromised patients. The galactomannan ELISA test on a serum specimen is less burdensome than the current reference standard.  ^3^ A true reference standard is autopsy combined with culture from autopsy specimens; a clinical reference standard is composed of EORTC/MSG clinical and histological criteria.  ^4^ Estimates of prevalence of IA were based on the median and range of values in included studies.  ^5^ Included studies were valid diagnostic accuracy studies in patients with diagnostic uncertainty and direct comparison of test results with an appropriate reference standard. Quality was not downgraded based on the GRADE criteria. | | | | | | |

| **Galactomannan ELISA for the diagnosis of invasive aspergillosis** | | | | | | |  |  |  |
| --- | --- | --- | --- | --- | --- | --- | --- | --- | --- |
| **Patients or population:** immunocompromized patients, mostly hematology patients^1^ **Settings:** mainly hematology or cancer departments, mainly inpatients **New Test:** commercial Platelia® sandwich ELISA detecting galactomannan in serum^2^  **Cut-off value:** 1.5 ODI  **Reference Test:** composite of EORTC/MSG clinical and histological criteria^3^  **Threshold:** Proven or probable invasive aspergillosis | | | | | | |  |  |  |
| **Test Property**  **(95% CI)** | **Number of participants (studies)** | **Quality**  **of the Evidence**  **(GRADE)** | | **Test Result** | **Number of results**  **per 1000 patients tested**  **(95% CI)**  Assumed numbers with galactomannan ELISA compared to reference test (EORTC/MSG criteria) | | | **Comments** | |
|  |  |  |  |  | **Prevalence 2%^4^** | **Prevalence 12%^4^** | |  |  |
| **Sensitivity**  0.64  (0.50 to 0.77) | 2777  (18) | ⊕⊕⊕⊕  High^5^ | True positives | | **6** (5 to 8) | **77** (60 to 92) | | True positives are important, because patients will be treated with improved survival and spared the invasive diagnostic procedures, however they will suffer the toxicity of the treatment. | |
|  |  |  | False negatives | | **4** (2 to 5) | **43** (28 to 60) | | False negatives are important, because of missed diagnosis and either will not be treated or treatment will be delayed with uncertain consequences on mortality. | |
| **Specificity**  0.95  (0.91 to 0.97) | 2777  (18) | ⊕⊕⊕⊕  High^5^ | True negatives | | **941** (901 to 960) | **836** (801 to 854) | | True negatives are important, because patients will not be treated unnecessarily, will be spared the toxic effects of treatment, and will be reassured. | |
|  |  |  | False positives | | **50** (30 to 89) | **44** (26 to 79) | | False positives are important, because patients will be treated unnecessarily and many will be exposed to nephrotoxic drugs, also because of a possibly delayed diagnosis and treatment of true cause of symptoms. | |
| **CI:** Confidence interval; **ODI:** Optical density index; **EORTC:** European Organization for Research and Treatment of Cancer; **MSG:** Mycoses Study Group | | | | | | | | | |
| Leeflang M, Debets-Ossenkopp Y, Visser C, Scholten R, Hooft L, Bijlmer H, et al. Galactomannan detection for invasive aspergillosis in immunocompromized patients. Cochrane Database of Systematic Reviews. 2008; 4 (Art. No.: CD007394). | | | | | | | | | |
| **Footnotes:**  ^1^ One study that included only patients with HIV/AIDS was excluded, because this patient group and setting differed from the other included patient groups in such a way that authors of the review regarded the study population as not being representative.  ^2^ A diagnostic test for invasive aspergillosis (IA) needs to be not too invasive or a burden to immunocompromised patients. The galactomannan ELISA test on a serum specimen is less burdensome than the current reference standard.  ^3^ A true reference standard is autopsy combined with culture from autopsy specimens; a clinical reference standard is composed of EORTC/MSG clinical and histological criteria.  ^4^ Estimates of prevalence of IA were based on the median and range of values in included studies.  ^5^ Included studies were valid diagnostic accuracy studies in patients with diagnostic uncertainty and direct comparison of test results with an appropriate reference standard. Quality was not downgraded based on the GRADE criteria. | | | | | | | | | |

**SoF Table – 2 prevalence estimates (sens and spec in table)**

**SoF Table – 2 prevalence estimates (sens and spec in table), clinical scenario**

| **Galactomannan ELISA for the diagnosis of invasive aspergillosis** | | | | |
| --- | --- | --- | --- | --- |
| **Patients or population:** immunocompromized patients, mostly hematology patients^1^ **Settings:** mainly hematology or cancer departments, mainly inpatients **New Test:** commercial Platelia® sandwich ELISA detecting galactomannan in serum^2^ \| **Cut-off value:** 1.5 ODI  **Reference Test:** composite of EORTC/MSG clinical and histological criteria^3^ **\| Threshold:** Proven or probable invasive aspergillosis | | | | |
| **Test Result** | **Number of results per 1000 patients tested**  **(95% CI)^4^** | | **Number of participants (studies)** | **Quality of the Evidence**  **(GRADE)** |
|  | **Prevalence 20 per 1000^5^ :**  Which is typically seen in adults undergoing transplant, no neutropenic patients. | **Prevalence 440 per 1000^5^ :**  Which is typically seen in adults with hematological disorders that were neutropenic, underwent chemotherapy, had persistent fever despite antibiotics, acute graft versus host disease, or received corticosteroids. |  |  |
| *Sensitivity (95% CI)*: **0.64** (0.50 to 0.77) | | | | |
| **True positives** | **13** (9 to 15) | **282** (220 to 339) | 2777 (18) | ⊕⊕⊕⊕  **High^6^** |
| **False negatives** | **7** (4 to 10) | **158** (101 to 220) |  |  |
| *Specificity (95% CI):*  **0.95** (0.91 to 0.97) | | | | |
| **True negatives** | **931** (901 to 960) | **532** (510 to 543) | 2777 (18) | ⊕⊕⊕⊕  **High^6^** |
| **False positives** | **49** (30 to 89) | **28** (17 to 50) |  |  |
| **CI:** Confidence interval; **ODI:** Optical density index; **EORTC:** European Organization for Research and Treatment of Cancer; **MSG:** Mycoses Study Group | | | | |
| Leeflang M, Debets-Ossenkopp Y, Visser C, Scholten R, Hooft L, Bijlmer H, et al. Galactomannan detection for invasive aspergillosis in immunocompromized patients. Cochrane Database of Systematic Reviews. 2008; 4 (Art. No.: CD007394). | | | | |
| **Footnotes:**  ^1^ One study that included only patients with HIV/AIDS was excluded, because this patient group and setting differed from the other included patient groups in such a way that authors of the review regarded the study population as not being representative.  ^2^ A diagnostic test for invasive aspergillosis (IA) needs to be not too invasive or a burden to immunocompromised patients. The galactomannan ELISA test on a serum specimen is less burdensome than the current reference standard.  ^3^ A true reference standard is autopsy combined with culture from autopsy specimens; a clinical reference standard is composed of EORTC/MSG clinical and histological criteria.  ^4^ Assumed numbers with galactomannan ELISA compared to reference test (EORTC/MSG criteria).  ^5^ Estimates of prevalence of IA were based on the median and range of values in included studies.  ^6^ Included studies were valid diagnostic accuracy studies in patients with diagnostic uncertainty and direct comparison of test results with an appropriate reference standard. Quality was not downgraded based on the GRADE criteria. | | | | |

| **SoF Table – 2 prevalence estimates (sens and spec in table), no clinical scenario** | | | |
| --- | --- | --- | --- |
| **Galactomannan ELISA for the diagnosis of invasive aspergillosis** | | | |
| **Patients or population:** immunocompromized patients, mostly hematology patients^1^ **Settings:** mainly hematology or cancer departments, mainly inpatients **New Test:** commercial Platelia® sandwich ELISA detecting galactomannan in serum^2^ \| **Cut-off value:** 1.5 ODI  **Reference Test:** composite of EORTC/MSG clinical and histological criteria^3^ **\| Threshold:** Proven or probable invasive aspergillosis | | | |
| **Test Result** | **Number of results per 1000 patients tested**  **(95% CI)** | **Number of participants (studies)** | **Quality of the Evidence**  **(GRADE)** |
|  | Assumed numbers with galactomannan ELISA compared to reference test (EORTC/MSG criteria) |  |  |
|  | **Prevalence 20 per 1000^4^** |  |  |
| *Sensitivity (95% CI)*: **0.64** (0.50 to 0.77) | | | |
| **True positives** | **13** (9 to 15) | 2777 (18) | ⊕⊕⊕⊕  **High^5^** |
| **False negatives** | **7** (4 to 10) |  |  |
| *Specificity (95% CI):*  **0.95** (0.91 to 0.97) | | | |
| **True negatives** | **931** (901 to 960) | 2777 (18) | ⊕⊕⊕⊕  **High^5^** |
| **False positives** | **49** (30 to 89) |  |  |
| **CI:** Confidence interval; **ODI:** Optical density index; **EORTC:** European Organization for Research and Treatment of Cancer; **MSG:** Mycoses Study Group | | | |
| Leeflang M, Debets-Ossenkopp Y, Visser C, Scholten R, Hooft L, Bijlmer H, et al. Galactomannan detection for invasive aspergillosis in immunocompromized patients. Cochrane Database of Systematic Reviews. 2008; 4 (Art. No.: CD007394). | | | |
| **Footnotes:**  ^1^ One study that included only patients with HIV/AIDS was excluded, because this patient group and setting differed from the other included patient groups in such a way that authors of the review regarded the study population as not being representative.  ^2^ A diagnostic test for invasive aspergillosis (IA) needs to be not too invasive or a burden to immunocompromised patients. The galactomannan ELISA test on a serum specimen is less burdensome than the current reference standard.  ^3^ A true reference standard is autopsy combined with culture from autopsy specimens; a clinical reference standard is composed of EORTC/MSG clinical and histological criteria.  ^4^ Estimates of prevalence of IA were based on the median and range of values in included studies.  ^5^ Included studies were valid diagnostic accuracy studies in patients with diagnostic uncertainty and direct comparison of test results with an appropriate reference standard. Quality was not downgraded based on the GRADE criteria. | | | |

Table 1: DTA SoF table based on test results (TP, FP and TN, FN)

| **Abdominal ultrasound for the diagnosis of pancreatic cancer** | | | | |
| --- | --- | --- | --- | --- |
| **Patients or population:** symptomatic patients in primary care with suspicion of pancreatic cancer  **Setting:** mainly outpatients **New Test:** abdominal ultrasound^1^  **Reference Test:** endoscopic ultrasound with biopsy^2^  **Threshold:** Proven or probable pancreatic cancer | | | | |
| **Pooled Sensitivity** (95% CI)**:** 0.64 (0.50 to 0.77) \| **Pooled Specificity** (95% CI)**:** 0.95 (0.91 to 0.97) | | | | |
| **Test result** | **Number of results  per 1000 patients tested^3^**  (95% CI) | | **Number of participants  (studies)** | **Quality  of the evidence (GRADE)** |
|  | **Prevalence 20 per 1000^4^:**  Which is typically seen in otherwise healthy adults presenting with symptoms of jaundice, fatigue, pain of the abdomen, and dark urine. | **Prevalence 400 per 1000^4^:**  Which is typically seen in older adults presenting with symptoms of jaundice, fatigue and pain, with a family history of pancreatic cancer, history of chronic pancreatitis, who have diabetes, and are current or past smokers. |  |  |
| Test-Positive | | | 2777  (18 studies) | ⊕⊕⊕⊕  **High^5^** |
| True positives | **13 per 1000**  (9 to 15 per 1000) | **282 per 1000**  (220 to 339 per 1000) |  |  |
| False positives | **49 per 1000**  (30 to 89 per 1000) | **28 per 1000**  (17 to 50 per 1000) |  |  |
| Test-Negative | | |  |  |
| True negatives | **931 per 1000**  (901 to 960 per 1000) | **532 per 1000**  (510 to 543 per 1000) |  |  |
| False negatives | **7 per 1000**  (4 to 10 per 1000) | **158 per 1000**  (101 to 220 per 1000) |  |  |
| **CI:** Confidence interval | | | | |
| **Footnotes:**  ^1^ A diagnostic test for pancreatic cancer needs to be less invasive than the current reference standard and lessen the burden to patients.  ^2^ Endoscopic ultrasound with biopsy as a reference test is a more invasive test, placing higher burden on the patient, with risk of complications such as infection.  ^3^ Assumed numbers with abdominal ultrasound compared to reference test.  ^4^ Estimates of prevalence of pancreatic cancer were based on the median and range of values in included studies.  ^5^ Included studies were valid diagnostic accuracy studies in patients with diagnostic uncertainty and direct comparison of test results with an appropriate reference standard. Quality was not downgraded based on the GRADE criteria. | | | | |

Table 2: DTA SoF table based on disease status (TP, FN and TN, FP)

| **Abdominal ultrasound for the diagnosis of pancreatic cancer** | | | | |
| --- | --- | --- | --- | --- |
| **Patients or population:** symptomatic patients in primary care with suspicion of pancreatic cancer  **Setting:** mainly outpatients **New Test:** abdominal ultrasound1  **Reference Test:** endoscopic ultrasound with biopsy2  **Threshold:** Proven or probable pancreatic cancer | | | | |
| **Pooled Sensitivity (95% CI):** 0.64 (0.50 to 0.77) \| **Pooled Specificity (95% CI):** 0.95 (0.91 to 0.97) | | | | |
| **Test result** | **Number of results  per 1000 patients tested^3^**  (95% CI) | | **Number of participants  (studies)** | **Quality  of the evidence (GRADE)** |
|  | **Prevalence 20 per 1000^4^:**  Which is typically seen in otherwise healthy adults presenting with symptoms of jaundice, fatigue, pain of the abdomen, and dark urine. | **Prevalence 400 per 1000^4^:**  Which is typically seen in older adults presenting with symptoms of jaundice, fatigue and pain, with a family history of pancreatic cancer, history of chronic pancreatitis, who have diabetes, and are current or past smokers. |  |  |
| Test-Positive | | | 2777  (18 studies) | ⊕⊕⊕⊕  **High^5^** |
| True positives | **13 per 1000**  (9 to 15 per 1000) | **282 per 1000**  (220 to 339 per 1000) |  |  |
| False positives | **49 per 1000**  (30 to 89 per 1000) | **28 per 1000**  (17 to 50 per 1000) |  |  |
| Test-Negative | | |  |  |
| True negatives | **931 per 1000**  (901 to 960 per 1000) | **532 per 1000**  (510 to 543 per 1000) |  |  |
| False negatives | **7 per 1000**  (4 to 10 per 1000) | **158 per 1000**  (101 to 220 per 1000) |  |  |
| **CI:** Confidence interval | | | | |
| **Footnotes:**  ^1^ A diagnostic test for pancreatic cancer needs to be less invasive than the current reference standard and lessen the burden to patients.  ^2^ Endoscopic ultrasound with biopsy as a reference test is a more invasive test, placing higher burden on the patient, with risk of complications such as infection.  ^3^ Assumed numbers with abdominal ultrasound compared to reference test.  ^4^ Estimates of prevalence of pancreatic cancer were based on the median and range of values in included studies.  ^5^ Included studies were valid diagnostic accuracy studies in patients with diagnostic uncertainty and direct comparison of test results with an appropriate reference standard. Quality was not downgraded based on the GRADE criteria. | | | | |

Table 3: DTA SoF table based on pre- and post-test probability

| **Abdominal ultrasound for the diagnosis of pancreatic cancer** | | | | | |
| --- | --- | --- | --- | --- | --- |
| **Patients or population:** symptomatic patients in primary care with suspicion of pancreatic cancer  **Setting:** mainly outpatients **New Test:** abdominal ultrasound^1^  **Reference Test:** endoscopic ultrasound with biopsy^2^  **Threshold:** Proven or probable pancreatic cancer | | | | | |
| **Pooled Sensitivity** (95% CI)**:** 0.64 (0.50 to 0.77) \| **Pooled Specificity** (95% CI)**:** 0.95 (0.91 to 0.97) | | | | | |
| **Pre-test  probability^3^** | **Test result** | **Post-test probability^4^**  (95% CI) | **Likelihood ratio (LR)**  (95% CI) | **Number  of participants (studies)** | **Quality  of the evidence (GRADE)** |
| Low Probability | | | | 2777  (18 studies) | ⊕⊕⊕⊕  **High^5^** |
| 2%  - - - - - - - - - - - - - - - - -  Which is typically seen in otherwise healthy adults presenting with symptoms of jaundice, fatigue, pain of the abdomen, and dark urine. | + | **21%**  (17% to 26%) | **+ LR = 13**  (10 to 17) |  |  |
|  | – | **1%**  (1% to 1%) | **– LR = 0.38**  (0.27 to 0.54) |  |  |
| High Probability | | | |  |  |
| 44%  - - - - - - - - - - - - - - - - -  Which is typically seen in older adults presenting with symptoms of jaundice, fatigue and pain, with a family history of pancreatic cancer, history of chronic pancreatitis, who have diabetes, and are current or past smokers. | + | **91%**  (89% to 93%) | **+ LR = 13**  (10 to 17) |  |  |
|  | – | **23%**  (22% to 24%) | **– LR = 0.38**  (0.27 to 0.54) |  |  |
| **CI:** Confidence interval | | | | | |
| **Footnotes:**  ^1^ A diagnostic test for pancreatic cancer needs to be less invasive than the current reference standard and lessen the burden to patients.  ^2^ Endoscopic ultrasound with biopsy as a reference test is a more invasive test, placing higher burden on the patient, with risk of complications such as infection.  ^3^ Pre-test probability of pancreatic cancer was selected based on the median and range of prevalence values in included studies.  ^4^ Percentages are rounded to the nearest integer.  ^5^ Included studies were valid diagnostic accuracy studies in patients with diagnostic uncertainty and direct comparison of test results with an appropriate reference standard. Quality was not downgraded based on the GRADE criteria. | | | | | |
